# The planctomycete *Stieleria maiorica* Mal15^T^ employs stieleriacines to alter the species composition in marine biofilms

**DOI:** 10.1038/s42003-020-0993-2

**Published:** 2020-06-12

**Authors:** Nicolai Kallscheuer, Olga Jeske, Birthe Sandargo, Christian Boedeker, Sandra Wiegand, Pascal Bartling, Mareike Jogler, Manfred Rohde, Jörn Petersen, Marnix H. Medema, Frank Surup, Christian Jogler

**Affiliations:** 1 Department of Microbiology, Radboud University, Nijmegen, The Netherlands; 2 Leibniz Institute DSMZ, Braunschweig, Germany; 3 Helmholtz Centre for Infection Research, Braunschweig, Germany; 4 German Centre for Infection Research (DZIF), Braunschweig, Germany; 5 Institute for Biological Interfaces 5, Karlsruhe Institute of Technology, Eggenstein-Leopoldshafen, Germany; 6 Central Facility for Microscopy, Helmholtz Centre for Infection Research, Braunschweig, Germany; 7 Bioinformatics Group, Wageningen University, Wageningen, The Netherlands; 8 Department of Microbial Interactions, Friedrich-Schiller University, Jena, Germany

**Keywords:** Biofilms, Small molecules, Water microbiology

## Abstract

Bacterial strains of the phylum Planctomycetes occur ubiquitously, but are often found on surfaces of aquatic phototrophs, e.g. alga. Despite slower growth, planctomycetes are not outcompeted by faster-growing bacteria in biofilms on such surfaces; however, strategies allowing them to compensate for slower growth have not yet been investigated. Here, we identified stieleriacines, a class of *N*-acylated tyrosines produced by the novel planctomycete *Stieleria maiorica* Mal15^T^, and analysed their effects on growth of the producing strain and bacterial species likely co-occurring with strain Mal15^T^. Stieleriacines reduced the lag phase of Mal15^T^ and either stimulated or inhibited biofilm formation of two bacterial competitors, indicating that Mal15^T^ employs stieleriacines to specifically alter microbial biofilm composition. The genetic organisation of the putative stieleriacine biosynthetic cluster in strain Mal15^T^ points towards a functional link of stieleriacine biosynthesis to exopolysaccharide-associated protein sorting and biofilm formation.

## Abstract (web summary)

Kallscheuer, Jeske, Sandargo et al. demonstrate that stieleriacines, a class of *N*-acylated tyrosine derivatives, can alter species abundance in marine biofilms. This study suggests that slow-growing planctomycetes employ stieleriacines to counteract the growth advantage of their competitors.

## Introduction

From a heterotrophic bacterial perspective, the vastness of the sea represents a hostile oligotrophic ‘desert’. In contrast, surfaces of marine macroscopic phototrophs are nutrient-rich ‘oases’, densely packed with all sorts of alluring organic compounds, which can serve as nutrient sources. In aquatic environments, such biotic surfaces represent desirable ecological niches and are thus rapidly occupied by bacterial biofilms^1,2^. Members of the ‘*Roseobacter* group’ are particularly successful in such habitats^3^. For example, *Phaeobacter inhibens* employs *N*-acyl homoserine lactones (AHLs) as quorum sensing signals to initiate biofilm formation^4^. In *P. inhibens*, AHLs trigger the expression of the gene encoding AHL synthase along with other genes that promote biofilm formation. This positive feedback loop is often associated with the production of bioactive small molecules, e.g., the antibiotic tropodithietic acid (TDA) by *P. inhibens*^5,6^.

While *P. inhibens* and other proteobacteria are well known to dominate biotic surfaces, it was found that members of the phylum Planctomycetes can sometimes also be the dominating taxon^7^. Species belonging to the family Pirellulaceae, including the marine model planctomycete *Rhodopirellula baltica*, switch between different lifestyles, in which they either live as motile free-swimming cells or attach to surfaces^8^. Such a complex lifecycle requires control at additional stages beyond canonical transcription factor-based regulation of gene expression. Members of the family Pirellulaceae might thus rely on similar regulatory mechanisms of secreting signalling molecules for enabling cell-to-cell communication as the *Roseobacter* clade and many other microorganisms^9^. Large planctomycetal genomes of up to 12.4 Mb and high numbers of predicted clusters involved in small molecule production are in line with the assumed portfolio of bioactive compounds with potential regulatory activities, in addition to two component systems and extracytoplasmic function sigma factors^10^. Although planctomycetes grow rather slowly compared to competing microorganisms occupying the same ecological niche, they are not outcompeted by their natural competitors. On the contrary, planctomycetes can even account for up to 70% of the bacterial community in certain habitats^7^. The deficit in growth rate is suggested to be compensated by the production of small molecules with antimicrobial properties, while the chemical nature of such molecules remains elusive^11^. The current knowledge gap mainly results from the insufficient number of planctomycetes available as axenic cultures^7^. Recently, we developed an isolation pipeline to obtain novel planctomycetal strains in axenic culture. Basis for the isolation of 79 novel strains was an optimised formulation of cultivation media, supplemented with a blend of carefully titrated antibiotics and fungicides^10^. In Mal15^T^, one of the strains isolated by this strategy, we detected production of novel compounds beloning to the class of *N*-acylated tyrosine derivatives, which might have regulatory activities in this strain. In order to validate our working hypothesis, we performed assays with purified stieleriacine for studying its role on intraspecies and interspecies communication. Additionally, we inferred a putative biosynthetic pathway based on the genome sequence of strain Mal15^T^ obtained during a detailed characterisation of the novel strain.

## Results

### Identification of stieleriacines in cultures of strain Mal15^T^

Given our working hypothesis that planctomycetes are a promising source for novel bioactive small molecules, we continuously extend the current collection of axenic cultures from this phylum^10^. Here, we analysed the novel biofilm-forming strain Mal15^T^ (DSM 100215^T^ = LMG 29790^T^) isolated from sediments on Mallorca island in the Mediterranean Sea. Our phylogenetic analysis suggests that strain Mal15^T^ belongs to a novel genus and species in the family Pirellulaceae. Hence, we propose the name *Stieleria maiorica* gen. nov., sp. nov. for the novel isolate. A detailed characterisation of the novel strain is presented in the Supplementary Information.

**Fig. 1:**
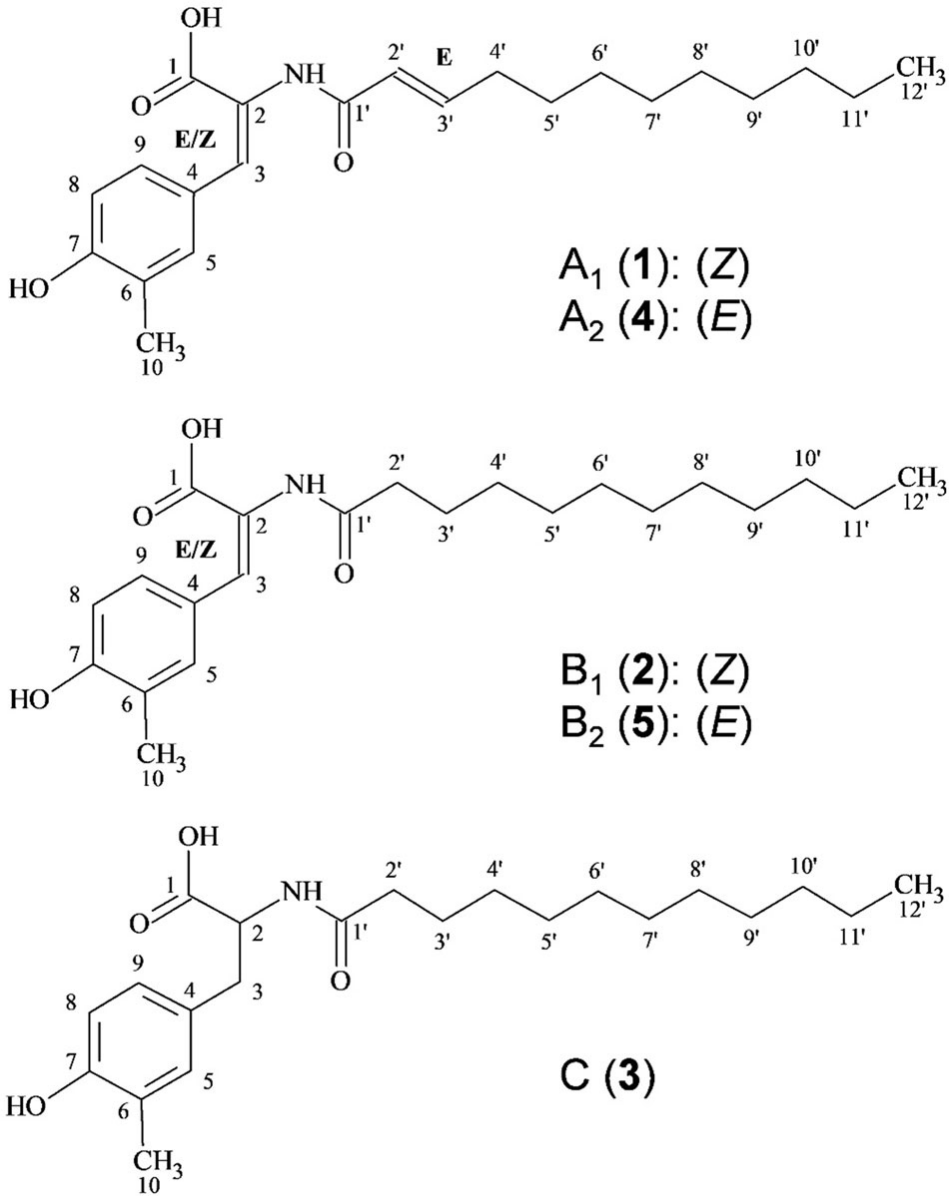
Stieleriacines. Structures of stieleriacines A_1_, A_2_ (upper panel), B_1_, B_2_ (middle panel) and C (lower panel) are depicted.

During metabolite analysis in culture supernatants of strain Mal15^T^, we found that it produces a distinct class of small molecules belonging to the group of long-chain *N*-acyl tyrosine derivatives, which we named stieleriacines (in accordance with the proposed genus name *Stieleria*). Stieleriacines are composed of a lauric acid or *trans*-2-dodecenoic acid moiety as fatty acid residue, which is ligated to a dehydrotyrosine derivative additionally *C*-methylated in *meta*-position of its aromatic ring (Fig. 1). Stieleriacines were found to be produced during laboratory-scale shaking flask cultivations of axenic Mal15^T^ cultures, indicating that their production does not require presence of other microorganisms as an external stimulus.

### Analysis of in vitro and in vivo effects of stieleriacine A_1_

**Fig. 2:**
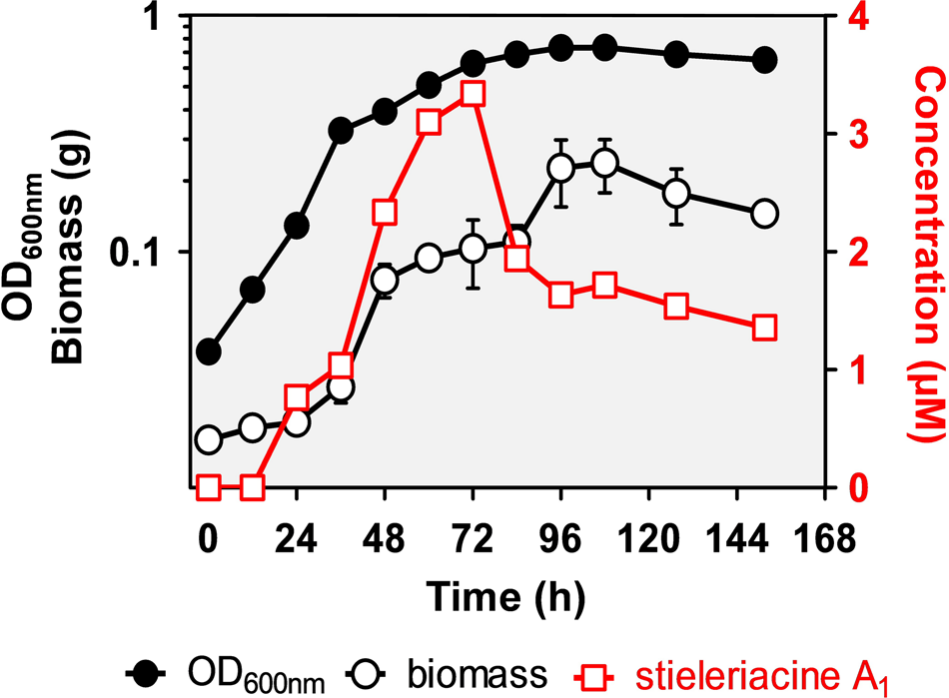
Stieleriacine A_1_ production in relation to growth of strain Mal15^T^. Triplicates of Mal15^T^ cultures were harvested every 12 h for five days, followed by harvesting every 16 h for two days. The optical density at 600 nm (OD_600nm_), the biomass dry weight and the stieleriacine A_1_ titre in the cell supernatant were quantified. Error bars indicate the standard deviation. Where error bars are not visible, they are shorter than the size of the symbols.

**Fig. 3:**
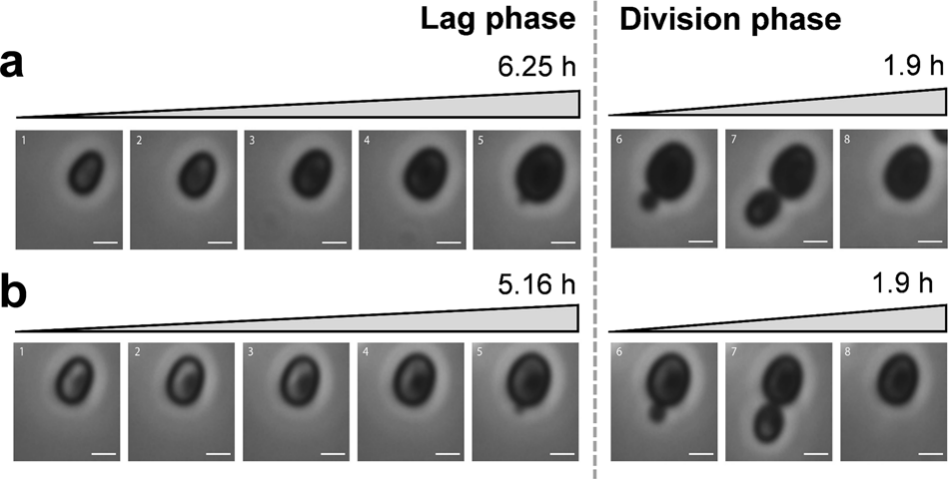
Effect of stieleriacine A_1_ on growth of strain Mal15^T^. **a** Time-lapse series of untreated Mal15^T^ cells during the 6.25 h lag-phase (1–5) and after initialisation of cell division and entrance into the exponential phase (6–8). The division as such from budding to daughter cell release required 1.9 h. **b** The lag phase of Mal15^T^ cells was significantly shorter (1–5: 5.16 h) once treated with 1.34 µM stieleriacine A_1_, while the division as such lasted 1.9 h as well (6–8); (untreated: 6.25 h (**a**), treated: 5.16 h (**b**), *p* = 0.0058). The scale bar is 1 µm.

In previous studies, it has been shown that *N*-acyl amino acids can display antimicrobial activities against various bacteria^12–17^, while *N*-acylated tyrosine derivatives were shown to inhibit the enzyme tyrosinase^18^. Employing in vivo and in vitro assays with purified stieleriacine A_1_, the major stieleriacine produced by strain Mal15^T^, we could show that these compounds exhibit only moderate antimicrobial activity against Gram-positive bacteria (Supplementary Table 1) and negligible inhibition of tyrosinase, indicating that neither of the two effects reflects the major function of stieleriacines. To get first hints on the natural function of stieleriacine A_1_, we thus applied time-lapse microscopy coupled to microfluidics as experimental strategy to allow observation of individual cells over time spans of several hours to days. Such unusual approaches were necessary since most of the canonical biofilm formation assays did not work for planctomycetes. Mal15^T^ cells were treated with 1.34 µM stieleriacine A_1_, a ‘physiological’ concentration found to be produced in the mid-exponential phase during laboratory-scale cultivation of the strain (Fig. 2). The natural production of stieleriacines by strain Mal15^T^ can be neglected in this experimental setup as a constant medium flow ensures that compounds secreted by the strain are immediately flushed away. We found that presence of stieleriacine A_1_ significantly reduced the duration of the lag phase (5.2 h) compared to untreated Mal15^T^ cells (6.3 h; *p* = 0.0058) (Fig. 3, Supplementary Table 2, Supplementary Movie 1 and 2). This observation points towards a bioactivity to the advantage of its producer, however, we did not exclude any additional inhibitory effect on competing microbes at this stage.

**Fig. 4:**
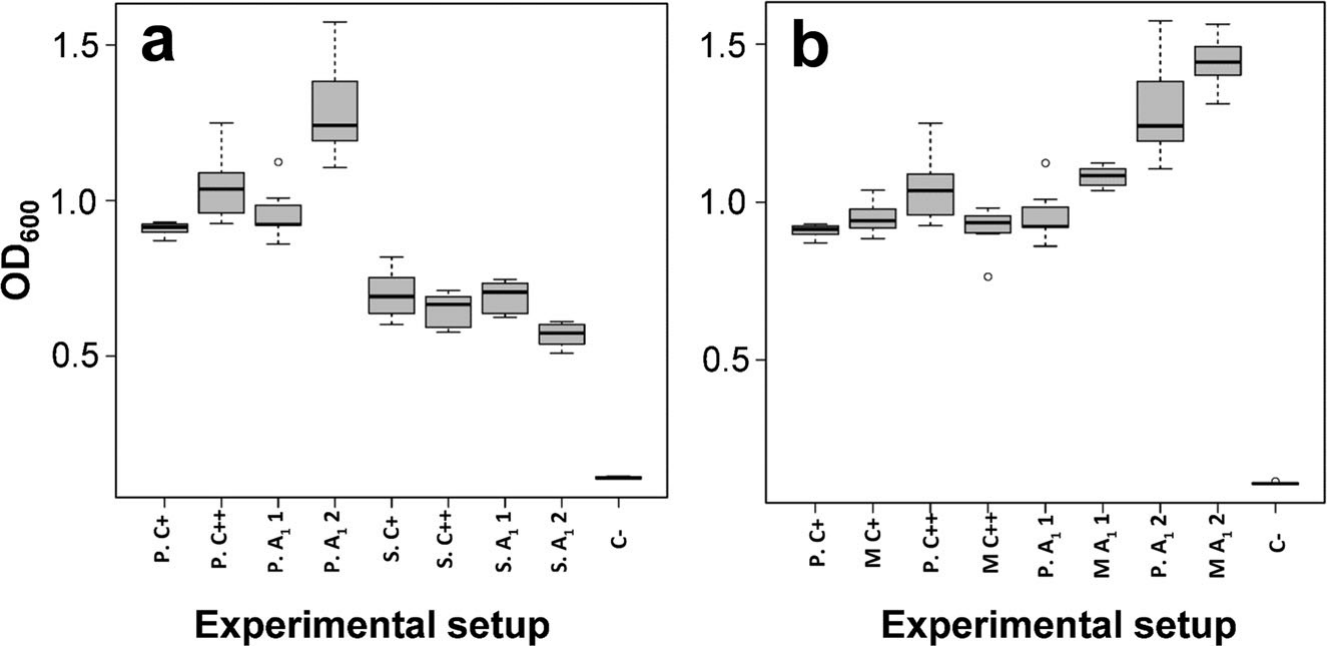
Box plot of biofilm formation capacity for cells treated with stieleriacine A_1_. **a** Box plot of biofilm formation capacity of *P. inhibens* (P) and *S. dubius* (S). **b** Box plot of biofilm formation capacity of *P. inhibens* (P) compared to *P. inhibens* Δ*luxR* transposon mutant (M). Experimental setup: **P.C**+: *P. inhibens* in MB medium; **P.C**++: *P. inhibens* in MB with acetone; **P.A**_**1**_**1**: *P. inhibens* in MB with stieleriacine A_1_ 1.34 µM in acetone; **P.A**_**1**_**2**: *P. inhibens* in MB with stieleriacine A_1_ 134 µM in acetone; **S.C**+: *S. dubius* in MB medium; **S.C**++: *S. dubius* in MB medium with acetone; **SA**_**1**_**1**: *S. dubius* in MB with stieleriacine A_1_ 1.34 µM in acetone; **S.A**_**1**_**2**: *S. dubius* in MB with stieleriacine A_1_ 134 µM in acetone; **M.C**+: Δ*luxR* transposon mutant in MB medium; **P.C**++: Δ*luxR* transposon mutant in MB with acetone; **MA**_**1**_**1**: Δ*luxR* transposon mutant in MB with stieleriacine A_1_ 1.34 µM in acetone; **MA**_**1**_**2**: Δ*luxR* transposon mutant in MB with stieleriacine A_1_ 134 µM in acetone; **C−**: MB medium. For each experiment, four biological replicates and two technical replicates were performed. The minimum and maximum value, sample median, and the first and third quartiles are shown (box-and-whisker plot).

The artificial laboratory cultivation conditions and the tested concentration of stieleriacine A_1_ probably only insufficiently reflect natural conditions in seawater, in which densities of planktonic Mal15^T^ cells are much lower. From an ecological perspective, stieleriacine production by individual swarming cells or smaller aggregates appears irrational as the secreted molecules are immediately diluted to inactive concentrations. We hypothesised that sufficient stieleriacine concentrations can only be reached by larger numbers of cells inhabiting a local environment, such as a biofilm with a rather gel-like texture and that stieleriacines might thus play a more important role in such micro-environments. Following this argumentation, we were curious to test for a potential effect of stieleriacine A_1_ on biofilm formation capabilities of natural competitors of strain Mal15^T^. We chose two members of the ‘*Roseobacter* group’, *P. inhibens* DSM 17395 and *Sulfitobacter dubius* DSM 16472^T^, which were shown to co-occur with species of the familiy Pirellulaceae^19,20^. The biofilm assays were performed with isolated stieleriacine A_1_ in absence of Mal15^T^ cells to exclude any additional effects, which might e.g. be caused by other natural compounds produced by strain Mal15^T^. Unexpectedly, application of stieleriacine A_1_ led to an increased biofilm formation of *P. inhibens* (+35%, *p* < 0.001), but reduced biofilm formation of *S. dubius* (−15%, *p* < 0.001) (Fig. 4a). This effect was observed when 134 µM stieleriacine A_1_ was used, a 100-fold higher concentration than in the microfluidics experiment performed with strain Mal15^T^. The biofilm assay required empirical optimisation of the stieleriacine A_1_ concentration; since we were aware that concentrations in liquid cultures might not properly reflect local concentrations in biofilms, in which cells are in very close proximity and local metabolite concentrations are thus considerably higher. We decided to follow the empirical optimisation approach of the stieleriacine A_1_ concentration to reconstruct the natural conditions in the biofilm as good as possible. Natural local concentrations of stieleriacines in biofilms are difficult to assess and largely dependent on size and microbial community composition of the biofilm. The observed stimulating effect of stieleriacine A_1_ on *P. inhibens* was surprising and was thus analysed further. To this end, we tested for a potential interaction of stieleriacine A_1_ with the AHL-dependent quorum sensing system in *P. inhibens*, in particular the AHL-responsive transcriptional regulator protein LuxR^21^. Mutant analysis with a *luxR*-deficient *P. inhibens* strain (Supplementary Fig. 1) demonstrated that the positive effect on biofilm formation is largely independent from LuxR, indicating that stieleriacine A_1_ does not directly interfere with the quorum sensing system of *P. inhibens* (Fig. 4b). But, why should strain Mal15^T^ produce a molecule that stimulates biofilm formation of its natural competitor *P. inhibens*, while reducing the fitness of its other competitor *S. dubius*? The reason possibly relates to production of TDA^22^. Promoting the biofilm formation of *P. inhibens* would in return lead to increased production of AHLs and TDA^23,24^. This benefits strain Mal15^T^ in two ways: (i) Mal15^T^ turned out to be resistant against TDA while other competitors are not. (ii) TDA production reduces the growth speed of *P. inhibens* approximately by 41% due to the increased metabolic burden^25^ and may ensure that strain Mal15^T^ is not outcompeted. Thus, we suggest that strain Mal15^T^ ‘invites’ *P. inhibens* via stieleriacine production to join the biofilm and to produce TDA in order to challenge other faster growing competitors. In contrast, biofilm formation of *S. dubius* is reduced by stieleriacine A_1_ as this bacterium does not produce TDA and is in that regard useless for strain Mal15^T^. The hypothesis suggests that planctomycetes actively shape the biofilm community to gain advantages employing a novel type of natural products: stieleriacines. Our results thus point towards the capability of certain slow-growing bacteria to mediate colonisation of their surrounding by production of small molecules. The hypothesis fits to the observation that slow-growing planctomycetes can dominate biofilms on competitive marine surfaces^7^.

### Genome analysis identifies a putative stieleriacine biosynthesis pathway

**Fig. 5:**
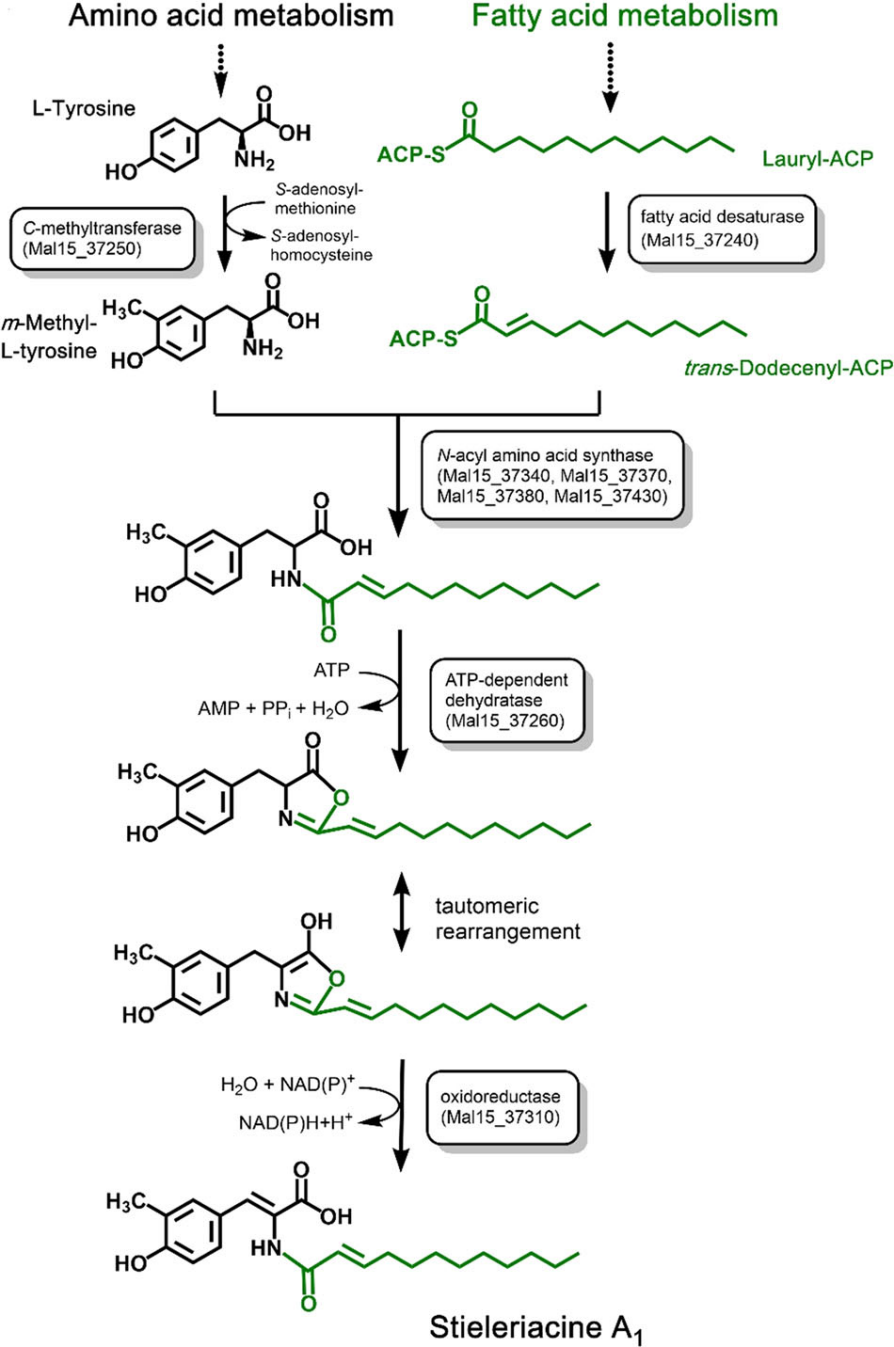
Proposed biosynthesis pathway of stieleriacines. The proposed stieleriacine biosynthesis pathway starting from l-tyrosine and lauryl-ACP is depicted. The locus tags of genes coding for candidate enzymes participating in the pathway are shown in boxes. ACP acyl carrier protein.

**Fig. 6:**
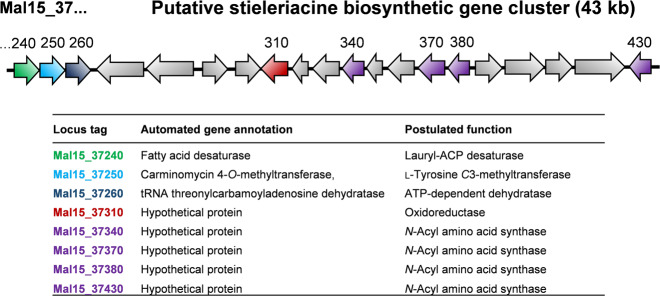
The putative stieleriacine biosynthetic gene cluster in strain Mal15^T^. Genes with a predicted role in the stieleriacine biosynthesis cluster are shown in different colours. For these genes the automated gene annotation and the postulated function are shown in the table. The complete list of automated gene annotations of the cluster can be found in Supplementary Table 3.

Although microbial synthesis of *N*-acyl tyrosine molecules has been reported in several recent studies^18,26,27^, the genetic basis for biosynthesis has not been elucidated in greater detail. The key enzyme for stieleriacine biosynthesis in strain Mal15^T^ is likely an *N*-acyl amino acid synthase (NAS) (Fig. 5). We sequenced the 9.9 Mb genome of strain Mal15^T^ and identified a cluster (locus tags Mal15_37240 to Mal15_37430, Fig. 6), which contains not only four putative NAS-encoding genes, but all genes required for a postulated stieleriacine biosynthesis pathway. The NAS-encoding genes (Mal15_37340, Mal15_37370, Mal15_37380 and Mal15_37430) were originally annotated as hypothetical proteins, but were identified as candidates by BLASTp and InterPro scan. The four enzymes might have different substrate spectra, which can explain the production of five slightly different stieleriacines in strain Mal15^T^ (Fig. 1). However, it should not be excluded that the strain is capable of producing other *N*-acyl amino acids. The *C*-methylation in *meta*-position of the aromatic ring and an uncommon double bond in the α,β-position of the tyrosine moiety were identified as characteristic molecular features separating stieleriacines from other *N*-acyl tyrosines identified so far^18,26^. In the biosynthetic cluster, Mal15_37250 encodes a putative methyltransferase similar to the l-tyrosine *C*3-methyltransferase SfmM2 of *Streptomyces lavendulae*. This enzyme catalyses an early step in saframycin biosynthesis^28^ identical to the reaction required during synthesis of stieleriacines (Fig. 5). The gene encoded immediately downstream, Mal15_37560, codes for a putative tRNA threonylcarbamoyladenosine dehydratase. When only taking the putative protein annotation into consideration, involvement in stieleriacine biosynthesis appears rather unlikely. However, the substrate of the dehydratase (tRNA threonylcarbamoyladenosine) strongly resembles the amide bond obtained after the *N*-acylation reaction in the postulated stieleriacine pathway. The catalysed lactonization and dehydration reaction would give rise to a cyclic pathway intermediate, in which the remaining double bond can be introduced by a coupled oxidation and hydrolysis reaction induced by a keto-enol tautomeric rearrangement (Fig. 5). This reaction may be catalysed by the putative oxidoreductase encoded by Mal15_37310. Other proteins in the cluster code for a putative transcriptional regulator or putative transporters (Supplementary Table 3), which could have additional function for regulation of the pathway and product export, however, we had no clear indication for this based on published information. Mal15_37240, encoding a putative fatty acid desaturase, is likely involved in the introduction of the double bond in the fatty acid part of the molecule (Fig. 5), however it remains to be elucidated whether this reaction takes place before or after the *N*-acylation.

## Discussion

In this study, we isolated and characterised *Stieleria maiorica* Mal15^T^, which represents a novel species and genus within the family Pirellulaceae, order Pirellulales, class Planctomycetia, phylum Planctomycetes. The strain was shown to produce *N*-acylated tyrosine deriatives, which we designated stieleriacines and which belong to the class of *N*-acylated amino acids. In previous studies, compounds of this class showed antimicrobial activities against various bacteria^12–17^ and probably act as signalling molecules mediating intraspecies and interspecies communication^4,15,29^. *N*-acylated tyrosine derivatives isolated from the marine γ-proteobacterium *Thalassotalea* sp. PP2-459 (Alteromonadales) were also shown to inhibit the enzyme tyrosinase^18^. Recently, an *N*-acyl tyrosine congener harbouring an uncommon α-methyl group in the tyrosine moiety was identified in *Alteromonas* sp. RKMC-009 isolated from the surface of a marine sponge^26^, while its natural function remains to be elucidated. Stieleriacine A_1_ showed only moderate antimicrobial activity against Gram-positive bacteria and negligible tyrosinase inhibitory activity. At this early stage of research with the novel isolate, we can only speculate on potential quorum sensing activity of stieleriacines. Analysis of potential target genes of a respective regulator part of a putative quorum sensing system is challenging given that 42% (2897 out of 6920) proteins encoded in the genome of strain Mal15^T^ are of unknown function. Experiments with the strain itself instead of isolated stieleriacine are particularly interesting, however, these require a Mal15^T^ mutant incapable of stieleriacine biosynthesis as negative control. Such a mutant can also provide important information on the stieleriacine biosynthesis pathway, however, due to lacking experience in terms of the genetic accessibility of strain Mal15^T^, this question needs to be addressed in follow-up studies.

Although some *N*-acyl amino acid compounds have been identified, the current knowledge on the underlying biosynthetic pathways is limited. Evidence for the presence of *N*-acylated derivatives of aromatic or basic amino acids was mainly based on a multitude of environmental DNA libraries. Characterisation of biosynthetic pathways for *N*-acyl amino acids is an important task in the future given that such compounds find potential biotechnological applications as antimicrobial surfactants and precursors for biodegradable polyesters in pharmaceutical and biomedical applications^30,31^. In case of the surfactants, the C_12_ chain as acyl residue (as present in stieleriacine A_1_) proved to have the optimum antibacterial activity among the single chain surfactants tested^31^. In that regard, genes of strain Mal15^T^ are interesting target for expression in heterologous microbial hosts.

A report on screening of soil metagenomic libraries revealed that genes coding for NAS proteins are frequently found adjacent to genes involved in the PEP-CTERM/exosortase protein sorting system (an exopolysaccharide-associated protein targeting system), indicating a functional link between both pathways^29^. Indeed, the same situation is also found in the genome of strain Mal15^T^. A PEP-CTERM motif protein-encoding gene (Mal15_37300) was found within the proposed stieleriacine biosynthetic gene cluster (Fig. 6), and many exopolysaccharide biosynthesis-related and secretion-related genes are encoded in the region upstream (Mal15_36520 to Mal17_37230, Supplementary Table 3). The conserved genetic organisation supports the notion of a functional link between stieleriacine biosynthesis, exopolysaccharide assembly and biofilm formation, which requires additional attention in future studies.

In conclusion, the identified stieleriacines stimulated growth initiation of Mal15^T^ cells and either promoted or inhibited biofilm formation of two different microorganisms, likely naturally competing with strain Mal15^T^ for biotic surfaces in marine environments. Although the two tested competitors might only be a small fraction of microorganisms dwelling in such environments, it allows first insights into strategies how slow-growing microorganisms compensate for lower growth rates and avoid being outcompeted. Stieleriacines are likely only one of several strategies how the survival of planctomycetes in aquatic environments is ensured and their high abundance on several marine biotic surfaces can be explained.

## Methods

### Sample collection and preparation

Seawater sediment samples from 30 cm depth were collected from Spain, Mallorca island, El Arenal, between Balneario 4 and 5 (39° 30′ 45.2″ N, 2° 44′ 49.1″ E) on September 23th, 2014. Samples were collected in sterile 50 mL polypropylene tubes, transferred to the laboratory, homogenised and processed.

### Culture and isolation conditions

For maintenance of strain Mal15^T^ M1 medium with HEPES (H) as buffering agent and additionally supplemented with *N*-acetyl glucosamine (NAG) and artificial seawater (ASW) (M1H NAG ASW medium) was used and prepared as described before^32^. Solid medium was prepared with 12 g/L (w/v) agar, washed three times with double distilled H_2_O and cooled to 55 °C prior to the addition of heat-sensitive solutions. For the initial strain isolation, solid M1H NAG ASW medium was supplemented with 100 µL of an ampicillin (50 mg/mL) and cycloheximide stock solution (20 mg/mL), dried for 30 min, inoculated with 100 µL homogenised sample material per plate and incubated at 20 °C in the dark until colony formation became visible. Single colonies were inoculated on fresh solid medium with the respective antibiotics. Axenic cultures were cryo-preserved in M1H NAG ASW medium supplemented with 50% glycerol or 5% DMSO and stored at −80 °C. Exponentially growing Mal15^T^ cells were used for analysis of carbon source utilisation (Supplementary Table 4) and enzymatic activities (Supplementary Table 5).

### Strain characterisation

Electron microscopy and basic light microscopy were performed as previously described^33,34^. For time-lapse microscopy, cells were observed using the CellASIC ONIX Microfluidic Platform (Merck Millipore). Physiological tests as well as phylogenetic and genome analysis were performed as previously described^35^. The results are shown in Supplementary Figs. 2–6.

### Statistics and reproducibility

All experiments were performed at least in biological triplicates. If not stated otherwise, data represent average and standard deviation. In all cases, detailed information is given in the respective figure captions. Significance of results is indicated by *p*-values given in the text.

### Phylogenetic analysis

16S rRNA gene sequence-based phylogeny was computed for strain Mal15^T^ (GenBank acc. no. MK554562), the type strains of all described planctomycetal species (assessed in May 2019) and all isolates recently published^10^ and described^1,36,37^. Phylogenetic inference based on 16S rRNA gene sequences and multi-locus sequence analysis (MLSA) was performed as described^36^. The 16S rRNA gene sequences were aligned with SINA^38^ and the phylogenetic inference was calculated with RAxML^39^ with a maximum likelihood approach with 1000 bootstraps, nucleotide substitution model GTR, gamma distributed rate variation and estimation of proportion of invariable sites (GTRGAMMAI option). Three 16S rRNA genes of bacterial strains from the PVC superphylum outside of the phylum Planctomycetes were used as outgroup. For MLSA the unique single-copy core genome of the analysed genomes (GenBank acc. no. for strain Mal15^T^: CP036264.1) was determined with proteinortho5^40^ with the ‘selfblast’ option enabled. The protein sequences of the resulting orthologous groups were aligned using MUSCLE v.3.8.31^41^. After clipping, partially aligned *C*-terminal and *N*-terminal regions and poorly aligned internal regions were filtered using Gblocks^42^. The final alignment was concatenated and clustered using the maximum likelihood method implemented by RAxML^39^ with the ‘rapid bootstrap’ method and 500 bootstrap replicates. Four planctomycetal genomes from different families in the class Planctomycetia were used as outgroup. The average nucleotide identity (ANI) was calculated using OrthoANI^43^. The average amino acid identity (AAI) was calculated using the aai.rb script of the enveomics collection^44^ and the percentage of conserved proteins (POCP) was calculated as described^45^. The *rpoB* nucleotide sequences were taken from publicly available planctomycetal genome annotations and the sequence identities were determined as described^46^. Alignment and matrix calculation were done with Clustal Omega^47^.

### Isolation and purification of stieleriacines from culture broth

For getting sufficient amounts of Mal15^T^ biomass, nutrient content of the culture broth was increased (1.0 g/L peptone, 1.0 g/L yeast extract, 40 mL/L 2.5% (w/v) glucose solution). Six hundred microlitre Mal15^T^ cultures were incubated for three days at 28 °C and 80 rpm in 2 L baffled flasks until 2% (w/v) of purified adsorbent resin XAD-16N (Rohm and Haas) were added. Cultures were further incubated for three days. In total, XAD-16N from 32 L culture was harvested and processed as previously described^32^. The combined crude extract was filtered with water/methanol (1:1) using a Strata-X 33 mm, Polymeric Reverse Phase Solid-Phase cartridge (Phenomenex). The solid phase was eluted with acetone and hexane yielding 308.25 mg crude product, which was fractionated using preparative RP-HPLC (PLC 2020, Gilson with Kromasil C_18_ column 250 × 20 mm, 7 µm; MZ-Analysetechnik) with deionised water and 0.1% formic acid (solvent A), and acetonitrile with 0.1% formic acid (solvent B). The following elution gradient was used: 40% B for 10 min, increased to 50% B in 3 min, and a gradient from 50–70% B in 60 min, thereafter 100% B for 10 min. Ultraviolet (UV) detection was carried out at 215 and 310 nm. Fractions were collected yielding five pure compounds. Stieleriacine A_**1**_ (**1**) (6.3 mg) was obtained at a retention time (*t*_R_) of 32.5 min, B_1_ (**2**) (2.0 mg) at *t*_R_ = 34.0 min, C (**3**) (0.8 mg) at *t*_R_ = 36.0 min, A_2_ (**4**) (4.9 mg) at *t*_R_ = 39.5 min, B_2_ (**5**) (3.5 mg) at *t*_R_ = 41.1 min (Fig. 1, Supplementary Fig. 7).

### Structure elucidation

HRESIMS mass spectra were measured with an Agilent 1200 series HPLC-UV system in combination with an ESI-TOF-MS (Maxis, Bruker) [column 2.1 × 50 mm, 1.7 µm, C_18_ Acquity UPLC BEH (Waters), solvent A: water with 0.1% formic acid, solvent B: acetonitrile with 0.1% formic acid, gradient: 5% B for 0.5 min increasing to 100% B in 19.5 min, maintaining 100% B for another 5 min, *R*_F_ = 0.6 mL/min, UV detection 200–600 nm. Nuclear magnetic resonance spectra were recorded on a Bruker Avance III 500 MHz spectrometer with a BBFO(plus) SmartProbe (^1^H 500 MHz, ^13^C 126 MHz), and a Bruker Avance III 700 MHz spectrometer with a 5 mm TCI cryoprobe (^1^H 700 MHz, ^13^C 175 MHz, ^15^N 71 MHz). UV spectra were recorded using a Shimadzu UV-VIS spectrophotometer UV-2450. Optical rotation was determined using a PerkinElmer 241 polarimeter. Correlations are shown in Supplementary Fig. 8.

### Antimicrobial activity assay

Determination of minimum inhibitory concentrations (MIC) was performed using serial dilution assays in 96-well microtiter plates. YMG medium (4 g/L yeast extract, 10 g/L malt extract, 4 g/L glucose, pH 7.2) was used for yeasts and filamentous fungi, while EBS medium (5 g/L casein peptone, 1 g/L meat extract, 1 g/L yeast extract, 5 g/L glucose, 50 mM HEPES, pH 7.0) was used for bacteria. Tested species were inoculated to initial cell densities of 10^5^–10^6^ colony-forming units per mL in the respective growth medium. MIC were assessed after 16 and 48 h of cultivation at 37 °C (bacteria) and 28 °C (yeasts and moulds), respectively. The MIC values represent the lowest concentration of stieleriacine at which no visible growth was observed.

### Cytotoxicity assay

In vitro cytotoxicity (IC_50_) was investigated using mouse fibroblast cell line L929 and HeLa KB3.1 cells as previously described^48^. Briefly, cells were cultivated in EBM-2 supplemented with 10% foetal bovine serum under 10% CO_2_ at 37 °C. Sixty microlitre of serial dilutions from an initial stock of 1 mg/mL stieleriacine in acetone was added to 120 μL aliquots of a cell suspension (50,000 cells per mL) in 96-well microtiter plates. After 5 days of incubation, a 3-(4,5-dimethylthiazol-2-yl)-2,5-diphenyltetrazolium bromide) (MTT) assay was performed. The absorbance was measured at 590 nm using an ELISA plate reader (Victor). The concentration, at which the growth of cells was inhibited to 50% of the control (IC_50_), was obtained from the dose−response curves. Acetone served as negative control.

### Tyrosinase inhibition assay

The tyrosinase inhibition assay was performed as previously described^18^ with the following modifications: 80 µL of compound, dissolved in PBS (0.1 M, pH 6.8) with 10% methanol at various concentrations, were added to 80 µL of PBS. PBS with 10% methanol served as a negative control. Eighty microlitre of mushroom-tyrosinase (100 units per mL) and 40 µL of l-tyrosine (2.5 mM) were added to each well. Phosphate buffer and tyrosinase served as a negative control. Reactions were incubated at 37 °C for 45 min and the absorbance was measured at 490 nm using the Infinite 200 Pro multimode reader (Tecan). The percentage of tyrosinase inhibition was calculated as follows: [(Δ*A*_control_ – Δ*A*_sample_)/Δ*A*_control_] × 100.

### Time-lapse microscopy

To analyse the physiological effect of stieleriacine A_1_ with time-lapse microscopy, a preculture of Mal15^T^ cells was inoculated 1:10 in fresh M1H NAG ASW medium and incubated for 72 h (28 °C, 80 rpm). Before loading, the CellASIC® microfluidic chamber was washed with a constant flow of medium (2 × 5 psi, 5 min). Stieleriacine A_1_ was provided dissolved in 132 µL acetone at 1.34 µM, while medium supplemented with the same volume of acetone was used as negative control. Cells were monitored for up to 36 h at 28 °C at 2 × 1 psi flow rate. Phase contrast images were acquired and processed as previously described^49^. In total, 1264 cells were analysed and all experiments were performed in three biological replicates.

### Biofilm formation assay

Biofilm formation of *P. inhibens* and *S. dubius* was analysed by a crystal violet assay^3,50^. *P. inhibens* and *S. dubius* were grown in 10 mL MB medium (MB, Carl Roth) at 28 °C with vigorous shaking to the early exponential phase (OD_600_ of 0.4). Cultures were subsequently diluted to an OD_600_ of 0.065. 100 μL of the diluted culture was transferred to a respective well of a sterile polystyrene 96-well assay plate (Corning, New York, NY, USA; Costar 3370). 1.34 µM or 134 µM of stieleriacine A_1_ solved in acetone or pure acetone were added to the wells prior to statically culturing of the plates for 24 h. After incubation, medium along with planktonic cells was removed and wells were washed twice with H_2_O. Two hundred microlitre of 0.5% crystal violet (CV) was added to the wells, which were incubated at room temperature for 10 min. After staining, the CV solution was removed and each well was washed twice with H_2_O to remove the residual dye and dried overnight. For scoring of cell attachment, the CV was extracted from the biofilm with 200 μL 95% (v/v) ethanol, of which 100 μL were transferred to a new 96-well plate before the absorbance was determined at 595 nm using the Tecan microplate reader (Infinite® 200 PRO). For each experiment, four biological replicates and two technical replicates were performed.

### Analysis of the stieleriacine production rate

To analyse the production rate of stieleriacine A_1_, precultures of strain Mal15^T^ were inoculated 1:10 in fresh M1H NAG ASW medium in triplicates and incubated for 7 days (152 h, 28 °C, 80 rpm). For the next five days 30 mL of each culture were harvested every 12 h, followed by 2 days harvesting after every 16 h. The harvested material was analysed for optical density (OD_600_), biomass and stieleriacine A_1_ concentration.

### Reporting summary

Further information on research design is available in the Nature Research Reporting Summary linked to this article.

## Supplementary information

Supplementary Information

Supplementary Movie 1

Supplementary Movie 2

Description of Additional Supplementary Files

Reporting Summary

## Data Availability

The genome and 16S rRNA gene sequence of strain Mal15^T^ are available from GenBank under accession numbers CP036264.1 and MK554562.1, respectively. Other relevant data are available from the corresponding authors upon request. The putative stieleriacine biosynthetic gene cluster was deposited at the MiBiG (Minimum Information about a Biosynthetic Gene cluster) database and can be found under accession number BGC0002080.
