# Author Correction: The planctomycete *Stieleria maiorica* Mal15^T^ employs stieleriacines to alter the species composition in marine biofilms

**DOI:** 10.1038/s42003-020-01228-1

**Published:** 2020-08-31

**Authors:** Nicolai Kallscheuer, Olga Jeske, Birthe Sandargo, Christian Boedeker, Sandra Wiegand, Pascal Bartling, Mareike Jogler, Manfred Rohde, Jörn Petersen, Marnix H. Medema, Frank Surup, Christian Jogler

**Affiliations:** 1 Department of Microbiology, Radboud University, Nijmegen, The Netherlands; 2 Leibniz Institute DSMZ, Braunschweig, Germany; 3 Helmholtz Centre for Infection Research, Braunschweig, Germany; 4 German Centre for Infection Research (DZIF), Braunschweig, Germany; 5 Institute for Biological Interfaces 5, Karlsruhe Institute of Technology, Eggenstein-Leopoldshafen, Germany; 6 Central Facility for Microscopy, Helmholtz Centre for Infection Research, Braunschweig, Germany; 7 Bioinformatics Group, Wageningen University, Wageningen, The Netherlands; 8 Department of Microbial Interactions, Friedrich-Schiller University, Jena, Germany

**Keywords:** Biofilms, Small molecules, Water microbiology

Correction to: *Communications Biology* 10.1038/s42003-020-0993-2, published online 12 June 2020.

The original published version of the article did not contain the novel genus and species descriptions within the article, but rather only in the Supplementary Notes. The descriptions have been added to the “Results” section in the HTML and PDF versions of the article.

