# Author Correction: The planctomycete *Stieleria maiorica* Mal15^T^ employs stieleriacines to alter the species composition in marine biofilms

**DOI:** 10.1038/s42003-020-01271-y

**Published:** 2020-09-21

**Authors:** Nicolai Kallscheuer, Olga Jeske, Birthe Sandargo, Christian Boedeker, Sandra Wiegand, Pascal Bartling, Mareike Jogler, Manfred Rohde, Jörn Petersen, Marnix H. Medema, Frank Surup, Christian Jogler

**Affiliations:** 1 Department of Microbiology, Radboud University, Nijmegen, The Netherlands; 2 Leibniz Institute DSMZ, Braunschweig, Germany; 3 Helmholtz Centre for Infection Research, Braunschweig, Germany; 4 German Centre for Infection Research (DZIF), Braunschweig, Germany; 5 Institute for Biological Interfaces 5, Karlsruhe Institute of Technology, Eggenstein-Leopoldshafen, Germany; 6 Central Facility for Microscopy, Helmholtz Centre for Infection Research, Braunschweig, Germany; 7 Bioinformatics Group, Wageningen University, Wageningen, The Netherlands; 8 Department of Microbial Interactions, Friedrich-Schiller University, Jena, Germany

**Keywords:** Biofilms, Small molecules, Water microbiology

Correction to: *Communications Biology* 10.1038/s42003-020-0993-2, published online 12 June 2020.

In the original version of the Article “The planctomycete *Stieleria maiorica* Mal15^T^ employs stieleriacines to alter the species composition in marine biofilms”, the authors described the new genus *Stieleria* and its type species *Stieleria maiorica*. However, the descriptions were not presented in the paper in the format required by Rule 27 and Rule 30 of the International Code of Nomenclature of Prokaryotes. Therefore, we here present the descriptions in the correct format.

**Description of** ***Stieleria*** **gen. nov**.

*Stieleria* (Stie.le’ri.a. N.L. fem. n. *Stieleria* named in honor of Anja Heuer, née Stieler, an extraordinary skilled German technician at the Leibniz Institute DSMZ, who played a key role in the cultivation of literally hundreds of novel planctomycetal strains). The round-to-pear-shaped cells with a smooth cell surface form rosettes or short chains. Cells reproduce by polar budding. In liquid culture, they produce an extracellular matrix that interconnects cells in aggregates. Daughter cells are motile, while mother cells are non-motile. The lifestyle is heterotrophic, obligatory aerobic, and mesophilic. The genus belongs to the phylum *Planctomycetes*, class *Planctomycetia*, order *Pirellulales*, and family *Pirellulaceae*. The type species is *Stieleria maiorica*.

**Description of** ***Stieleria maiorica*** **sp. nov**.

*Stieleria maiorica* (ma.io’ri.ca. N.L. fem. adj. *maiorica*, pertaining to the island Mallorca, Spain, on which the type strain was isolated). In addition to the features described above, the species exhibits the following properties. Colonies are pink-colored on solid medium. Cells are 1.9 ± 0.2 × 1.4 ± 0.2 µm in size. Motile daughter cells originate through budding from sessile mother cells. Gram staining delivers no clear results. The oxidase assay was negative, while the catalase assay was positive. The organism can degrade a wide range of carbon sources. In particular, strong signals were observed for *N*-acetyl-d-galactosamine, *N*-acetyl-d-glucosamine, l-arabinose, d-cellobiose, l-fucose, d-fructose, d-galactose, gentiobiose, α-d-glucose, d-gluconic acid, glucuronamide, d-glucuronic acid, α-d-lactose, lactulose, d-mannose, d-melibiose, β-methyl-d-glucoside, d-raffinose, l-rhamnose, sucrose, d-trehalose, turanose, and d-psicose, while for the carbon sources acetic acid, dextrin, d-galactonic acid lactone, d-glucose-6-phosphate, α-ketoglutaric acid, maltose, and d-mannitol only weak signals were detected. The enzyme repertoire includes alkaline phosphatase, esterase (C4), esterase lipase (C8), leucine arylamidase, valine arylamidase, acid phosphatase, and naphthol-AS-BI-phosphohydrolase. Growth of the type strain occurs between pH 5.5 and 9.0 with an optimum at pH 7.5. The optimal growth temperature of the type strain was determined to be 35 °C, but cells could grow in a range from 11 °C up to at least 37 °C. The complete chromosome of the type strain comprises 9,894,293 bp, with a G + C content of 59.3%. The type strain is Mal15^T^ (DSM 100215^T^ = LMG 29790^T^, synonym Malle15), which was isolated from seawater sediment on Mallorca island, Spain.

