# Supplementary material for: The planctomycete Stieleria maiorica Mal15T employs stieleriacines to alter the species composition in marine biofilms
Source: Commun Biol. 2020 Jun 12;3:303. doi: 10.1038/s42003-020-0993-2 (PMC7293339; doi:10.1038/s42003-020-0993-2)
Supplement: Supplementary file 1 — Supplementary Information [file 42003_2020_993_MOESM1_ESM.pdf]

Supplementary Figures

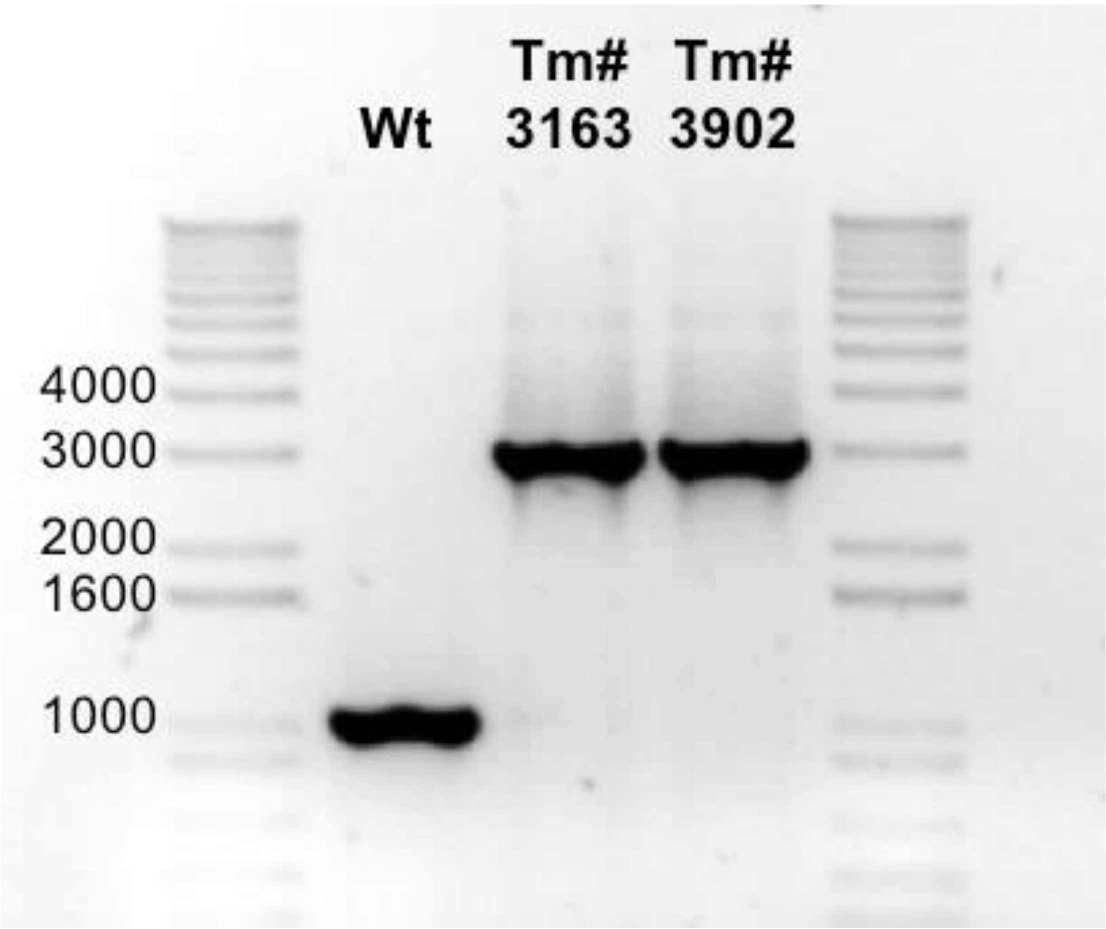

Supplementary Fig. 1. Agarose gel electrophoresis after PCR for verification of correct insertion of the EZ-Tn5 transposon in the *luxR* gene. The PCR fragments of wild-type DNA (Wt) have a size of about 1000 bp. The PCR products from the transposon mutants Tm#3163 and Tm#3902 have a size of about 3000 bp, thus showing the expected insertion of the EZ-Tn5 transposon with a size of 2001 bp.

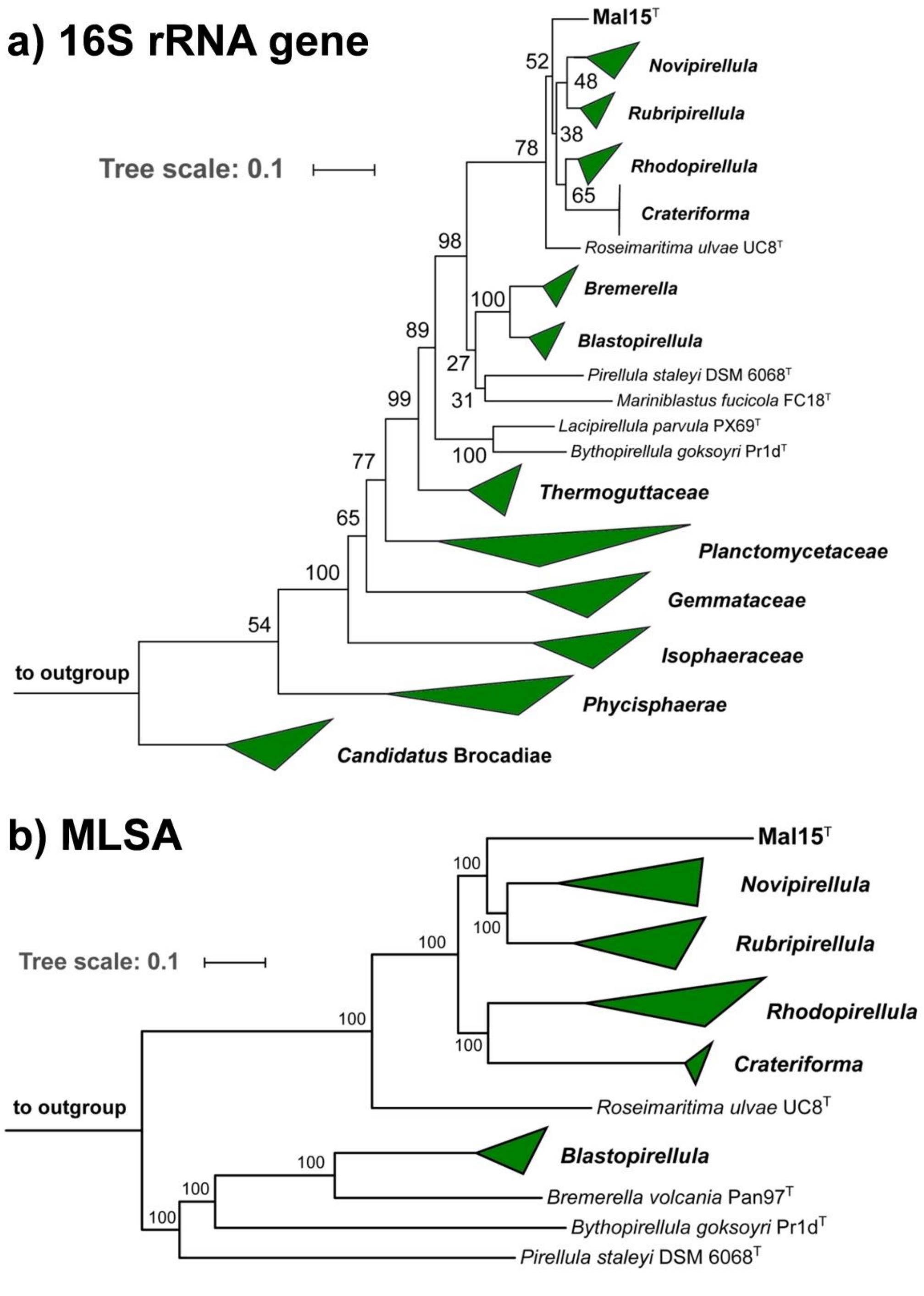

Supplementary Fig. 2. Maximum likelihood 16S rRNA gene sequence- and MLSA-based phylogeny. The phylogenetic trees show the position of strain Mal15^{T} in relation to its closest described relatives. 16S rRNA gene sequence- and MLSA-based phylogeny were computed using the maximum likelihood method. Bootstrap values after 1,000 re-samplings (16S rRNA gene) / 500 re-samplings (MLSA) are given at the nodes (in %). The outgroup for the 16S rRNA gene sequence-based tree consists of three 16S rRNA genes from the PVC superphylum outside of the phylum *Planctomycetes*. In the MLSA-based tree, three species of the family *Planctomycetaceae* served as outgroup.

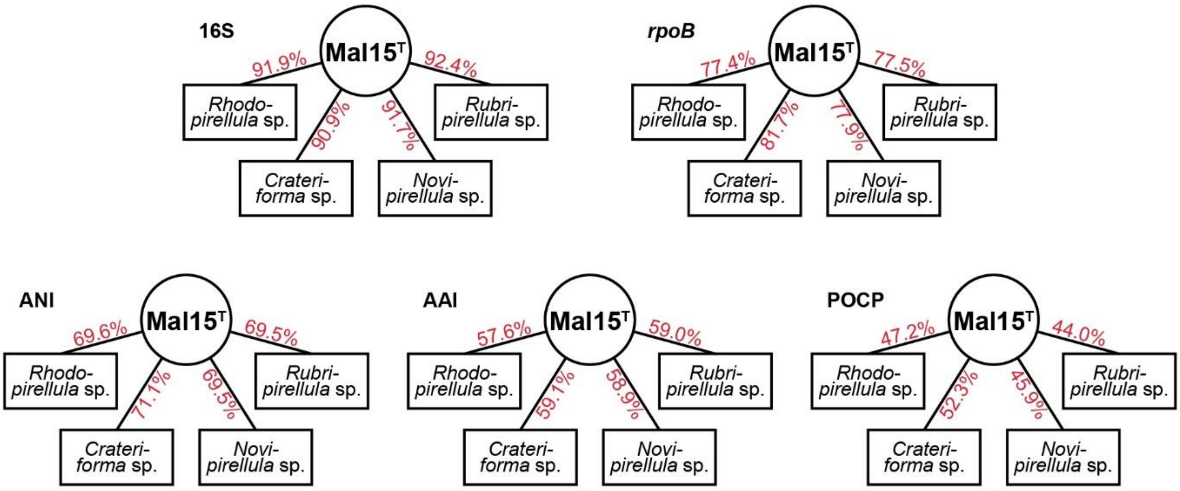

Supplementary Fig. 3. Phylogenetic marker values of isolate Mal15^T and its closest neighbours. The numbers give the minimal values shared between strain Mal15^T and any described member of the respective genera for 16S rRNA gene sequence identity (16S), *rpoB* nucleotide sequences identity (*rpoB*), average nucleotide identity (ANI), average amino acid identity (AAI) and percentage of conserved proteins (POCP).

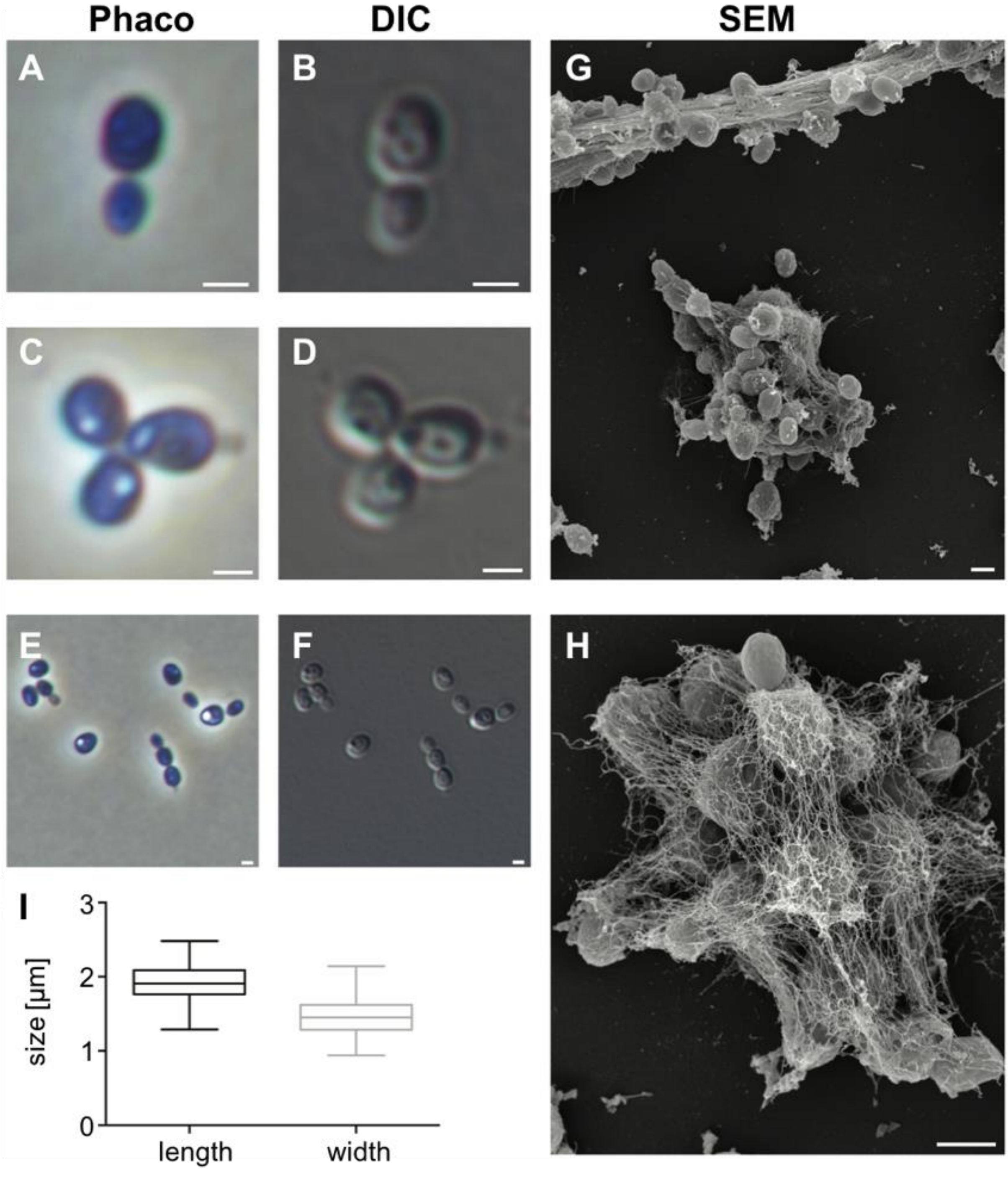

Supplementary Fig. 4. Morphology of strain Mal15^{T}. A-F Phase contrast (Phaco) and differential interference contrast (DIC) microscopy of Mal15^{T} cells. Cells are round to pear-shaped and sometimes connected to rosettes or chains with their narrow poles. **g-h** Scanning electron microscopy (SEM) revealed the existence of an extracellular matrix which interconnects cells in aggregates. **I** Cell size ranges between 1.9 ± 0.2 x 1.4 ± 0.2 µm (n=100 cells). Scale bar, 1 µm.

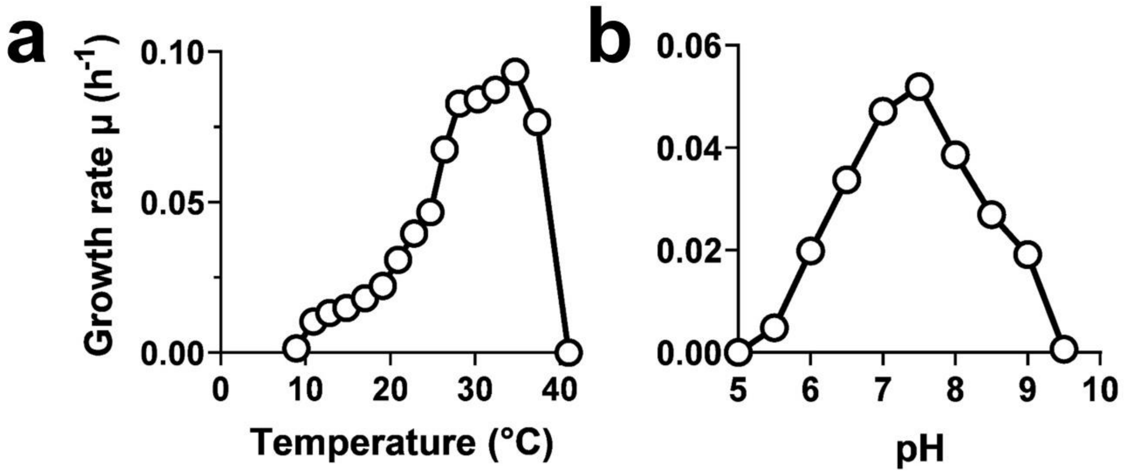

Supplementary Fig. 5. Temperature and pH optimum of strain Mal15^{T}. The strain was incubated at temperatures from 8.9 °C to 37.3 °C (a) and at a pH range of 5.0 to 9.5 (b) in M1H NAG ASW. Maximal growth rates were calculated from average OD_{600} values obtained from triplicate cultivations. Optimal growth was observed at 35 °C and pH 7.5.

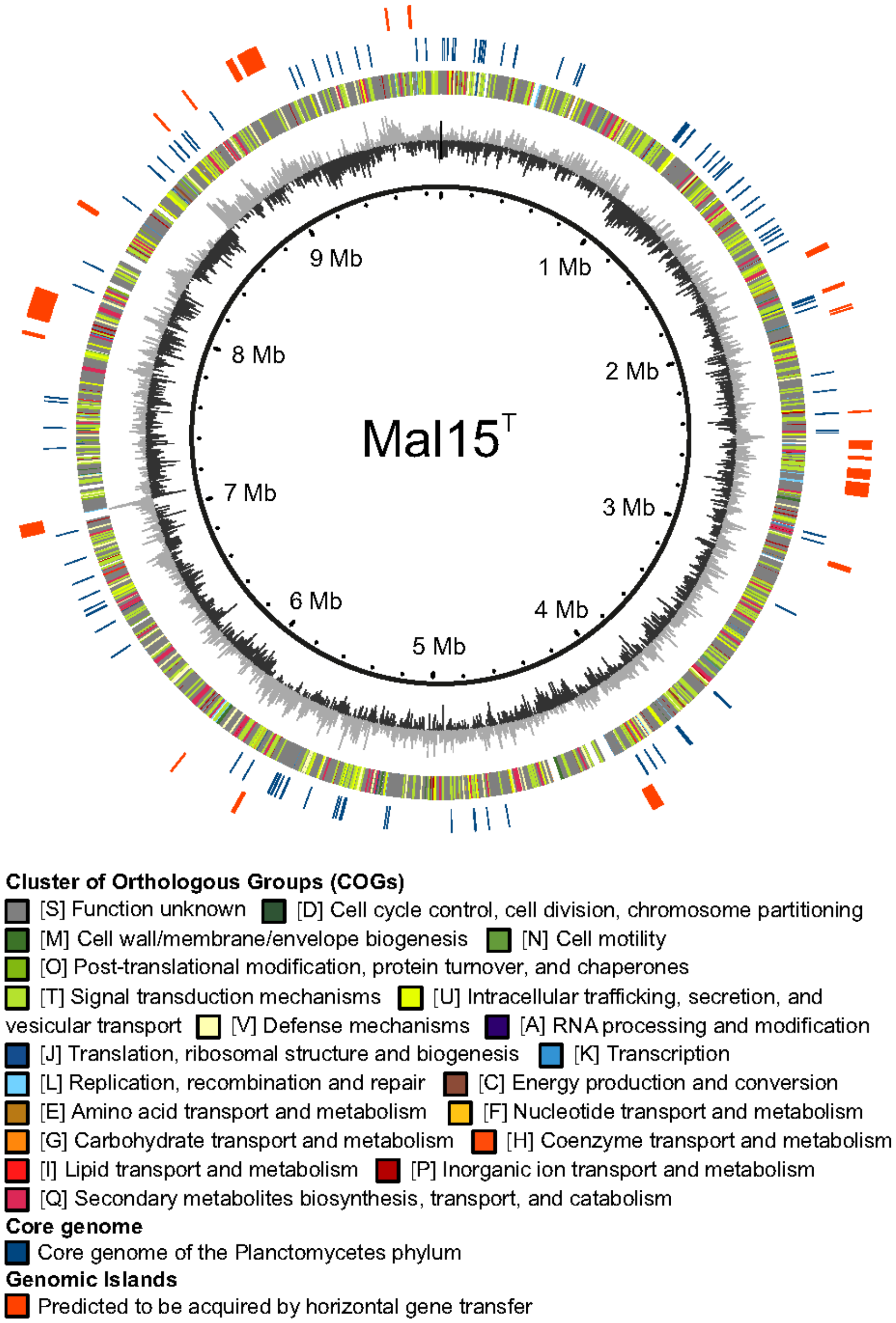

Supplementary Fig. 6. Circular genome plots of strain Mal15^{T}. The genome of strain Mal15^{T} is visualised by five circles. The innermost circle is labeled with the strain name and represents the circularised genome; the plotted ticks indicate the genome size (0.2 Mb per tick). The second circle depicts the GC skew in light and dark grey. The middle circle shows the color-coded COG (Cluster of Orthologous Groups) categories of the encoded proteins. The fourth circle illustrates the core genome, which was determined by the reciprocal best alignment method on three different phylogenetic levels as indicated by color. Finally, predicted genomic islands are indicated on the outermost circle.

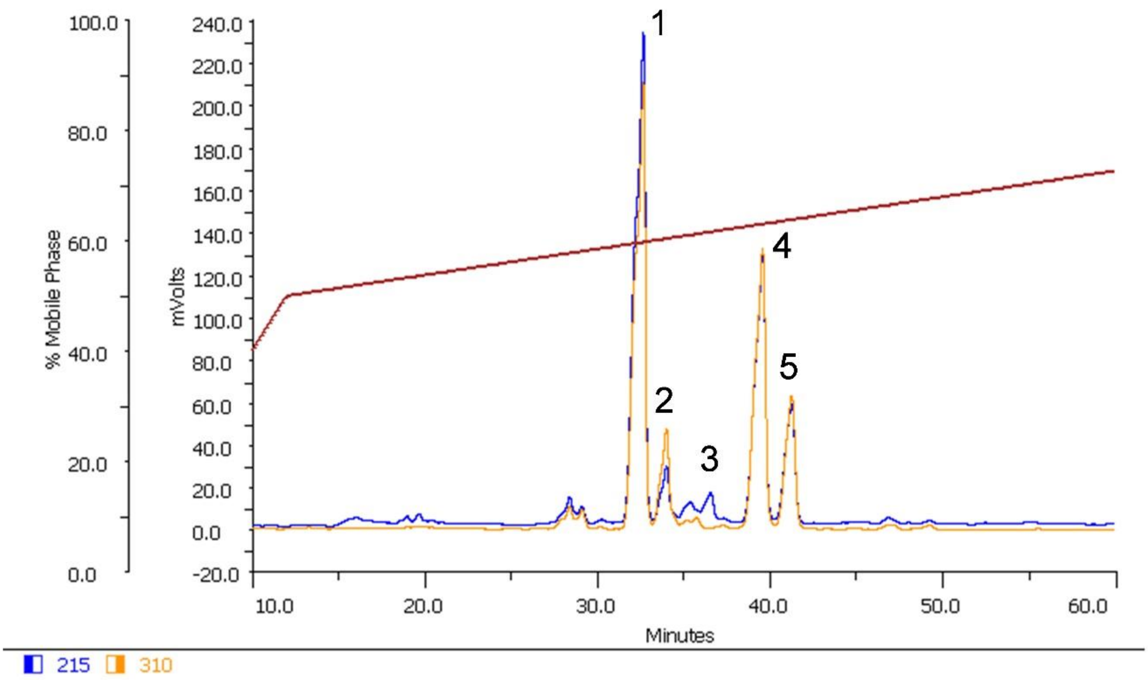

Supplementary Fig. 7. UV-*vis* fractionation chromatogram for crude extract of strain Mal15^T. The crude extract was fractionated using preparative RP-HPLC. A Kromasil C_{18} column served as stationary phase. Deionised water with 0.1% formic acid as solvent A, and acetonitrile with 0.1% formic acid as solvent B, were used as mobile phase. The used elution gradient (brown curve) was 40% B for 10 min, increased to 50% B in 3 min, and a gradient from 50–70% B in 60 min, thereafter 100% B for 10 min. UV detection was carried out at 215 nm (blue curve) and 310 nm (orange curve). Stieleriacine A_1 (**1**) was obtained at a retention time (t_R) = 32.5 min, B_1 (**2**) at t_R = 34.0 min, C (**3**) at t_R = 36.0 min, A_2 (**4**) at t_R = 39.5 min, B_2 (**5**) at t_R = 41.1 min.

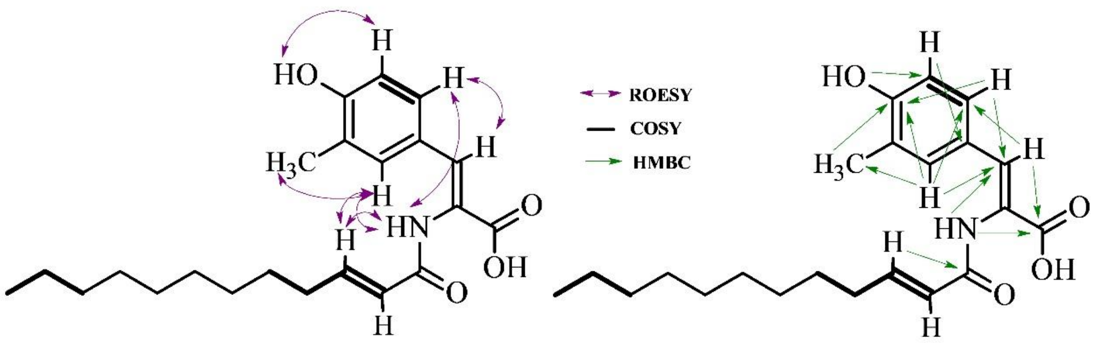

Supplementary Fig. 8. Key COSY, HMBC, and ROESY correlations for stieleriacine A_1 and A_2.

Supplementary Fig. 9. HRESIMS data of stieleriacine A_1 (1)

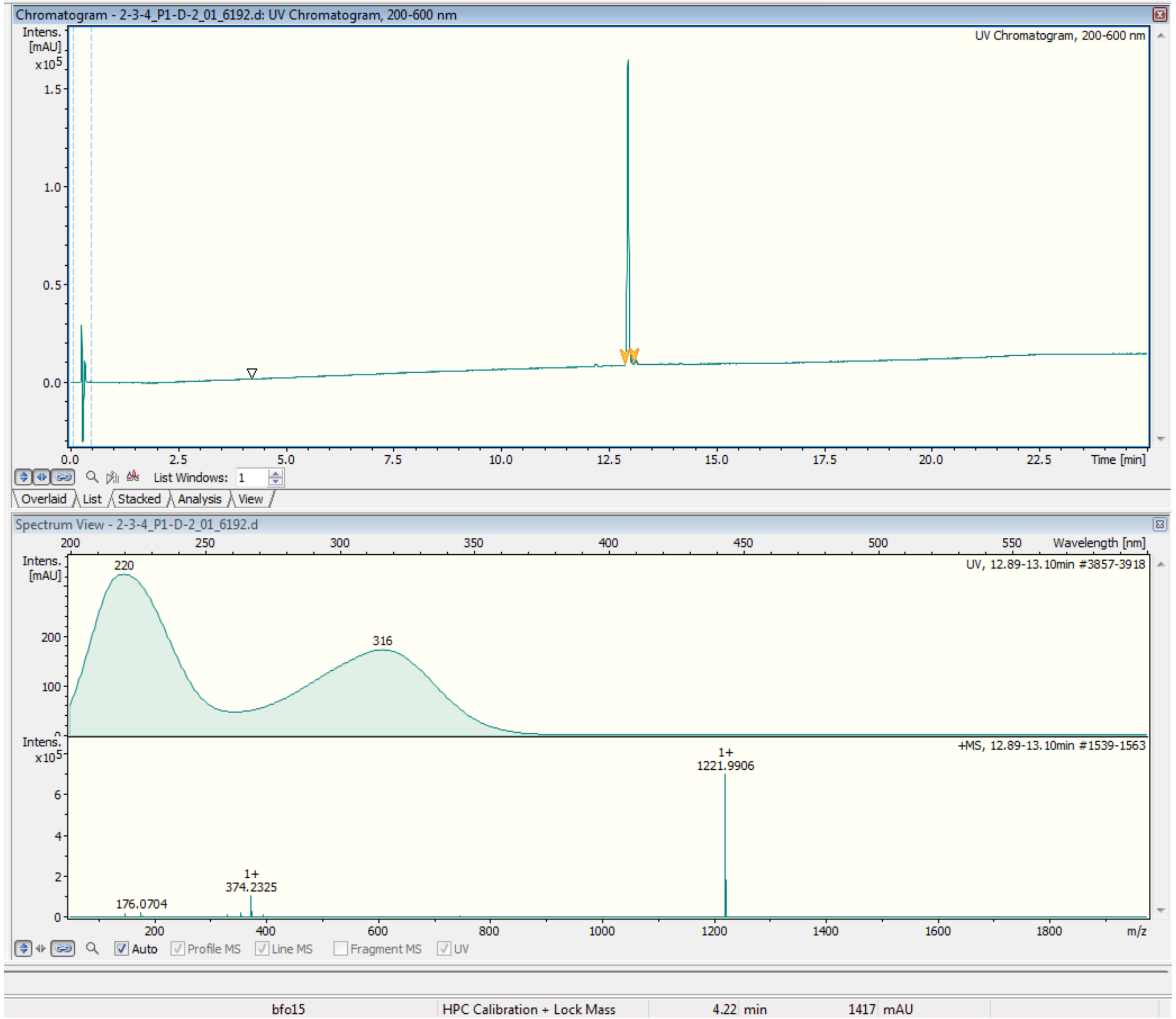

Supplementary Fig. 10. ^{1}H NMR spectrum (700 MHz, DMSO-d_6) of stieleriacine A_1 (1)

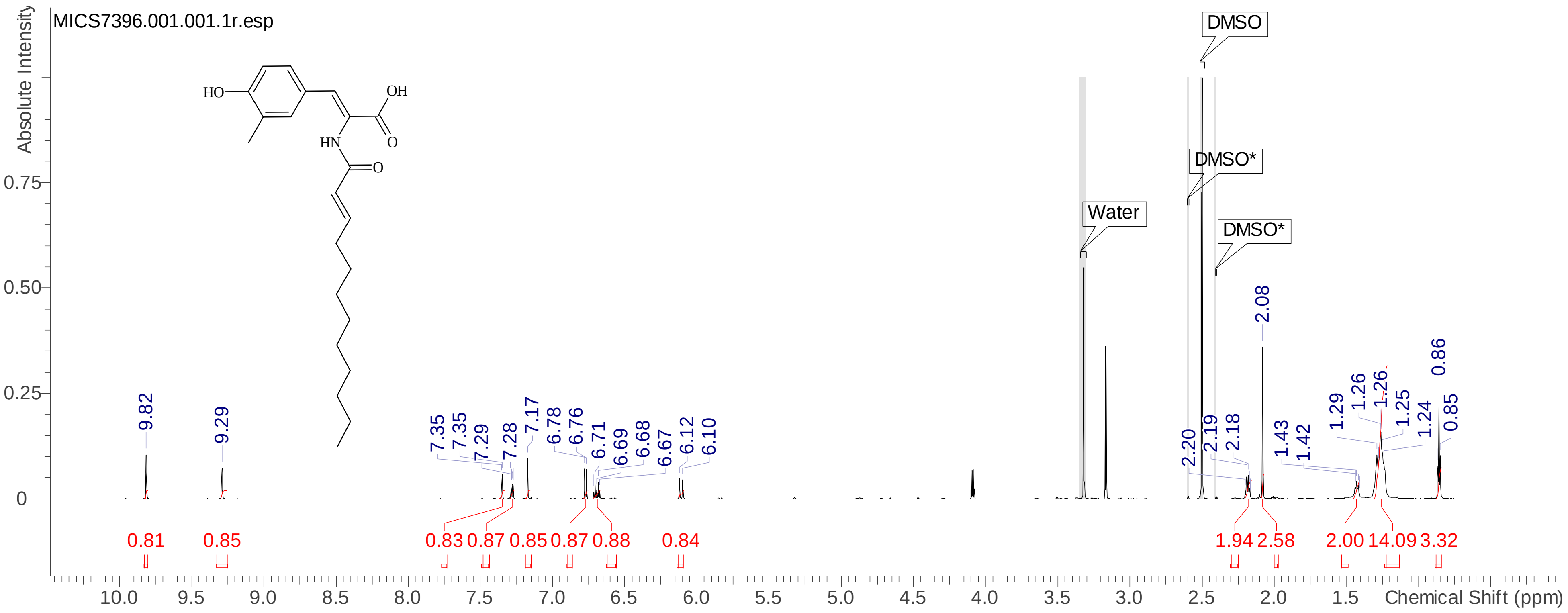

Supplementary Fig. 11. ^{13}C NMR spectrum (176 MHz, DMSO-d_6) of stieleriacine A_1 (1)

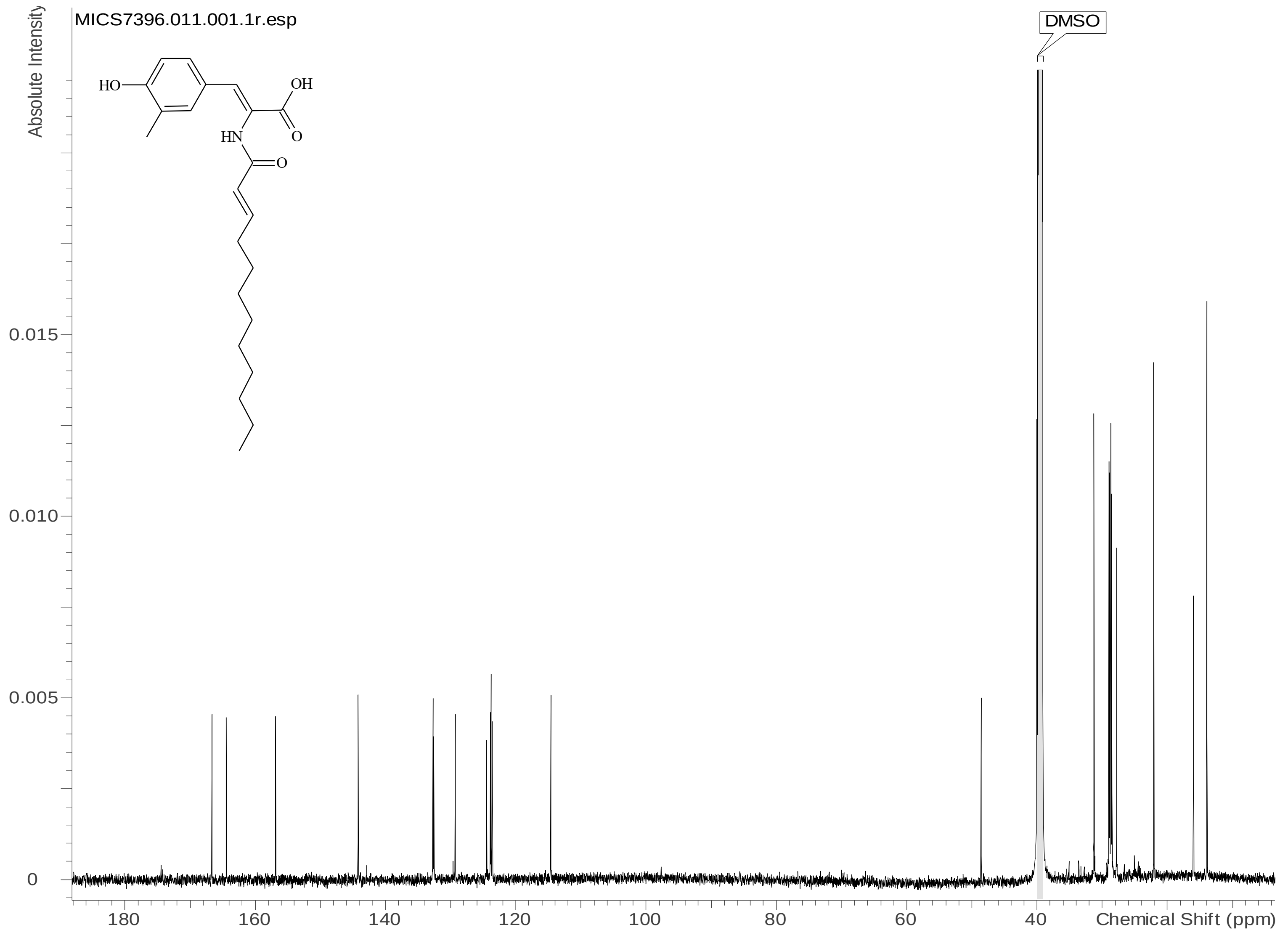

Supplementary Fig. 12. COSY NMR spectrum (700 MHz, DMSO-d_6) of stieleriacine A_1 (1)

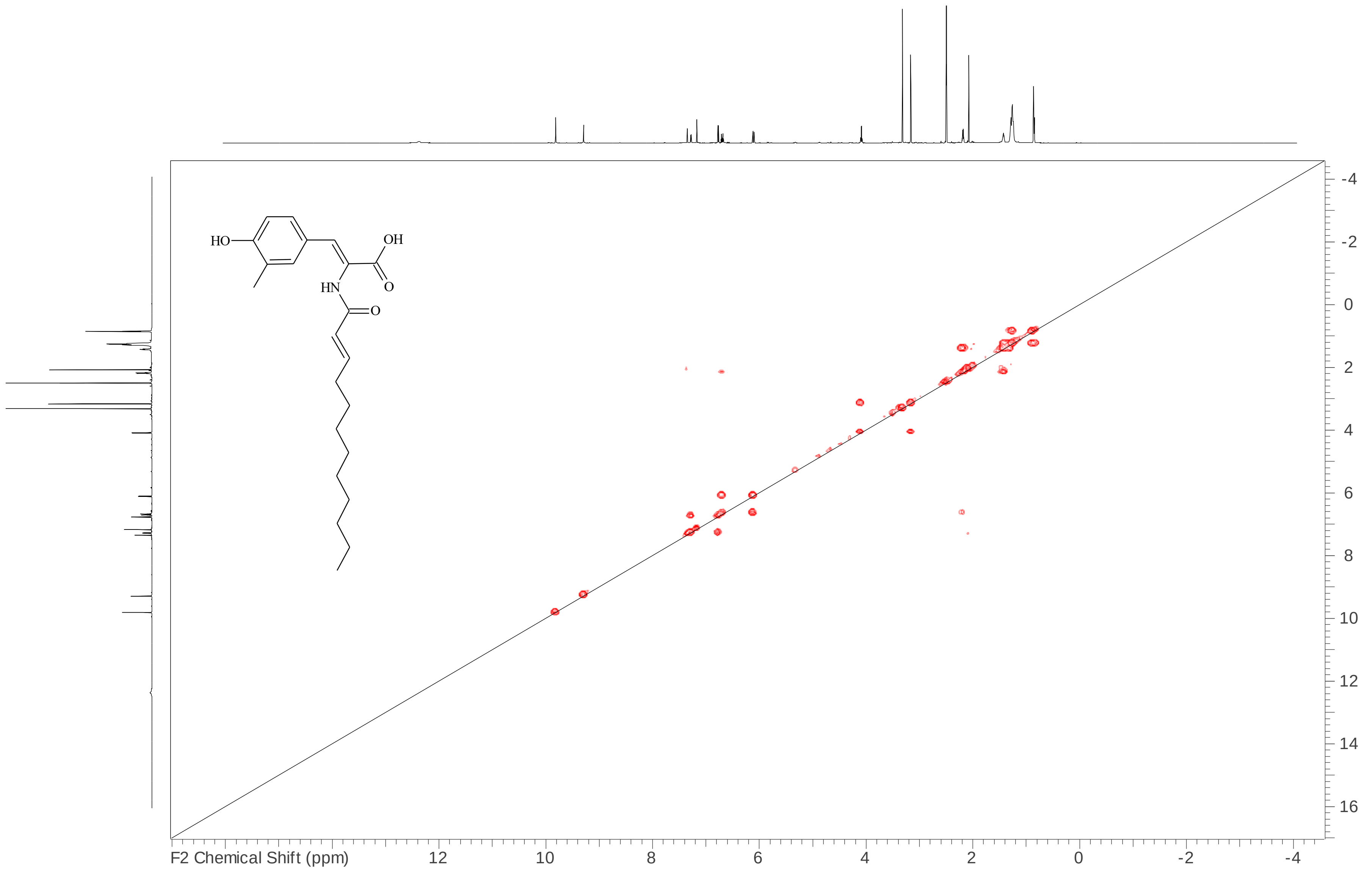

Supplementary Fig. 13. TOCSY NMR spectrum 700 MHz, DMSO-d_6) of stieleriacine A_1 (1)

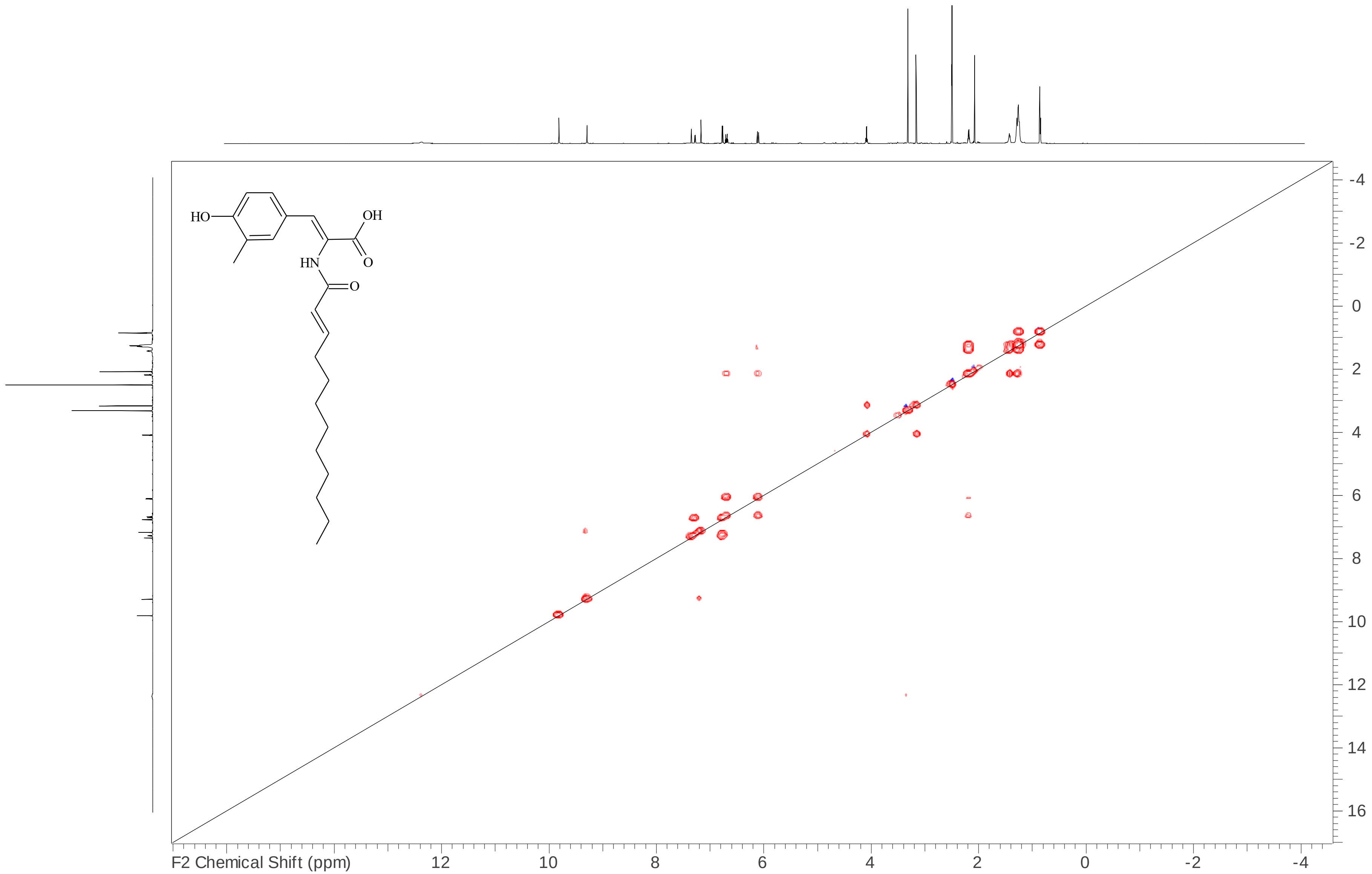

Supplementary Fig. 14. HSQC NMR spectrum (700 MHz, DMSO-d_6) of stieleriacine A_1 (1)

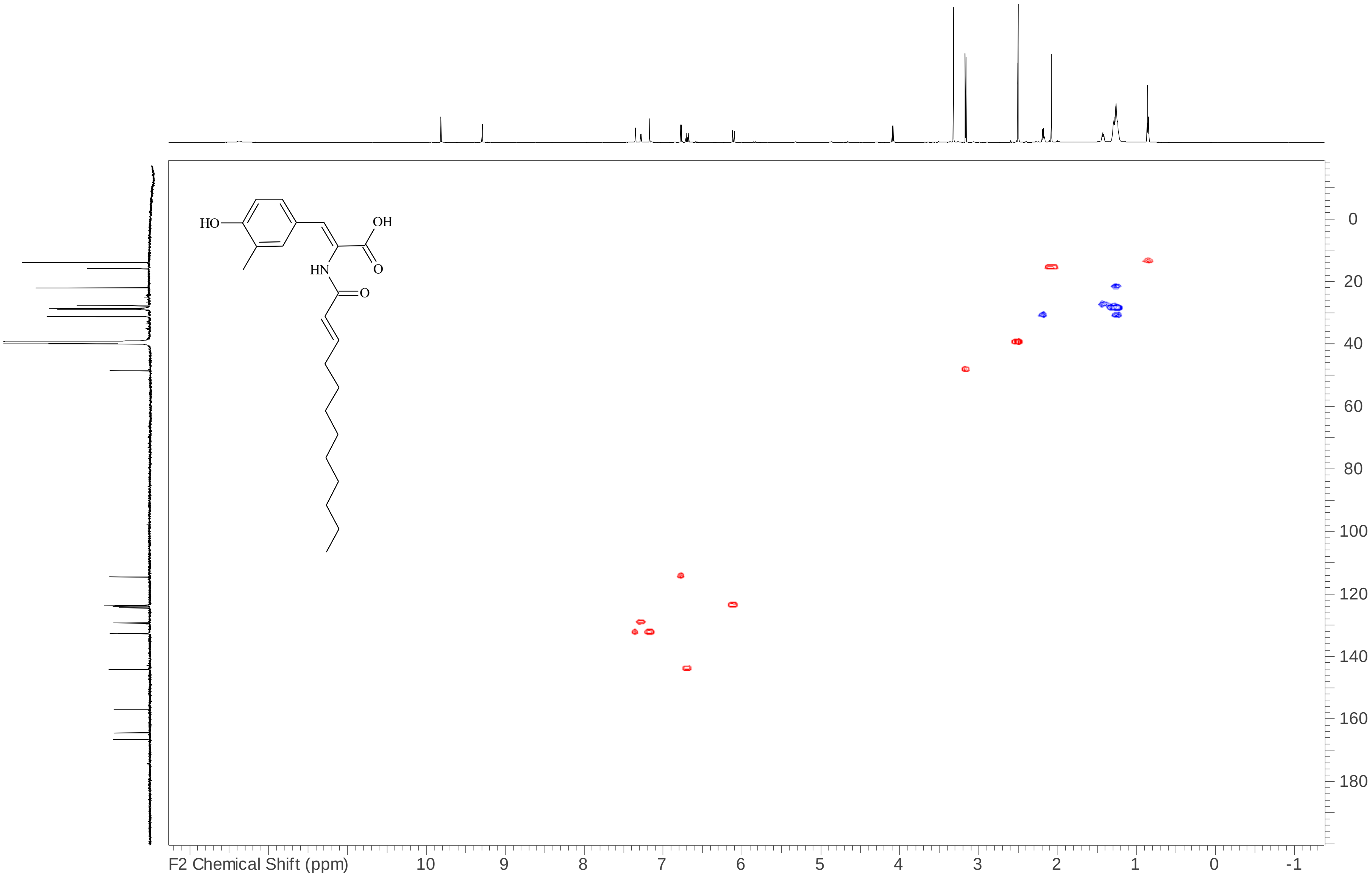

Supplementary Fig. 15. HMBC NMR spectrum (700 MHz, DMSO-d_6) of stieleriacine A_1 (1)

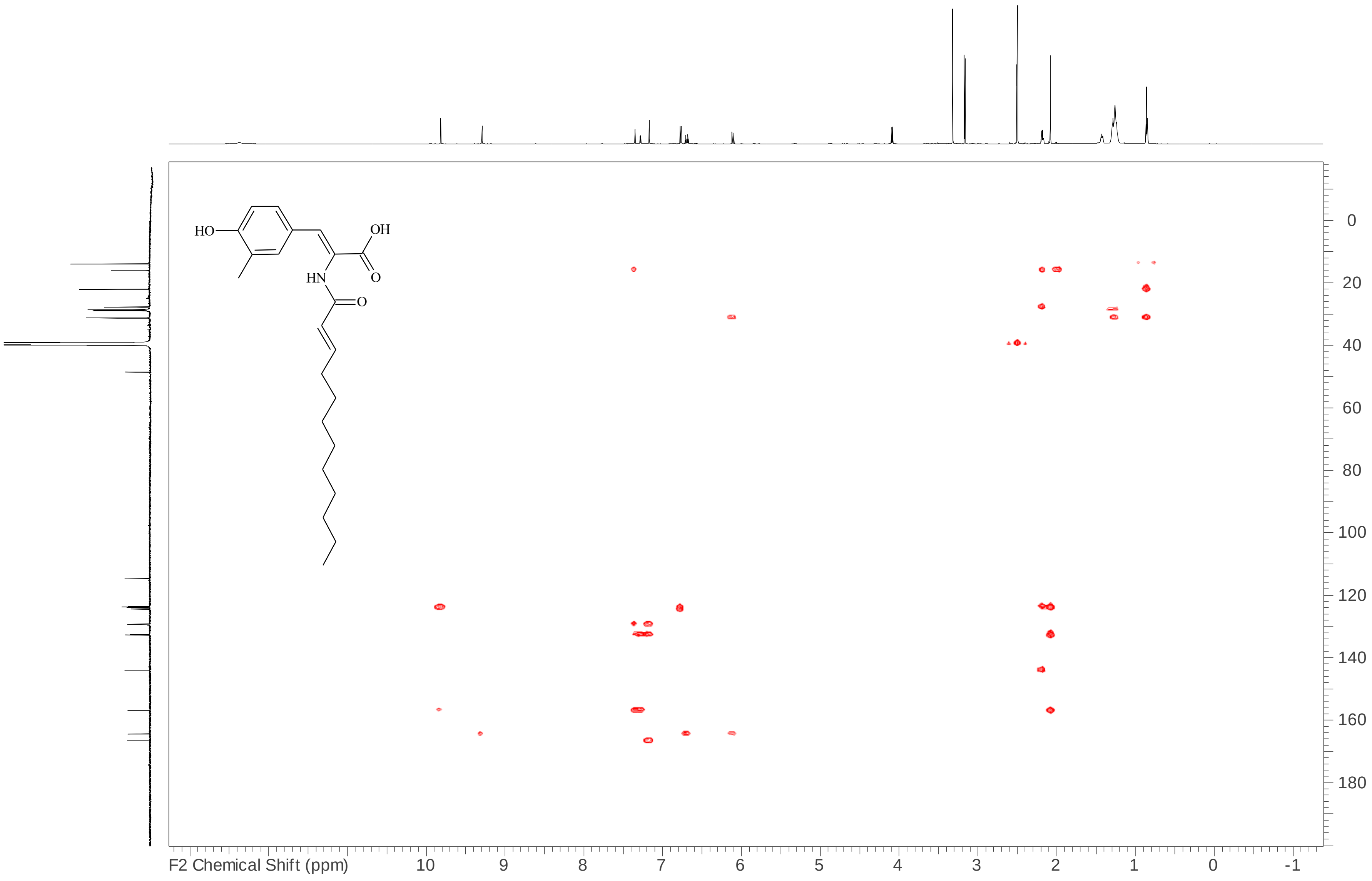

Supplementary Fig. 16. ROESY NMR spectrum (700 MHz, DMSO-d_6) of stieleriacine A_1 (1)

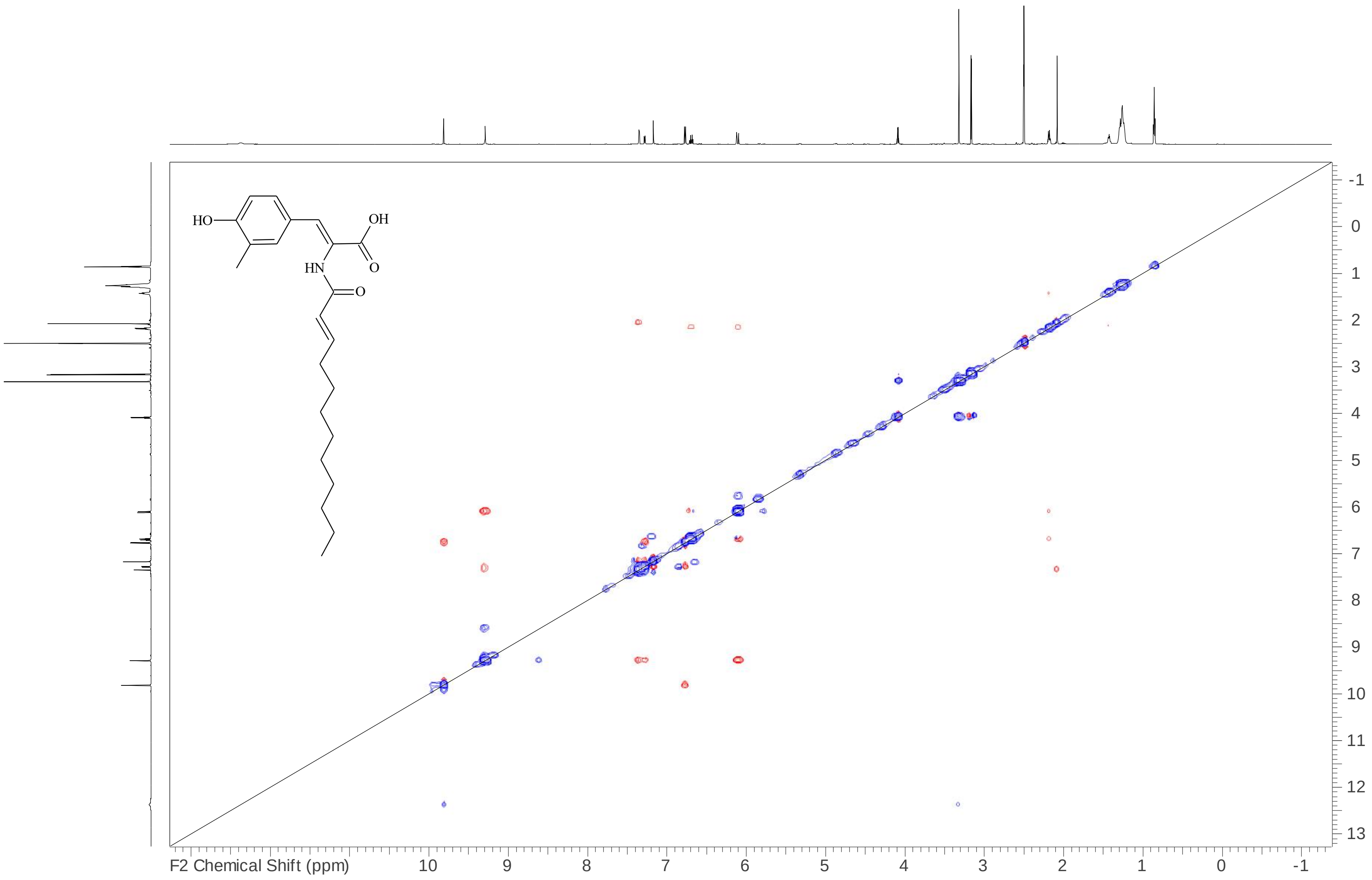

Supplementary Fig. 17. HRESIMS of stieleriacine B_1 (2)

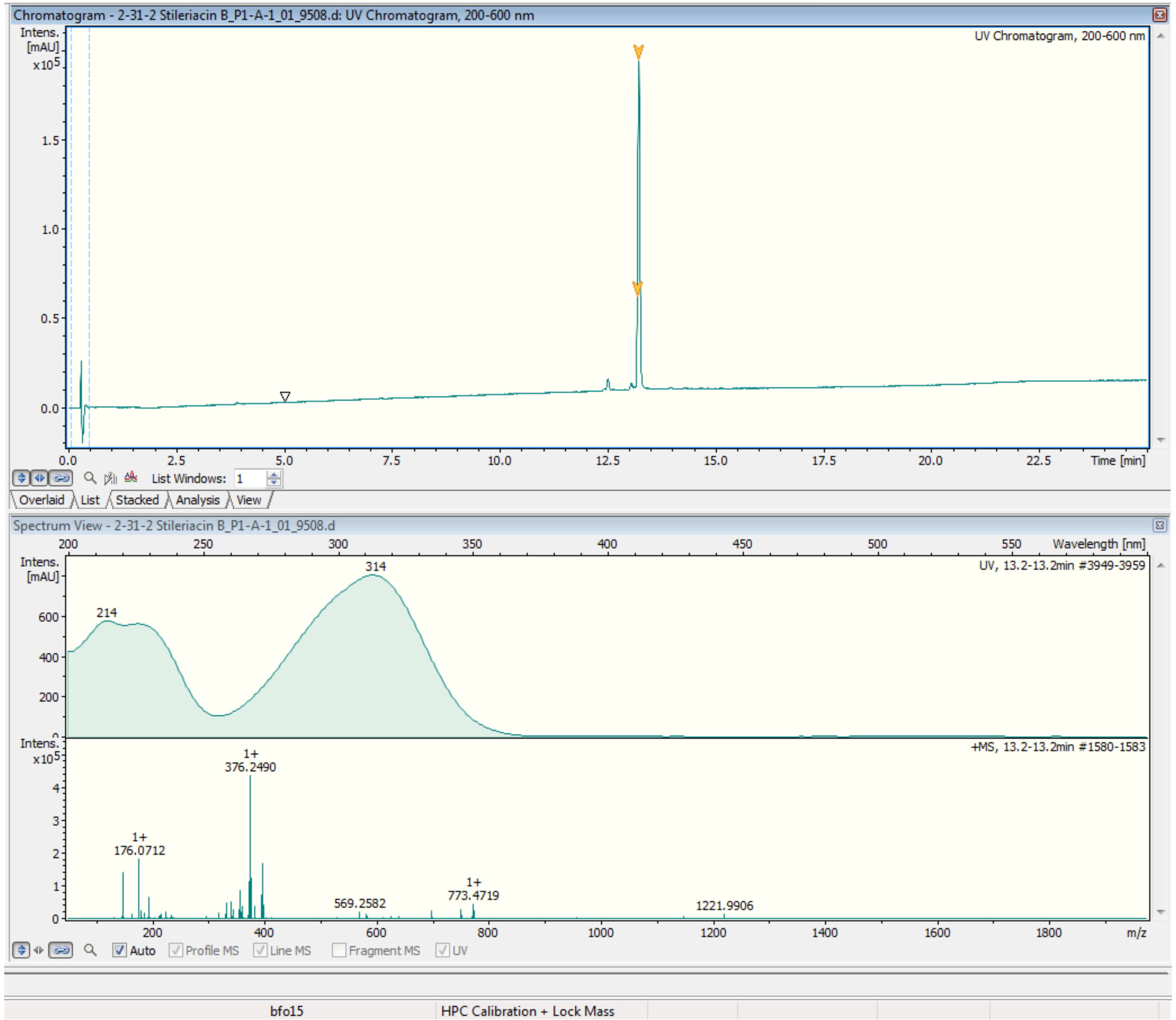

Supplementary Fig. 18. ^{1}H NMR spectrum (500 MHz, DMSO-d_6) of stieleriacine B_1 (2)

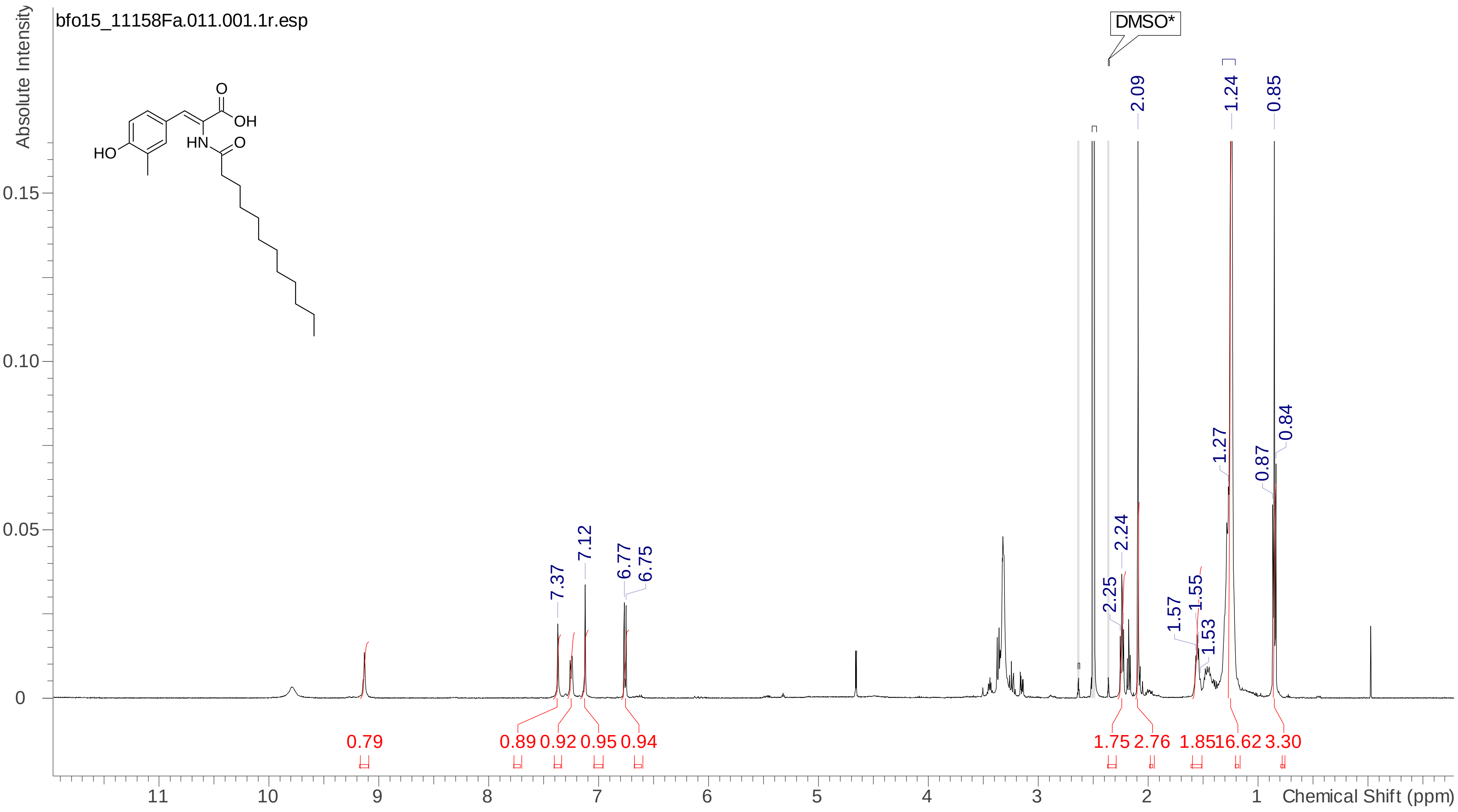

Supplementary Fig. 19. ^{13}C **NMR spectrum (126 MHz, DMSO-d_6) of stieleriacine B_1 (2)**

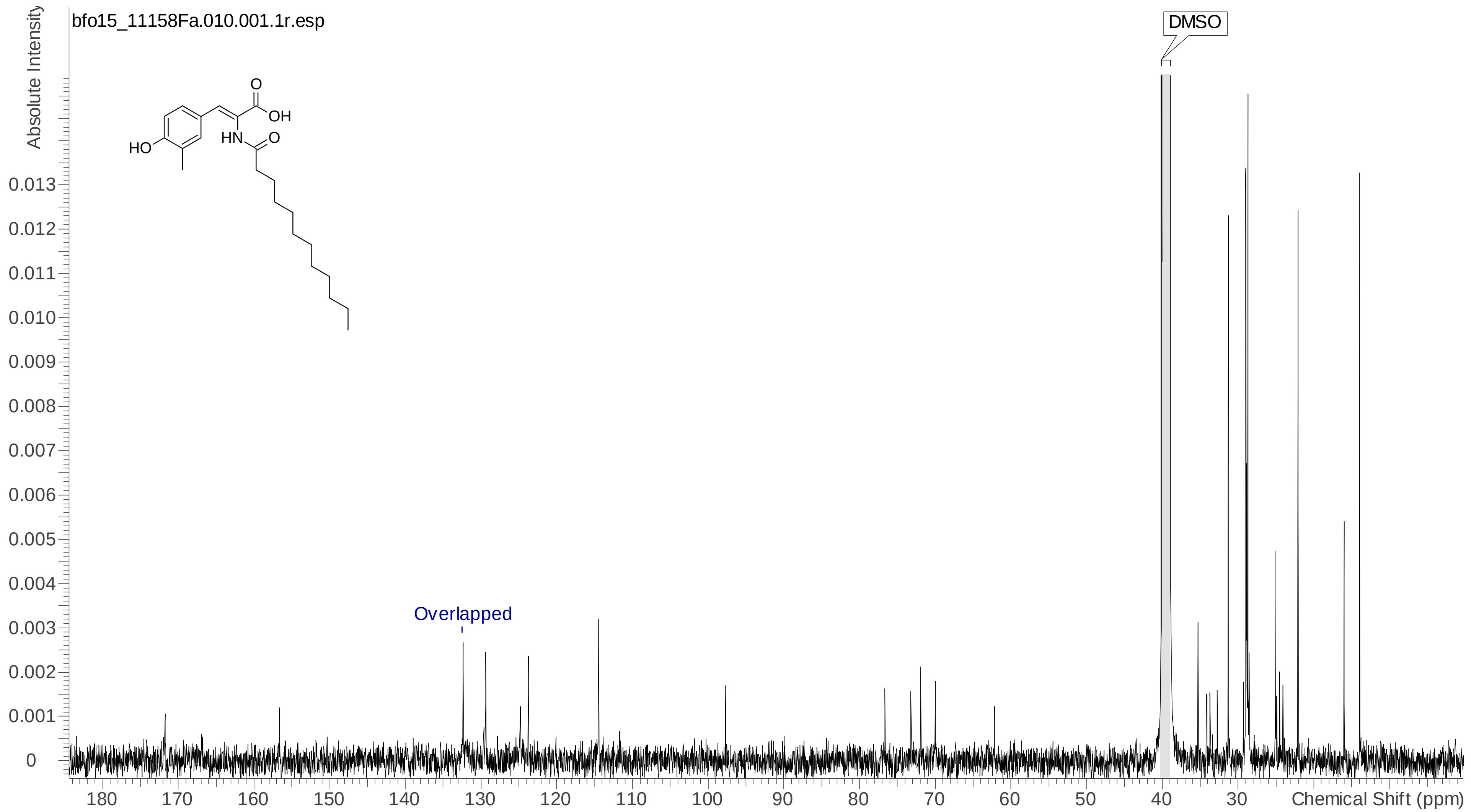

Supplementary Fig. 20. HSQC NMR spectrum (500 MHz, DMSO-d_6) of stieleriacine B_1 (2)

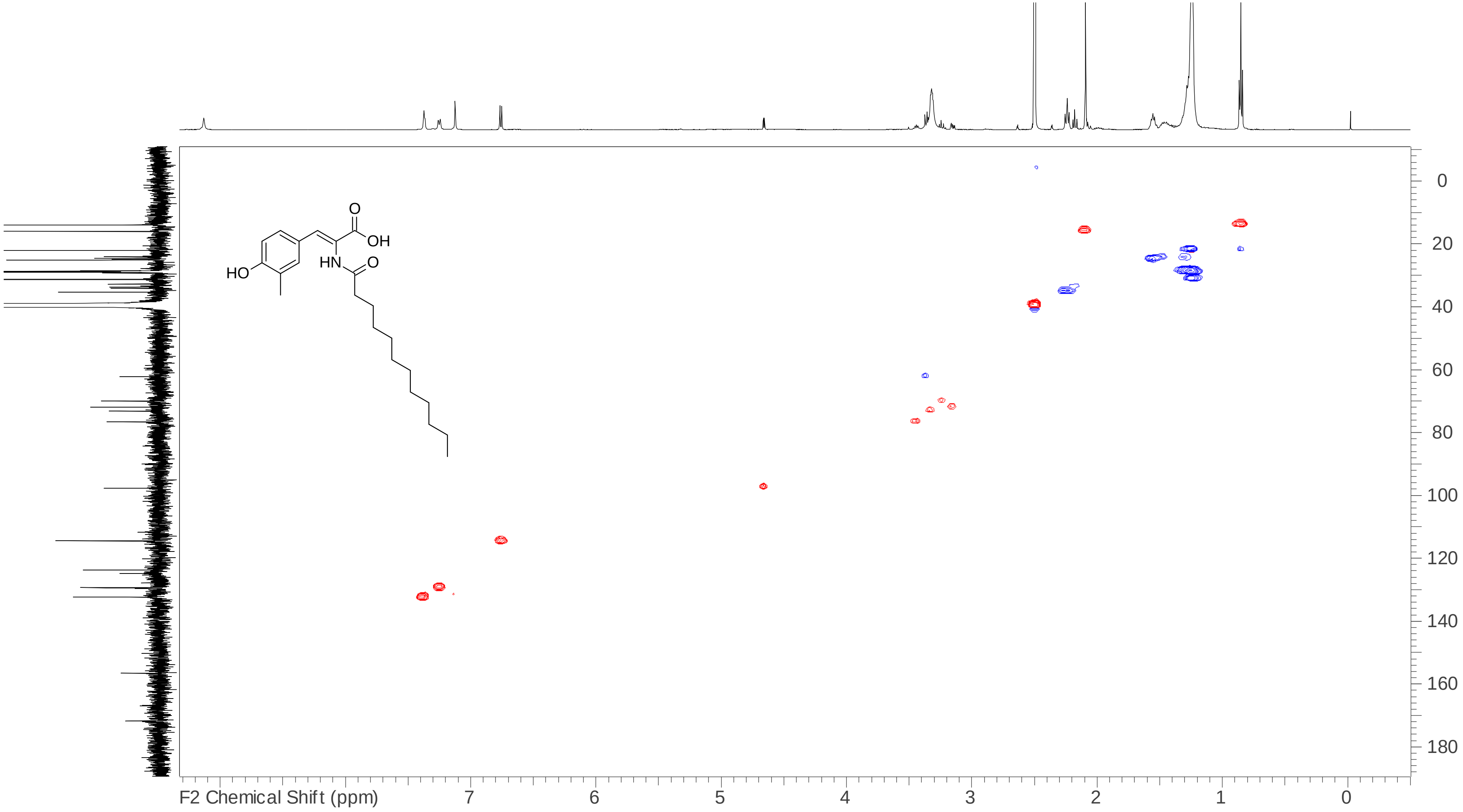

Supplementary Fig. 21. HMBC NMR spectrum (500 MHz, DMSO-d_6) of stieleriacine B_1 (2)

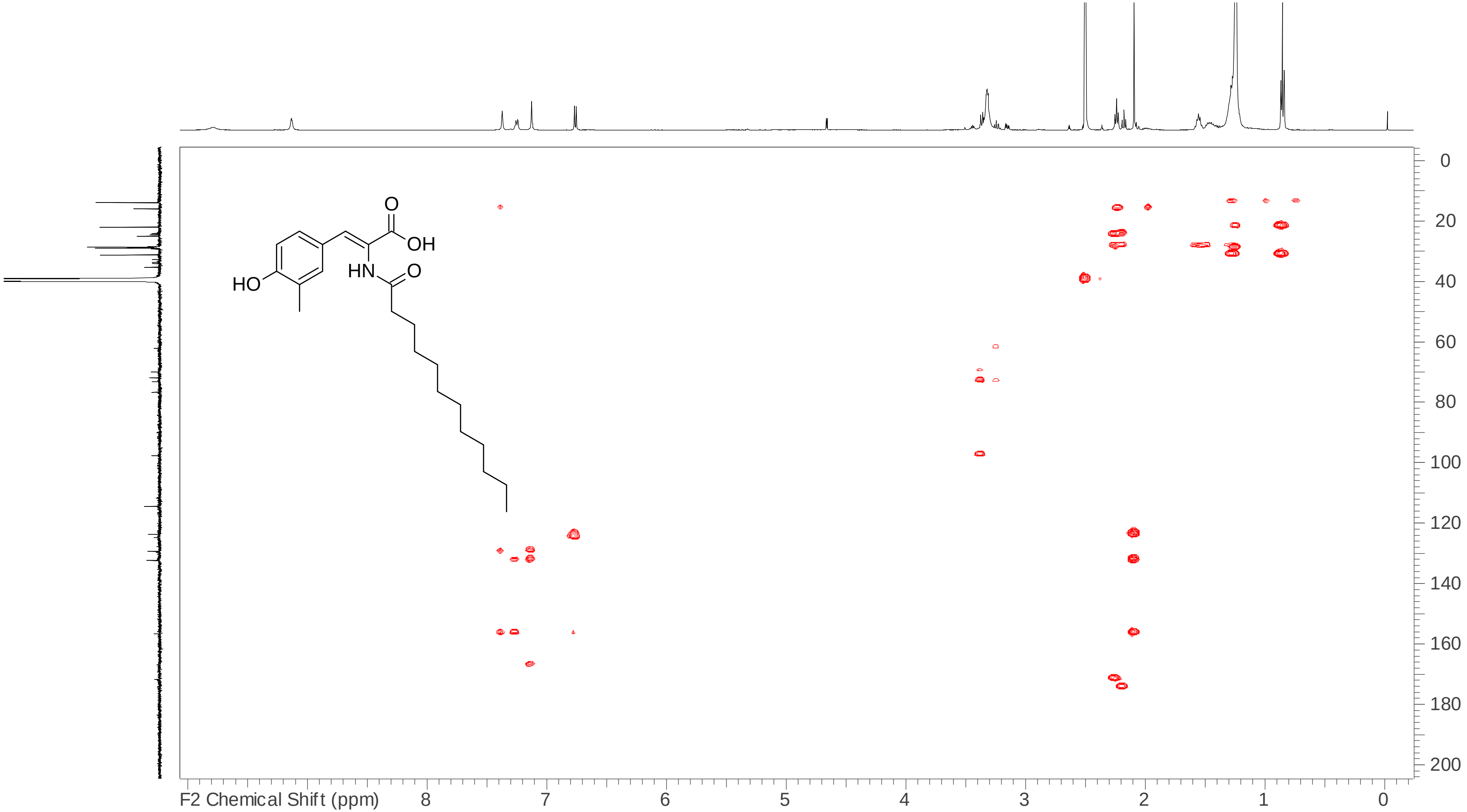

Supplementary Fig. 22. COSY NMR spectrum (500 MHz, DMSO-d_6) of stieleriacine B_1 (2)

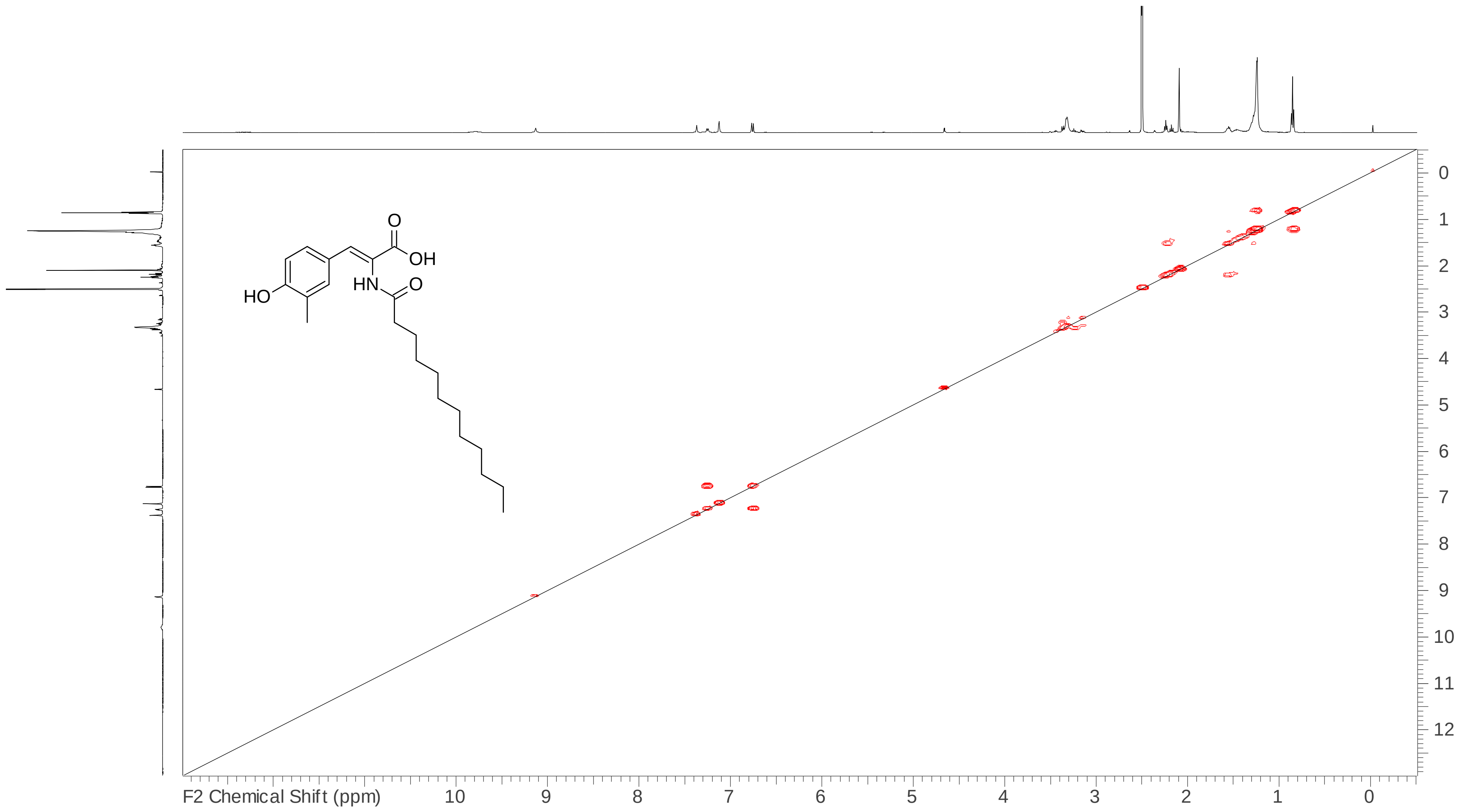

Supplementary Fig. 23. TOCSY NMR spectrum (500 MHz, DMSO-d_6) of stieleriacine B_1 (2)

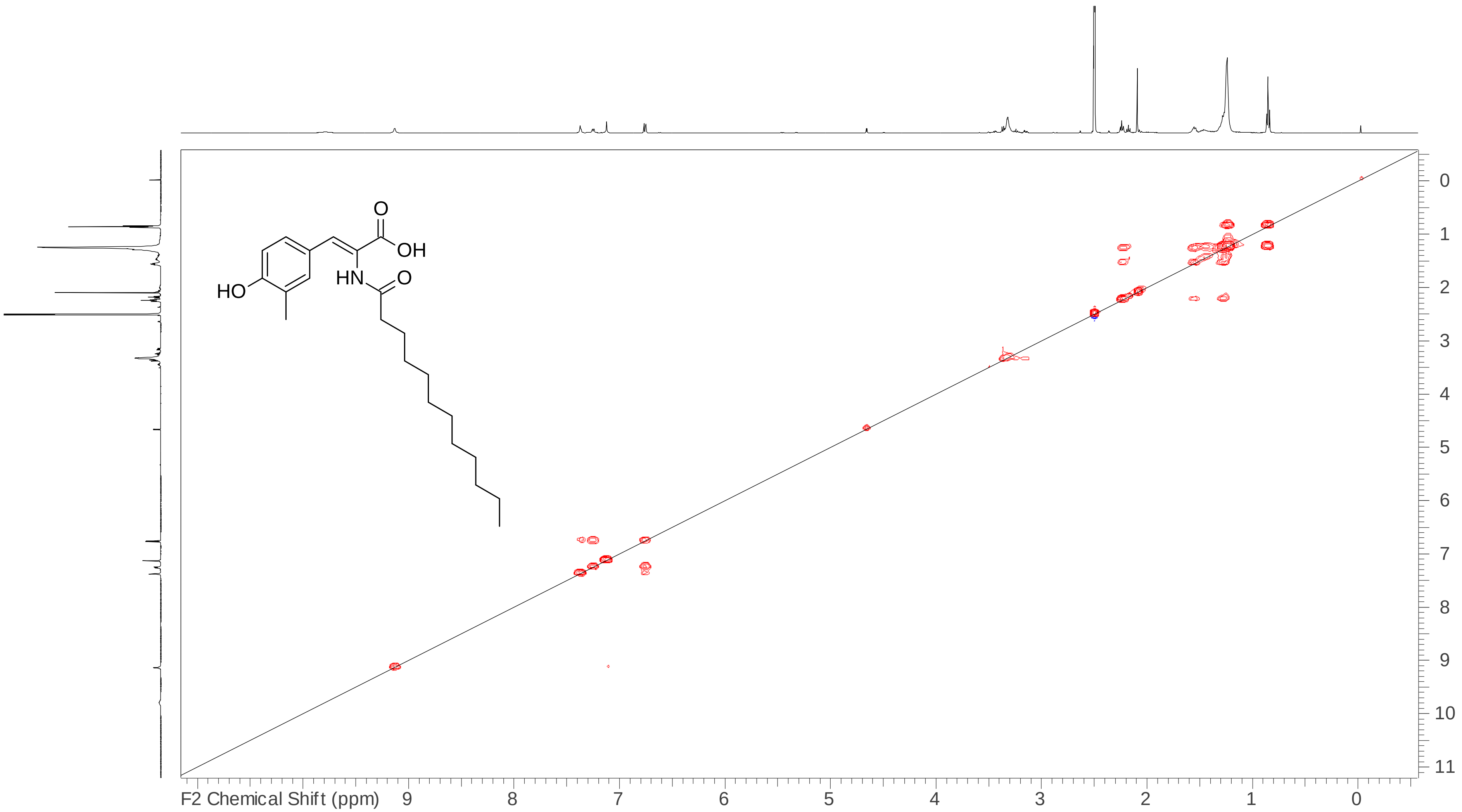

Supplementary Fig. 24. ROESY NMR spectrum (500 MHz, DMSO-d_6) of stieleriacine B_1 (2)

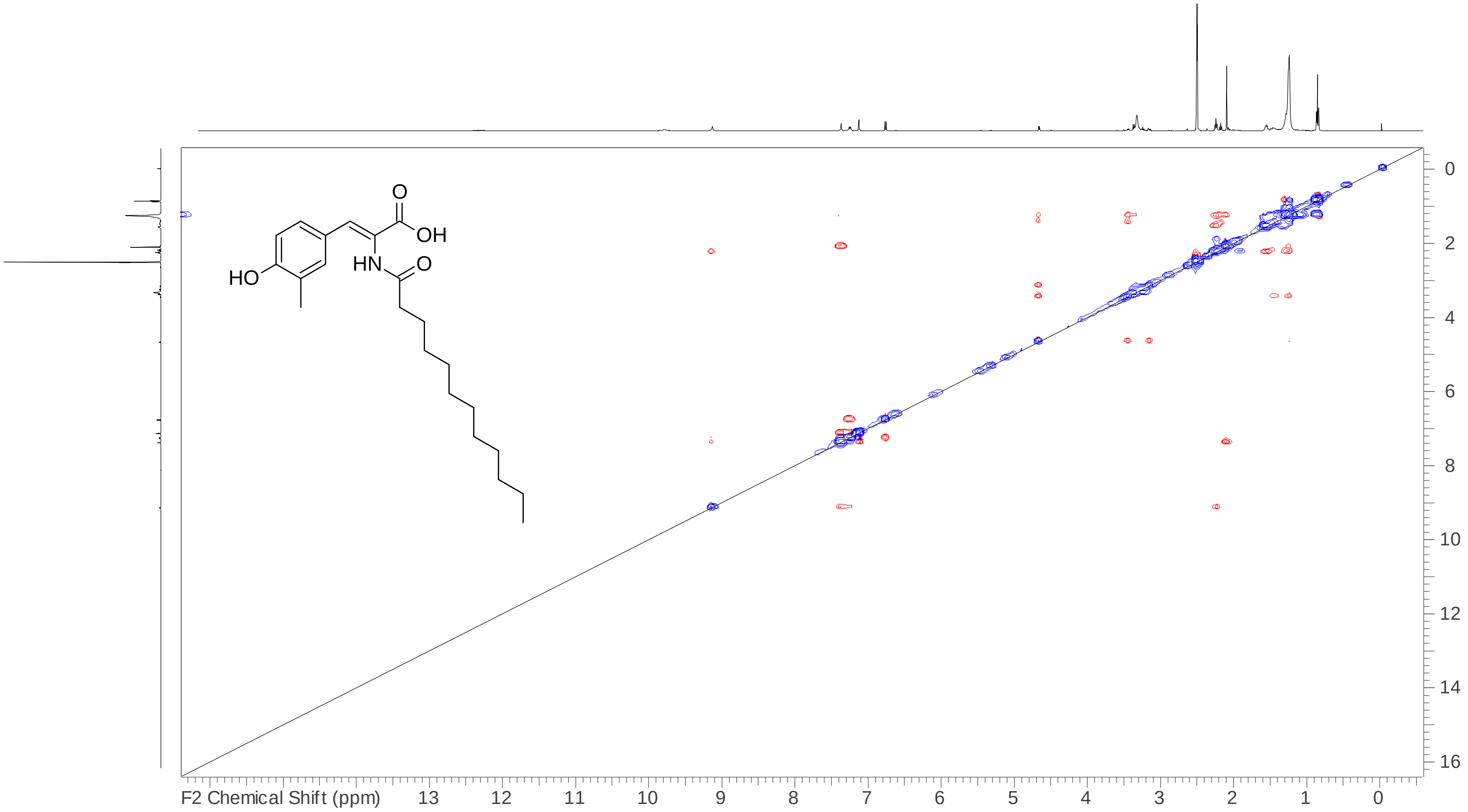

Supplementary Fig. 25. HRESIMS data of stieleriacine C (3)

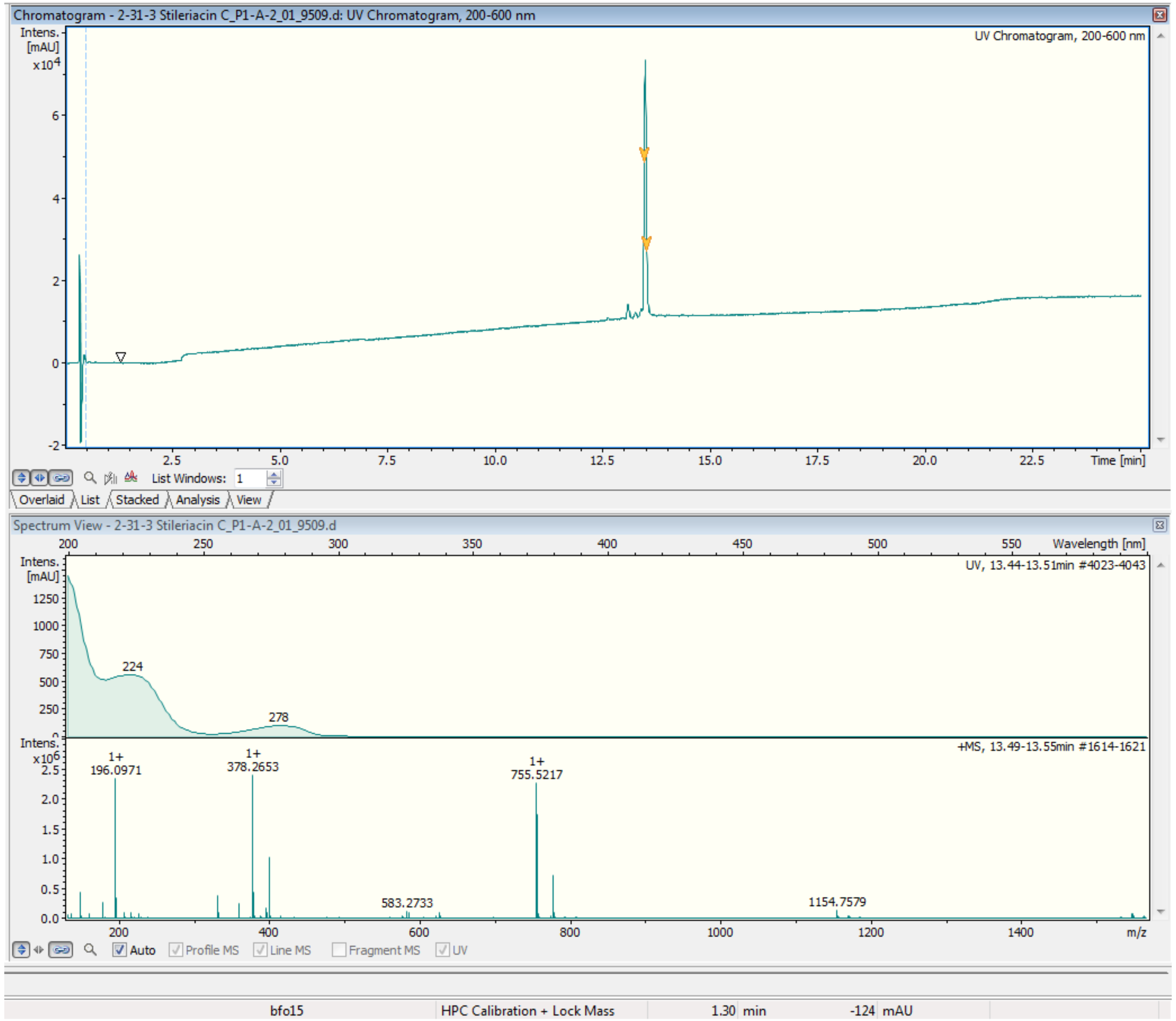

Supplementary Fig. 26. ^{1}H NMR spectrum (700 MHz, DMSO-d_6) of stieleriacine C (3)

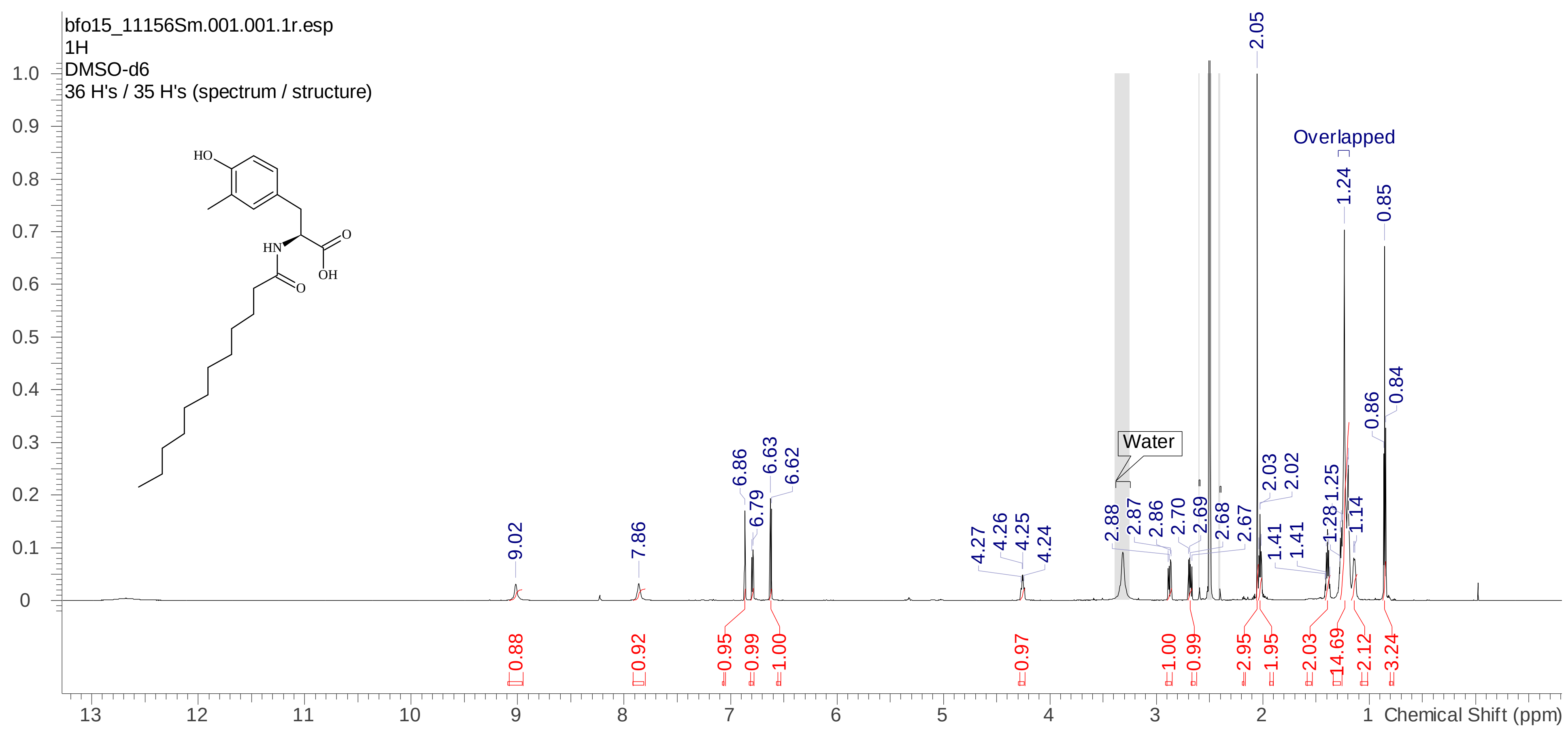

Supplementary Fig. 27. ^{13}C NMR spectrum (176 MHz, DMSO-d_6) of stieleriacine C (3)

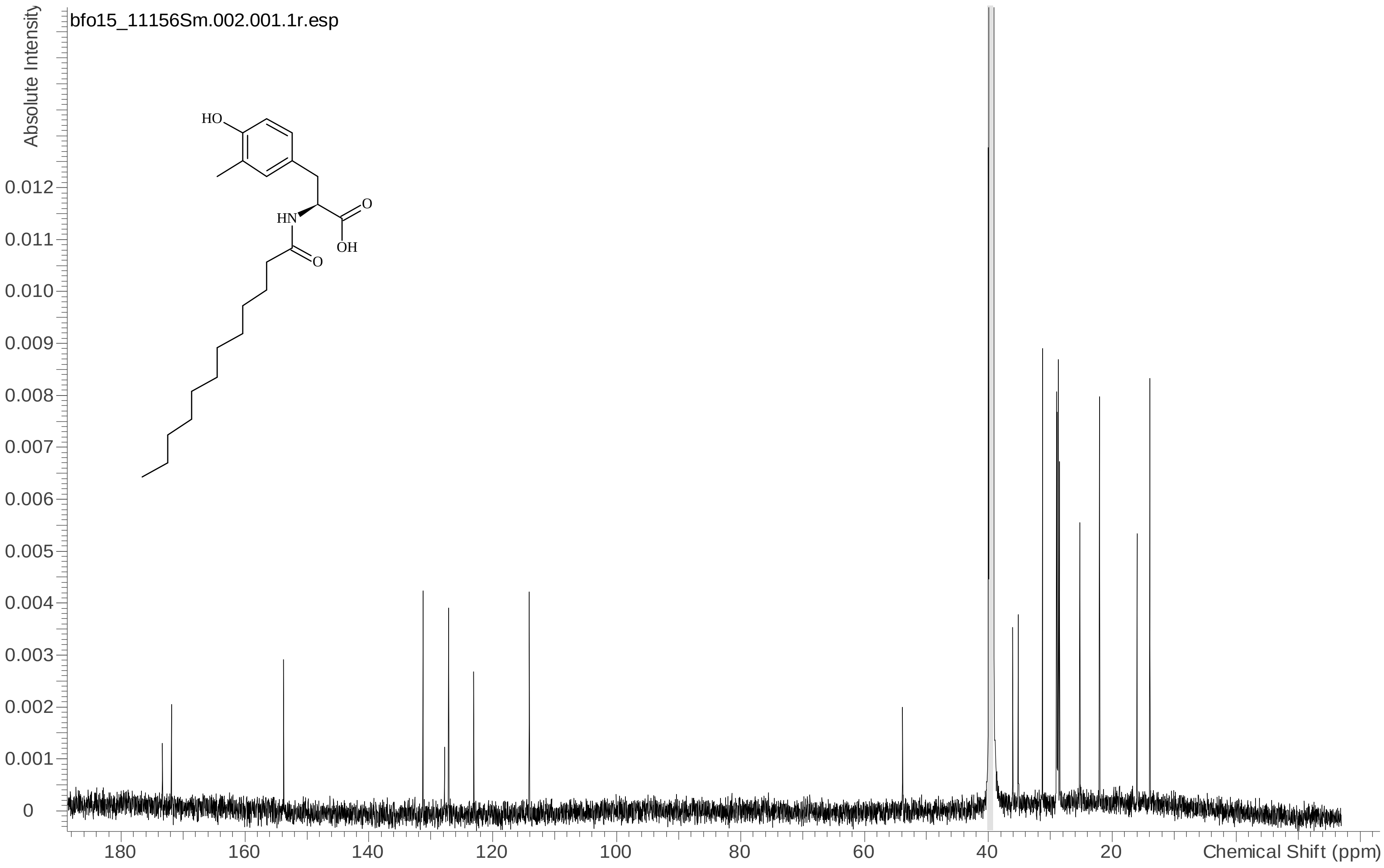

Supplementary Fig. 28. HSQC NMR spectrum (700 MHz, DMSO-d_6) of stieleriacine C (3)

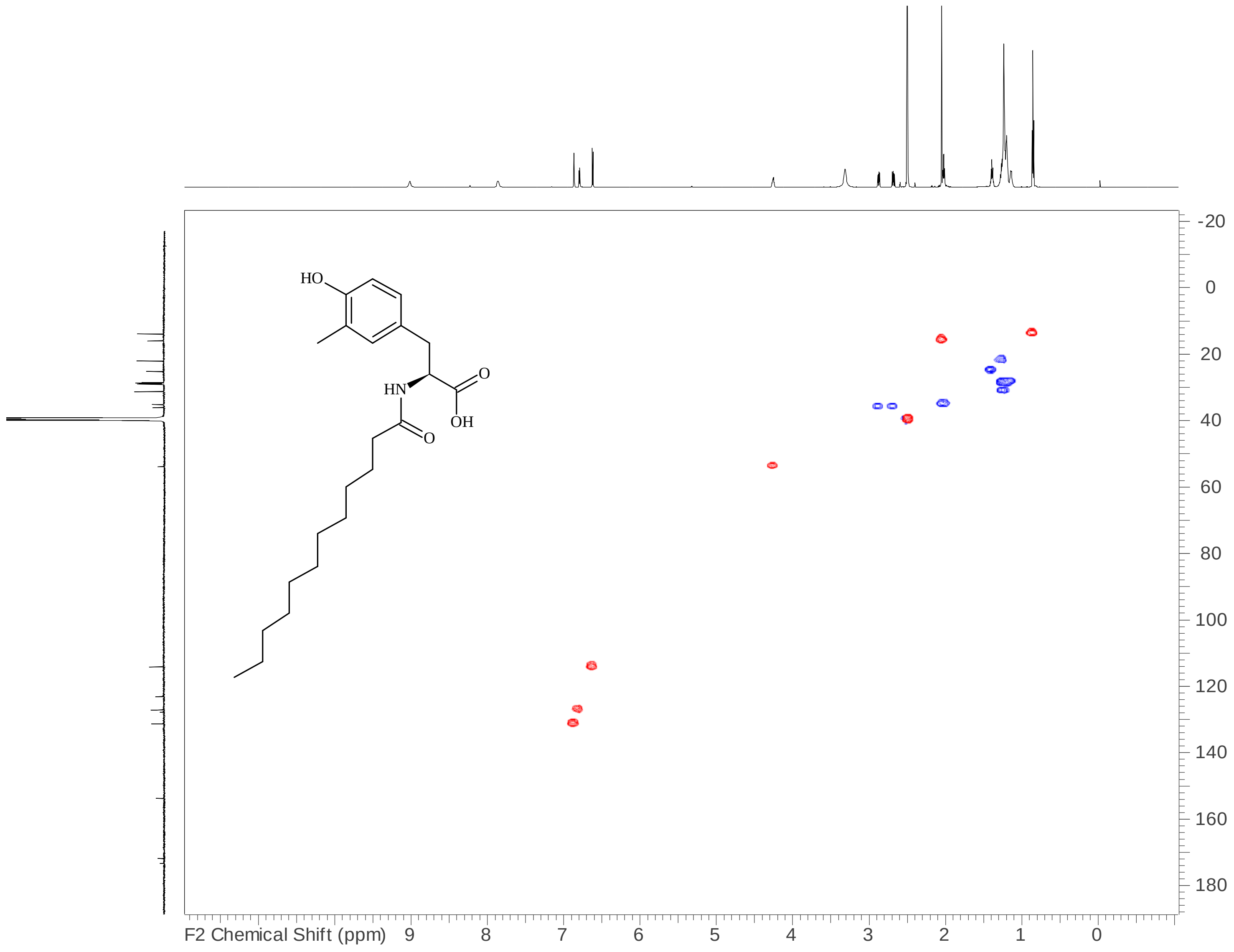

Supplementary Fig. 29. HMBC NMR spectrum (700 MHz, DMSO-d_6) of stieleriacine C (3)

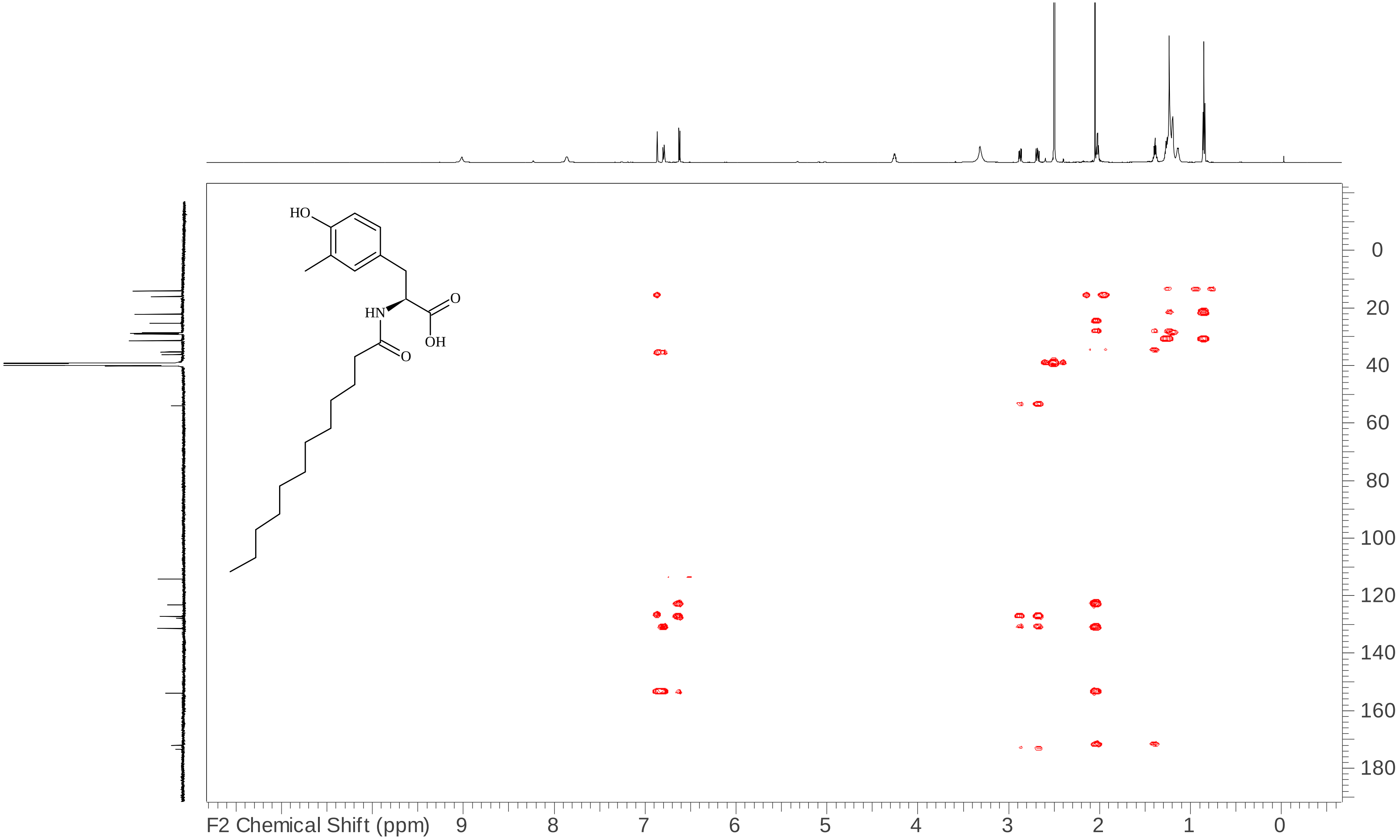

Supplementary Fig. 30. COSY NMR spectrum (700 MHz, DMSO-d_6) of stieleriacine C (3)

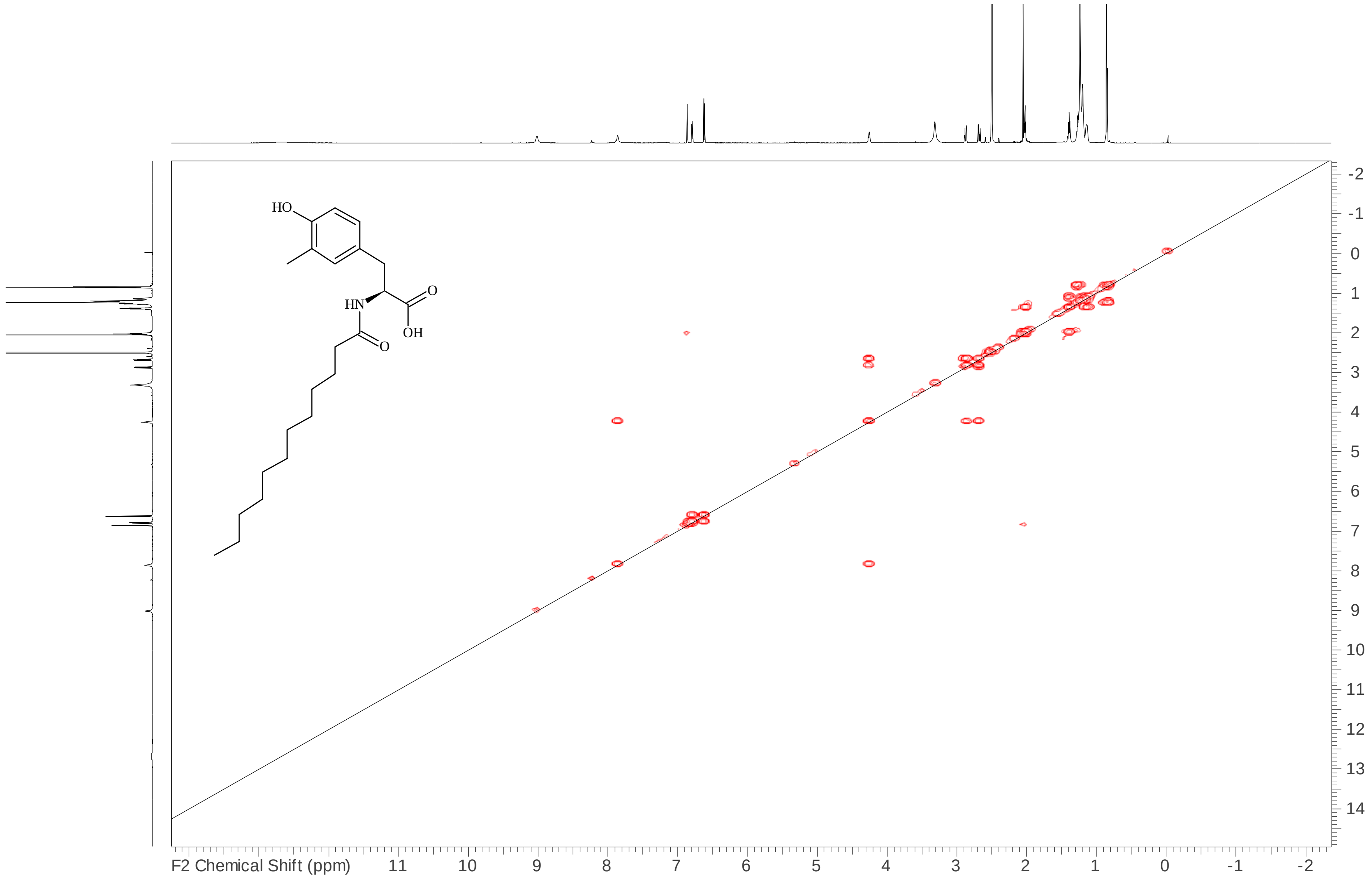

Supplementary Fig. 31. TOCSY NMR spectrum (700 MHz, DMSO-d_6) of stieleriacine C (3)

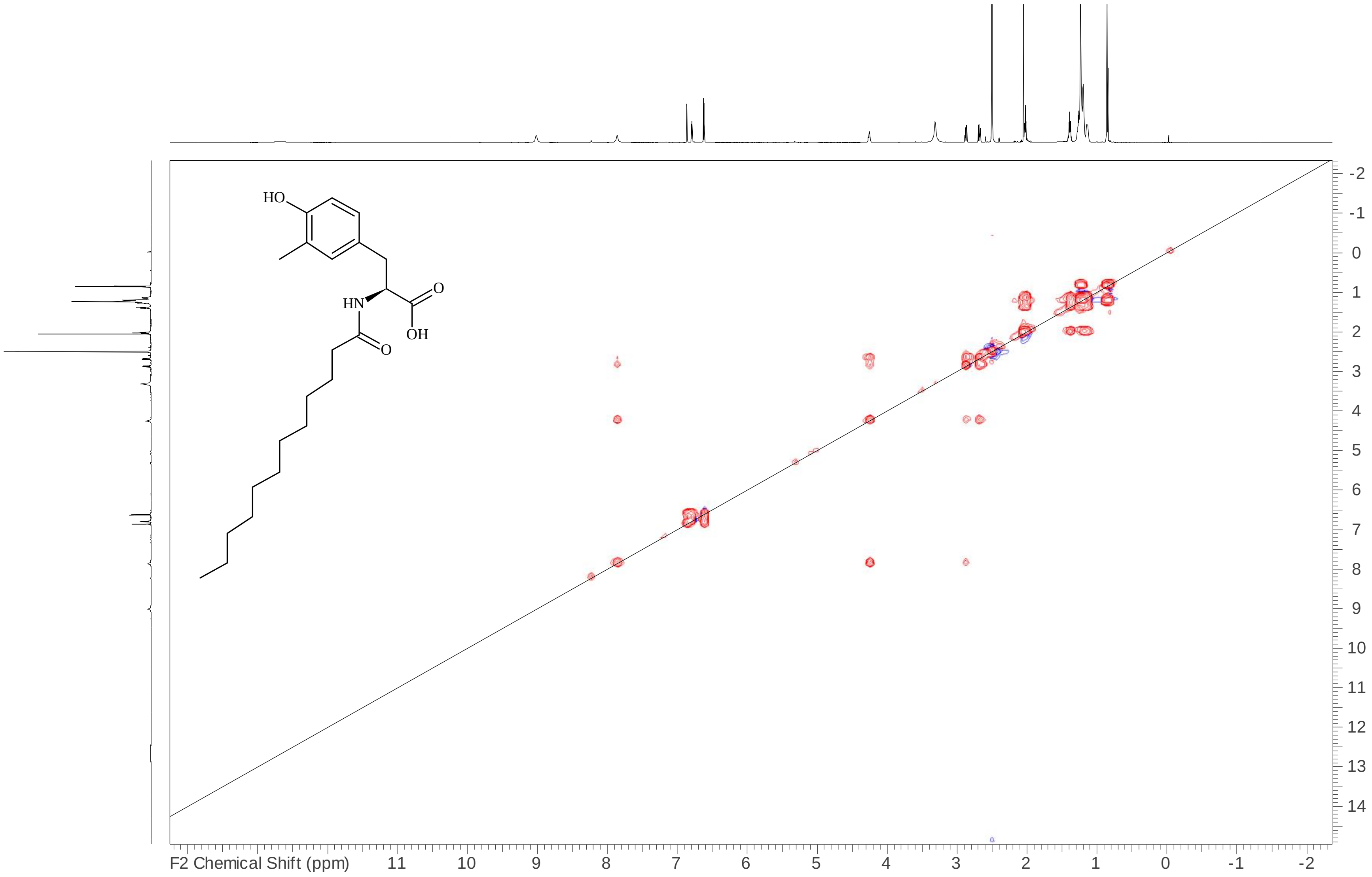

Supplementary Fig. 32. ROESY NMR spectrum (700 MHz, DMSO-d_6) of stieleriacine C (3)

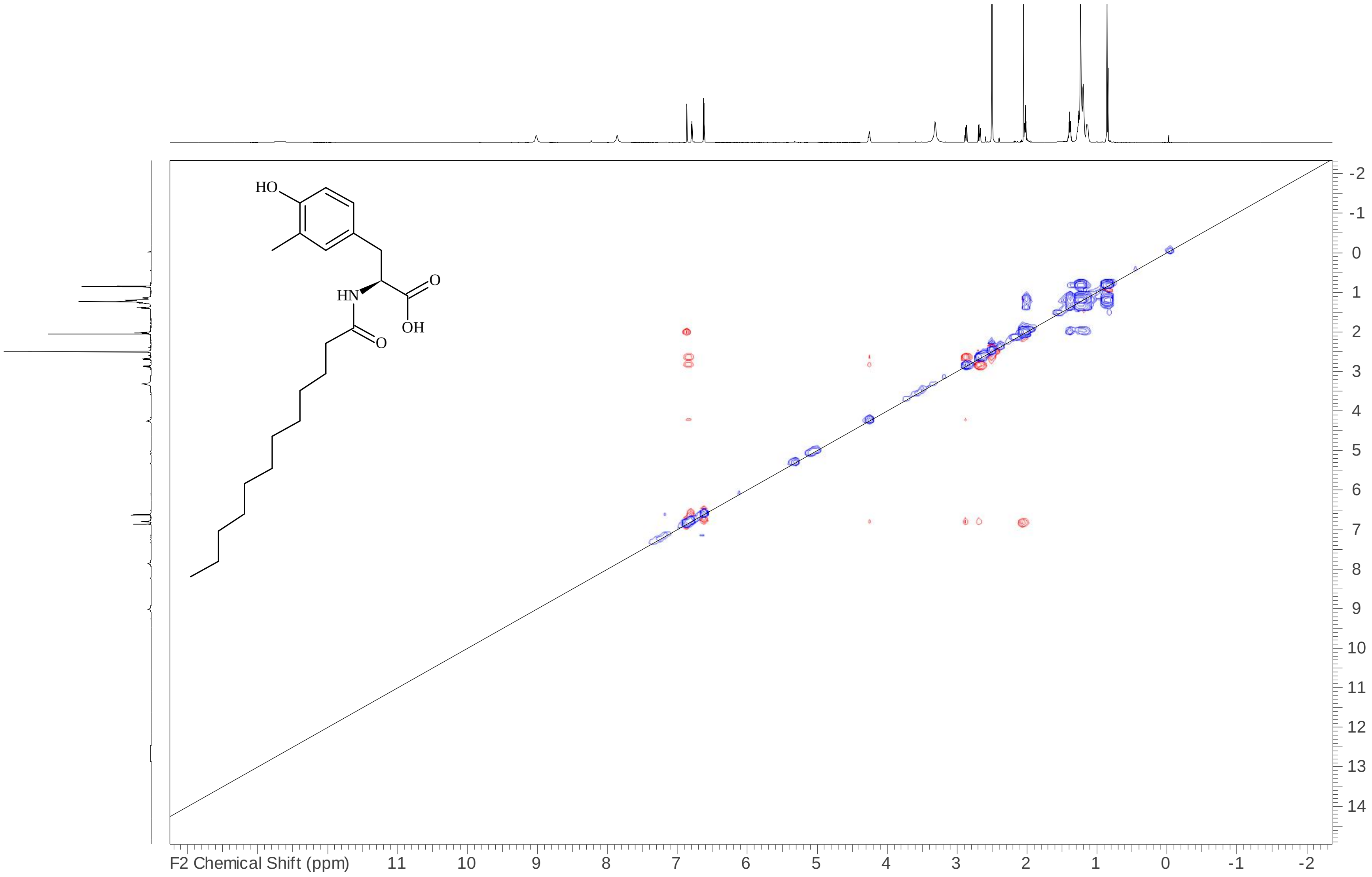

Supplementary Fig. 33. HRESIMS data of stieleriacine A_2 (4)

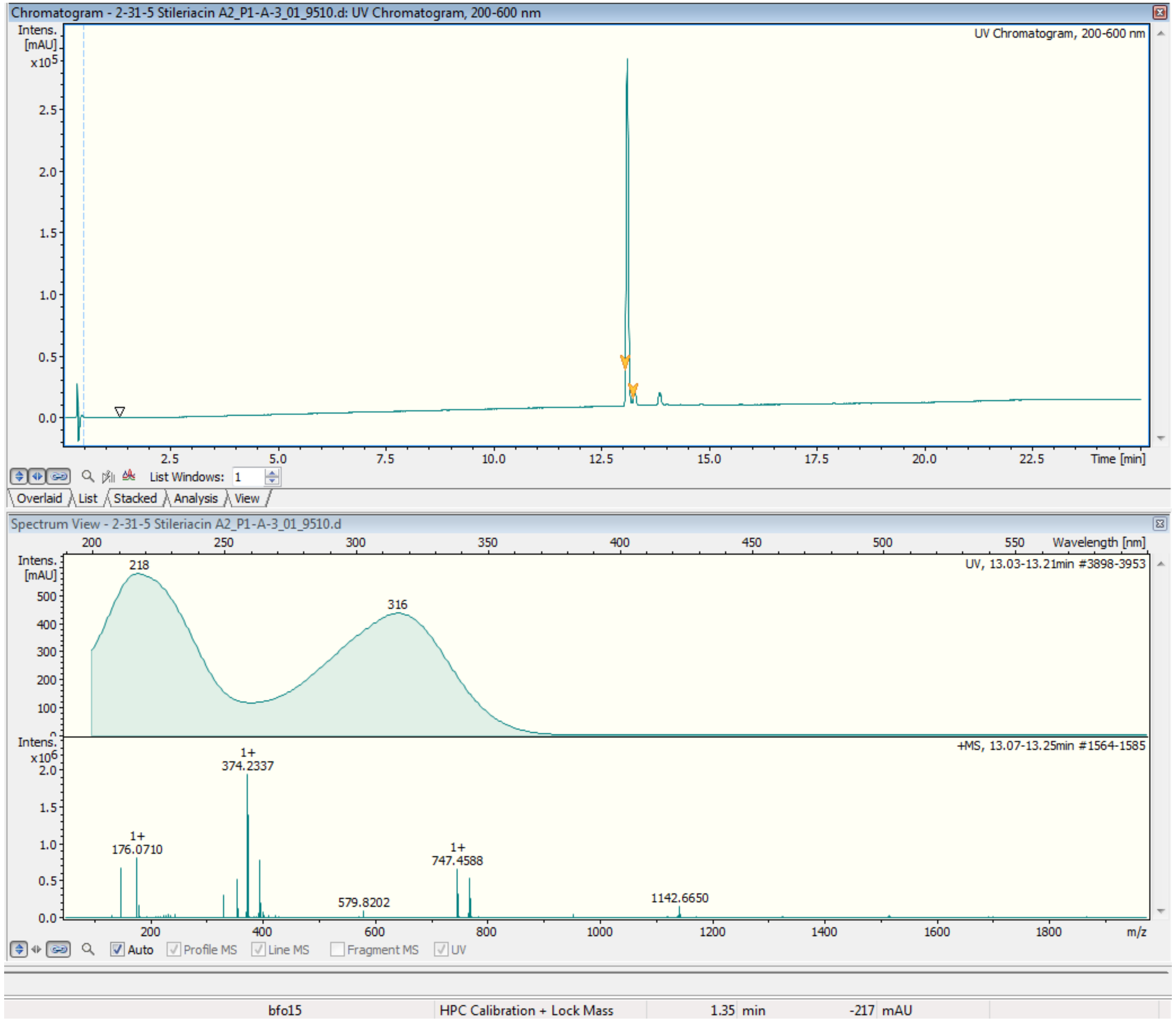

Supplementary Fig. 34. ¹H NMR spectrum (500 MHz, DMSO-d_6) of stieleriacine A_2 (4)

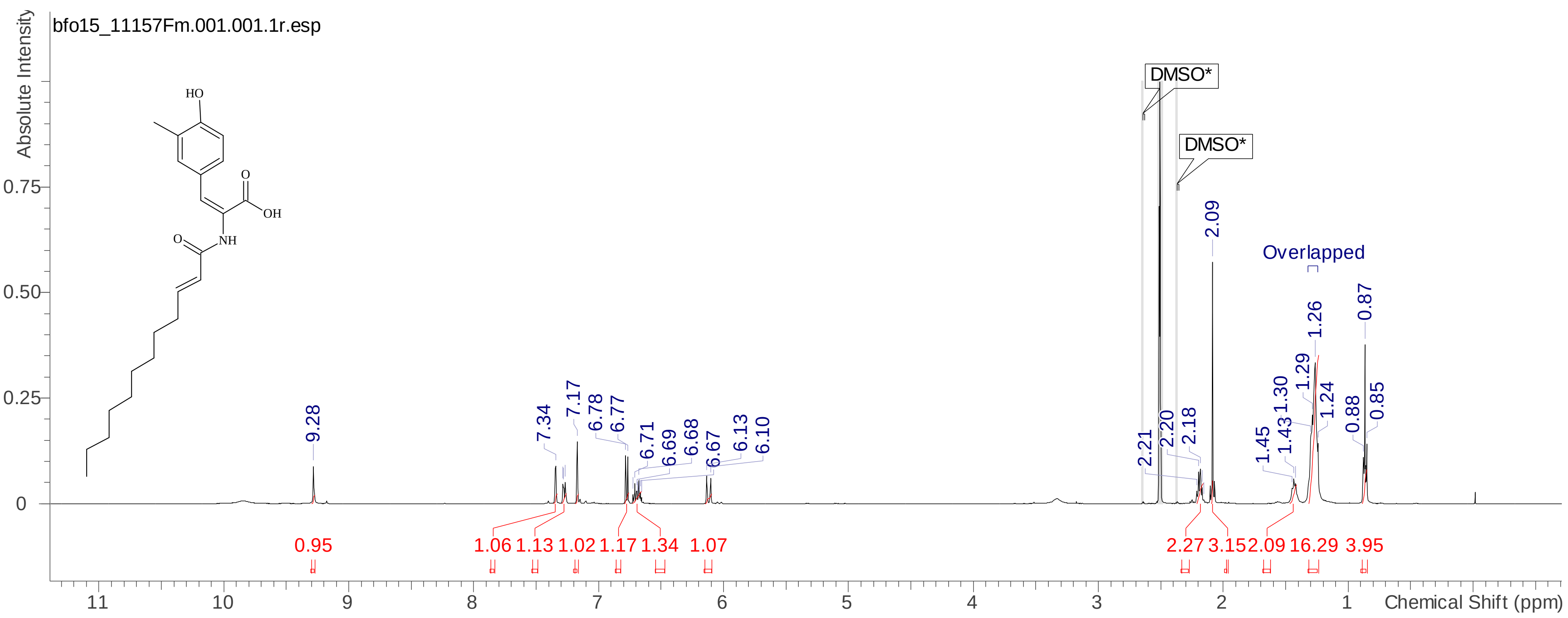

Supplementary Fig. 35. ^{13}C NMR spectrum (126 MHz, DMSO-d_6) of stieleriacine A_2 (4)

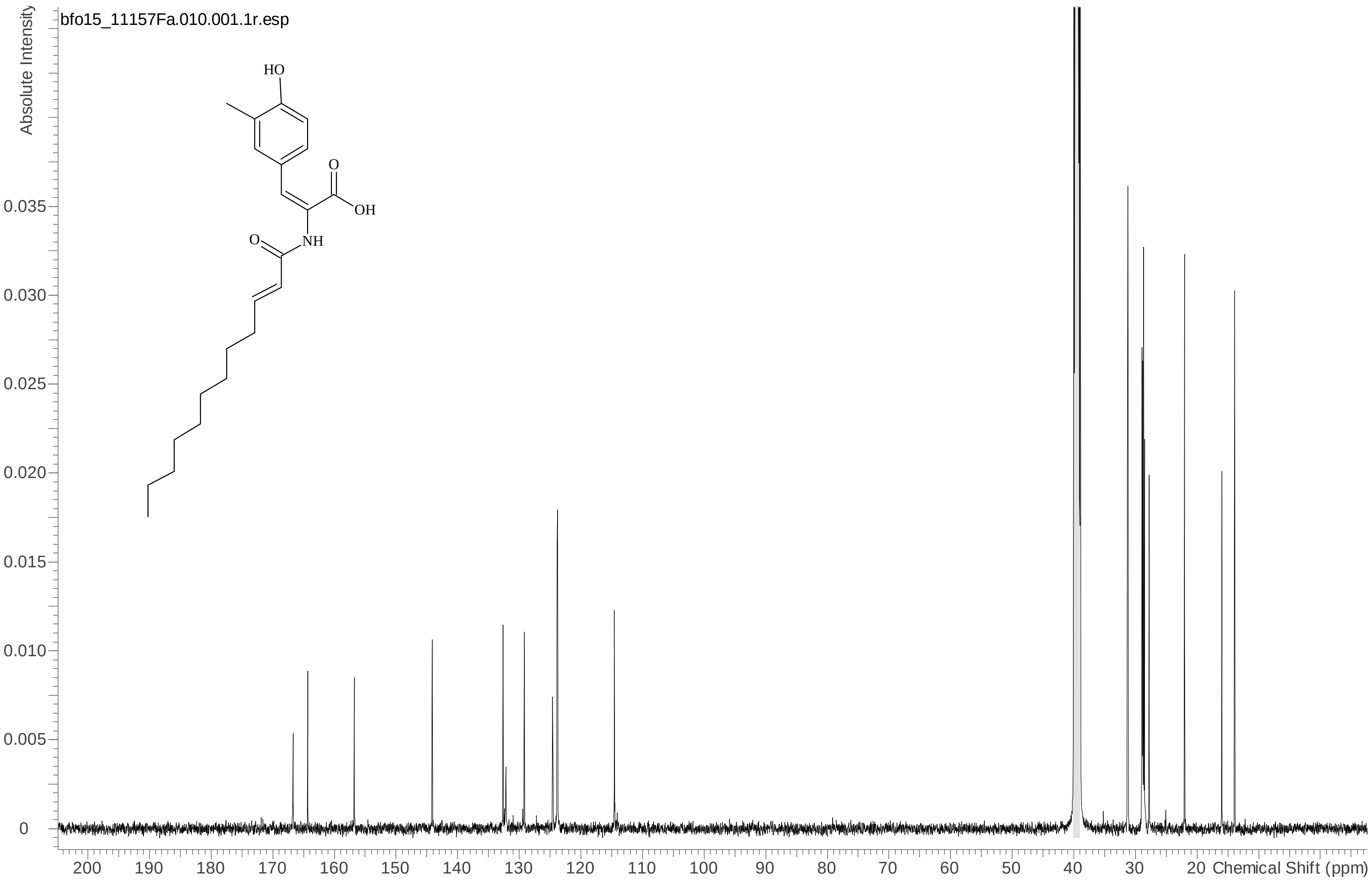

Supplementary Fig. 36. HSQC NMR spectrum (500 MHz, DMSO-d_6) of stieleriacine A_2 (4)

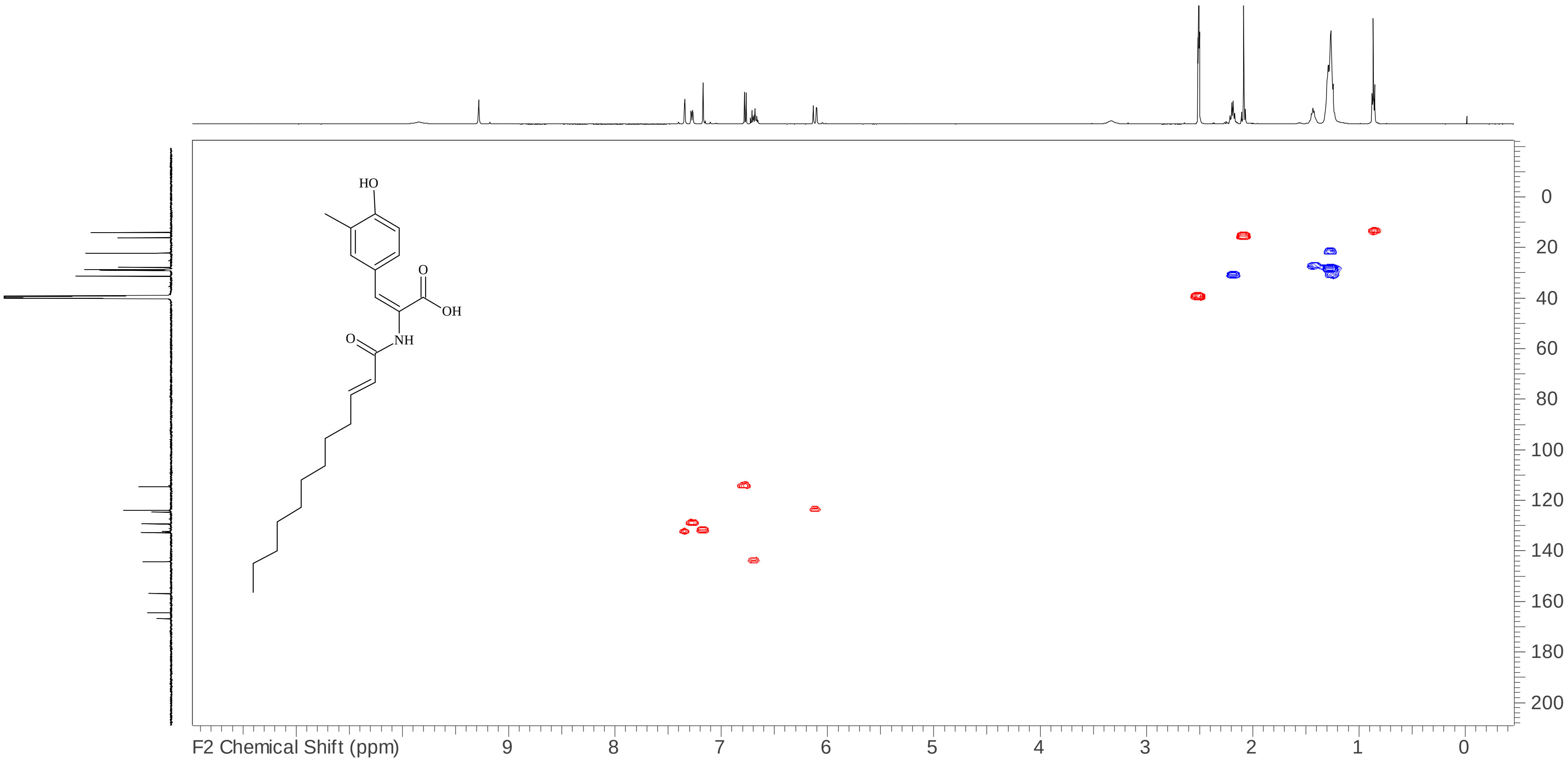

Supplementary Fig. 37. HMBC NMR spectrum (500 MHz, DMSO-d_6) of stieleriacine A_2 (4)

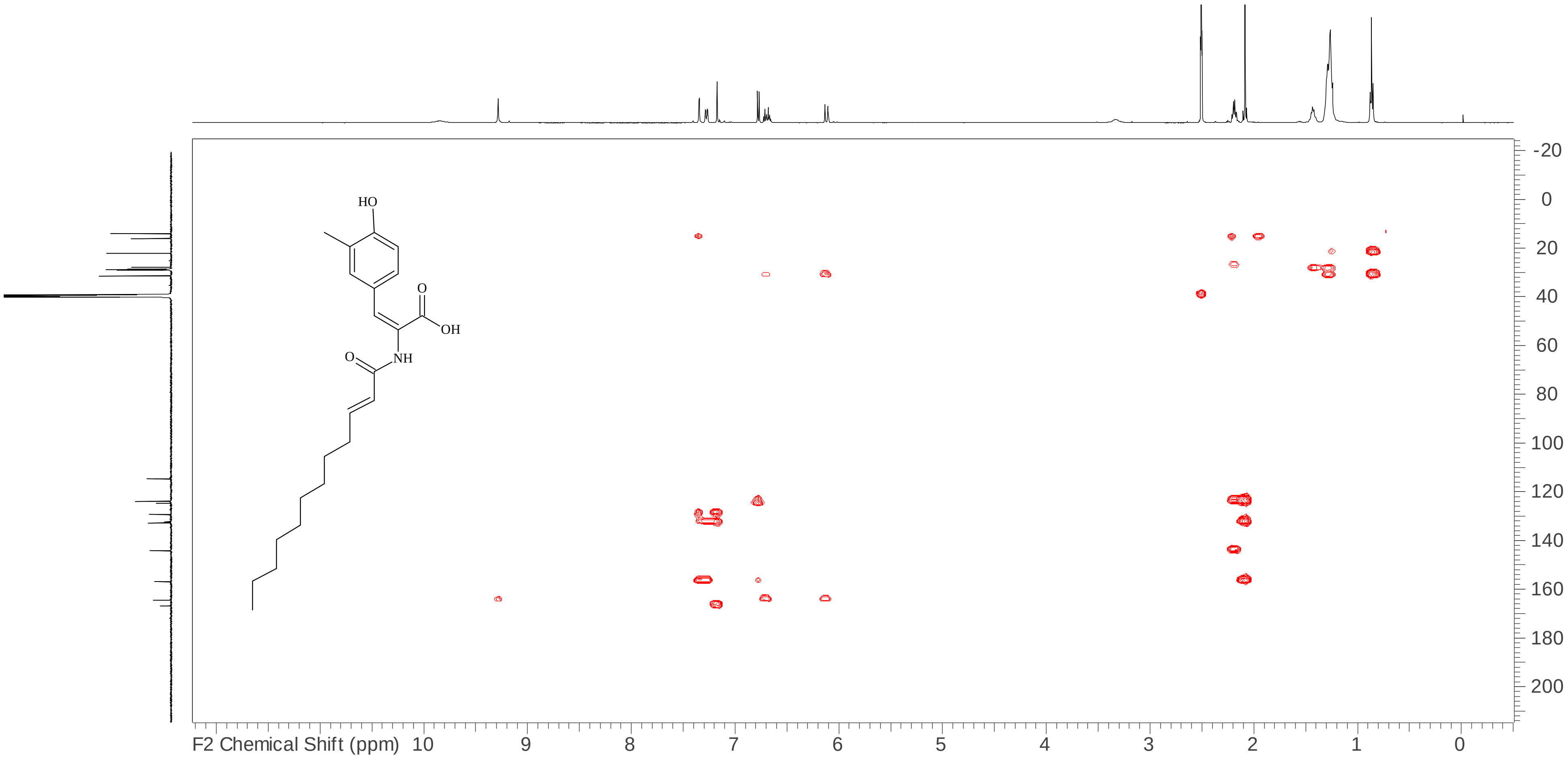

Supplementary Fig. 38. COSY NMR spectrum (500 MHz, DMSO-d_6) of stieleriacine A_2 (4)

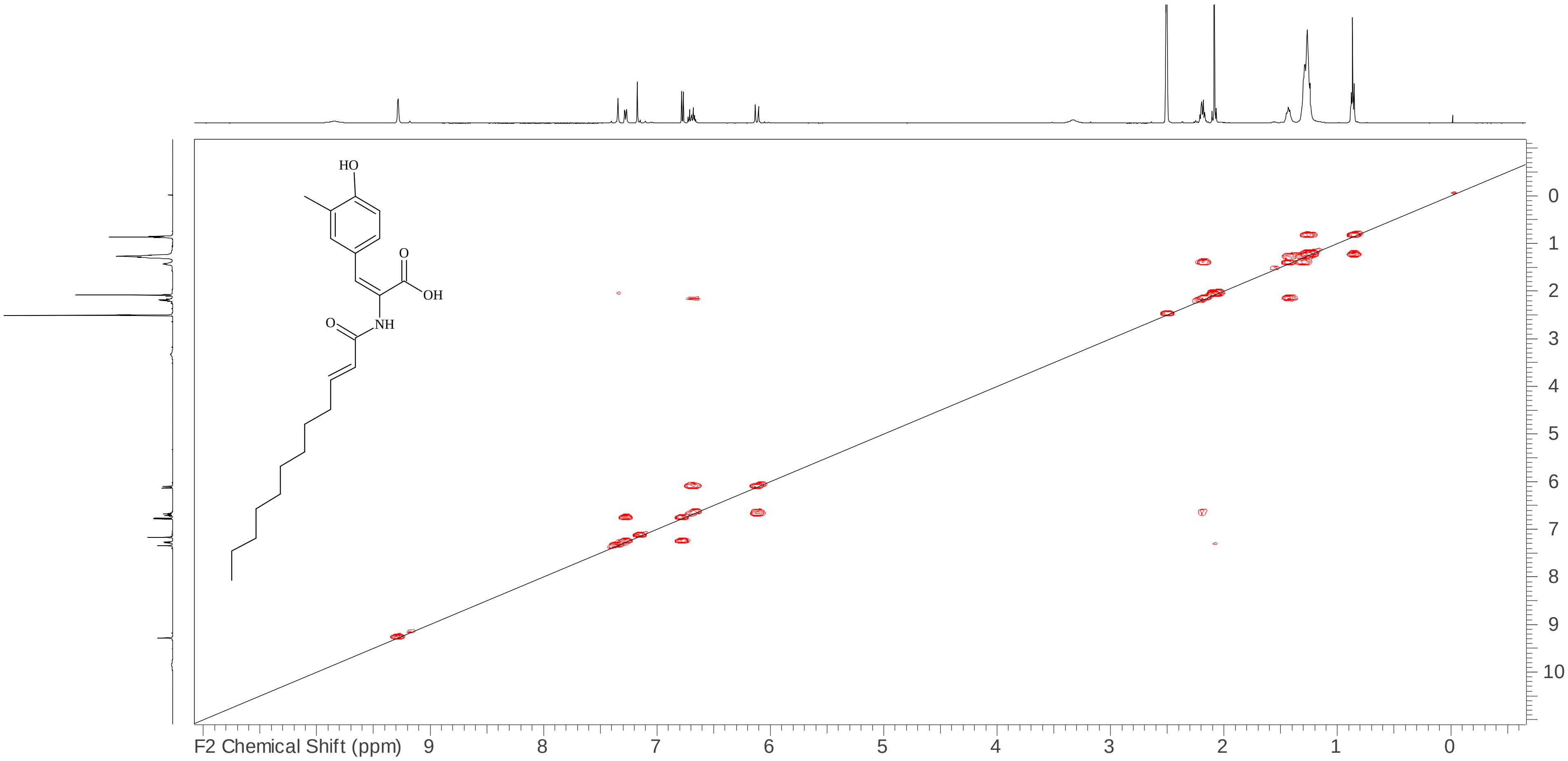

Supplementary Fig. 39. TOCSY NMR spectrum (500 MHz, DMSO-d_6) of stieleriacine A_2 (4)

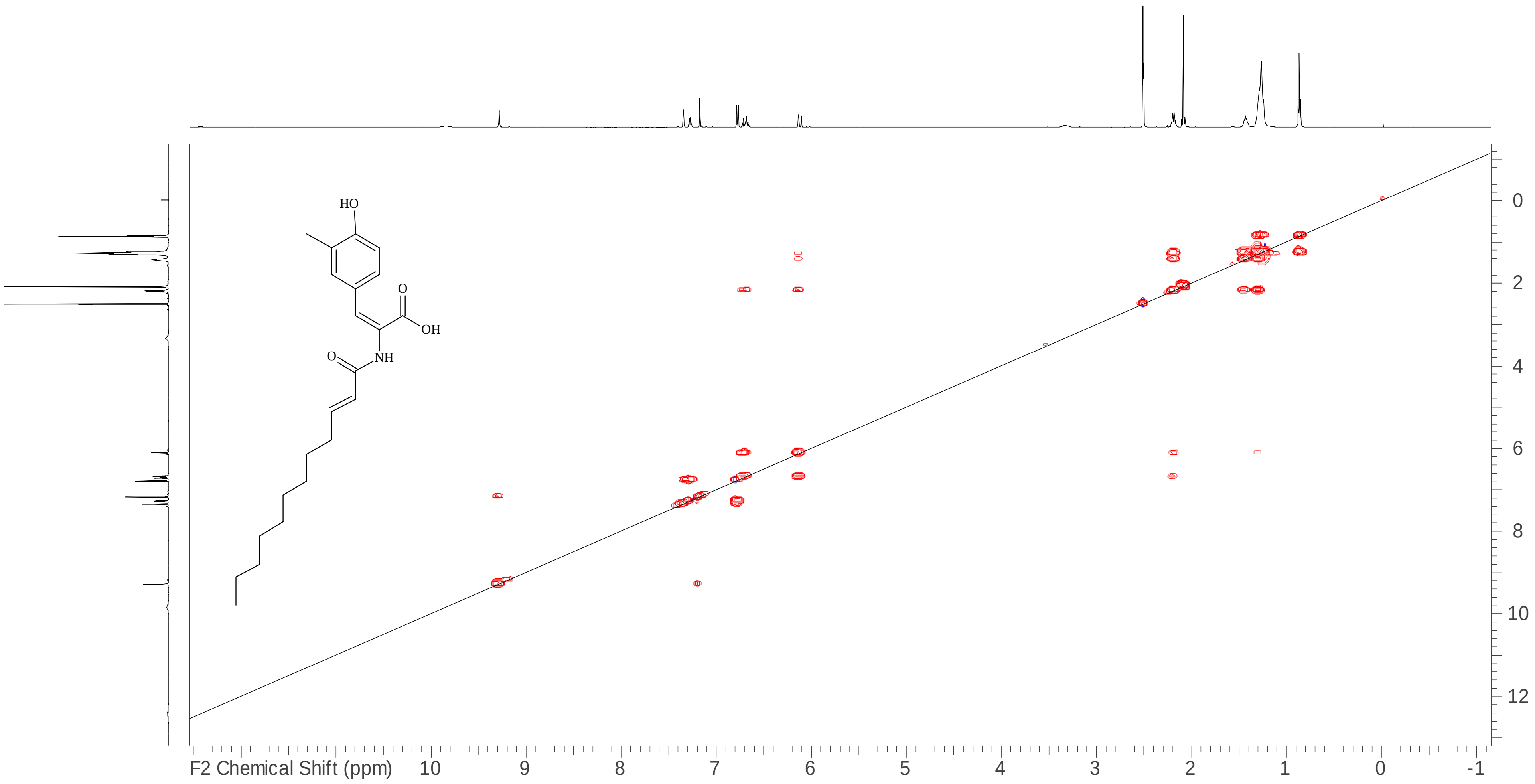

Supplementary Fig. 40. ROESY NMR spectrum (500 MHz, DMSO-d_6) of stieleriacine A_2 (4)

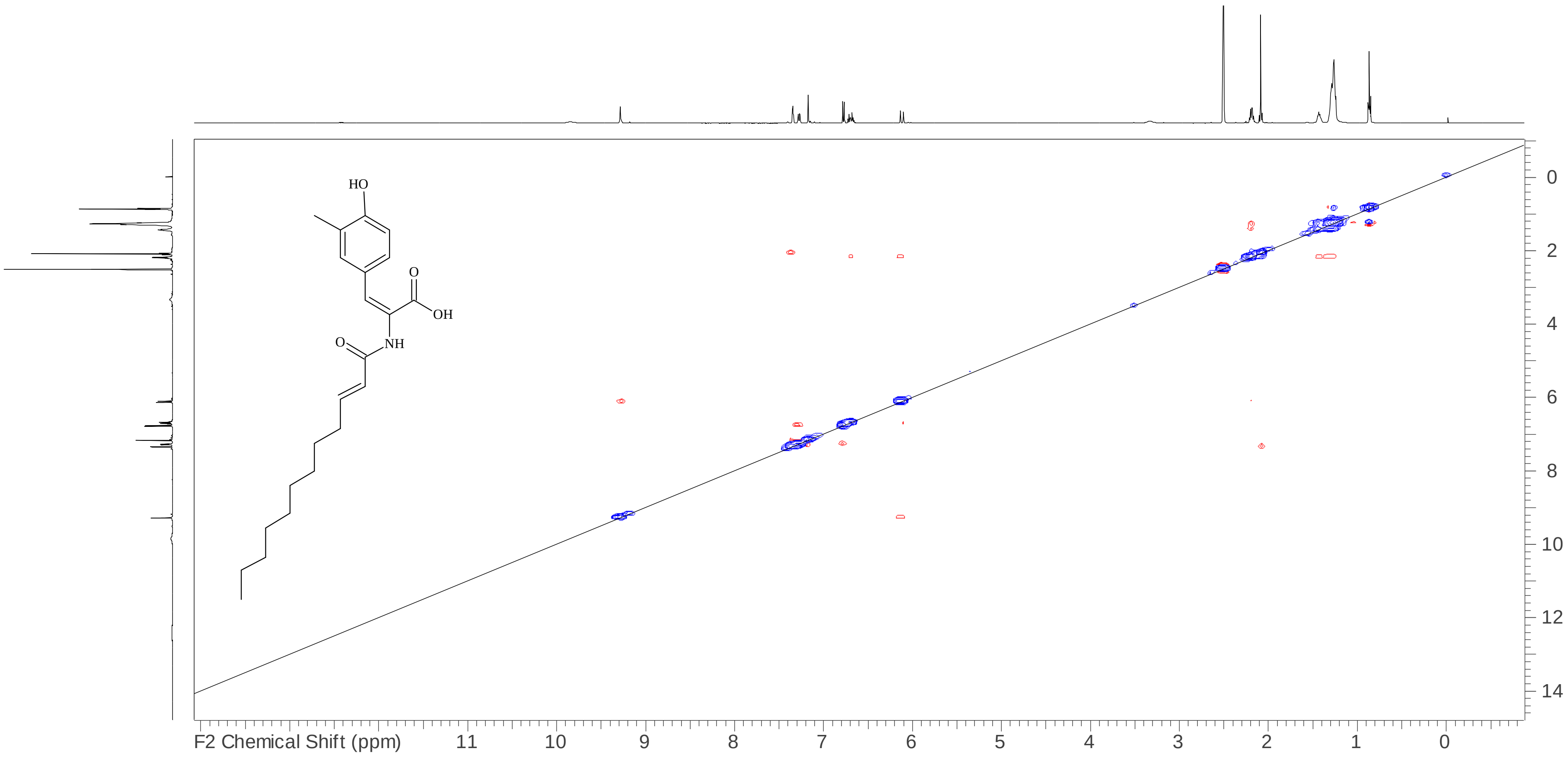

Supplementary Fig. 41. HRESIMS data of stieleriacine B_2 (5)

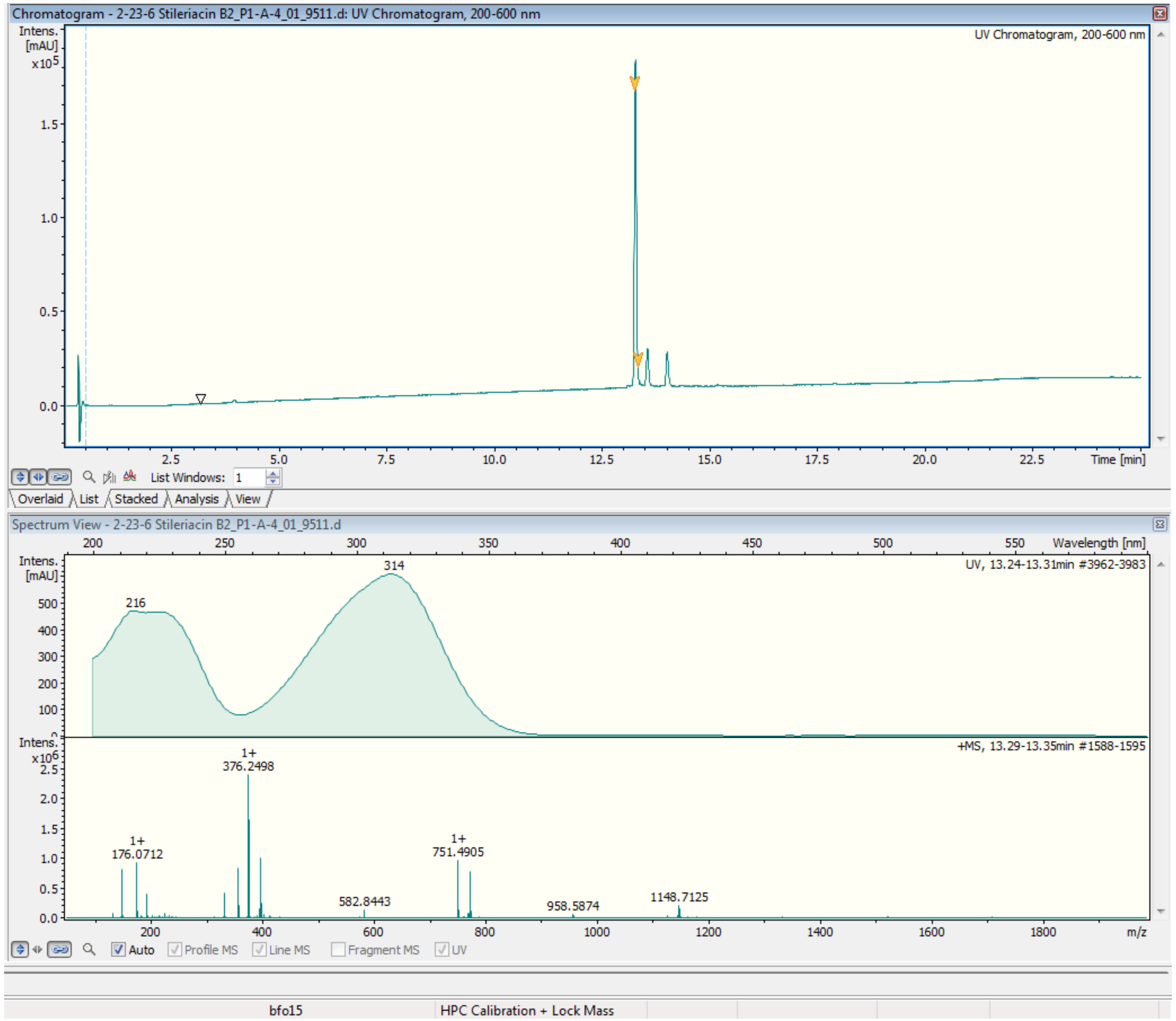

Supplementary Fig. 42. ^{1}**H NMR spectrum (500 MHz, DMSO-**d_6**) of stieleriacine B**$_2$ **(5)**

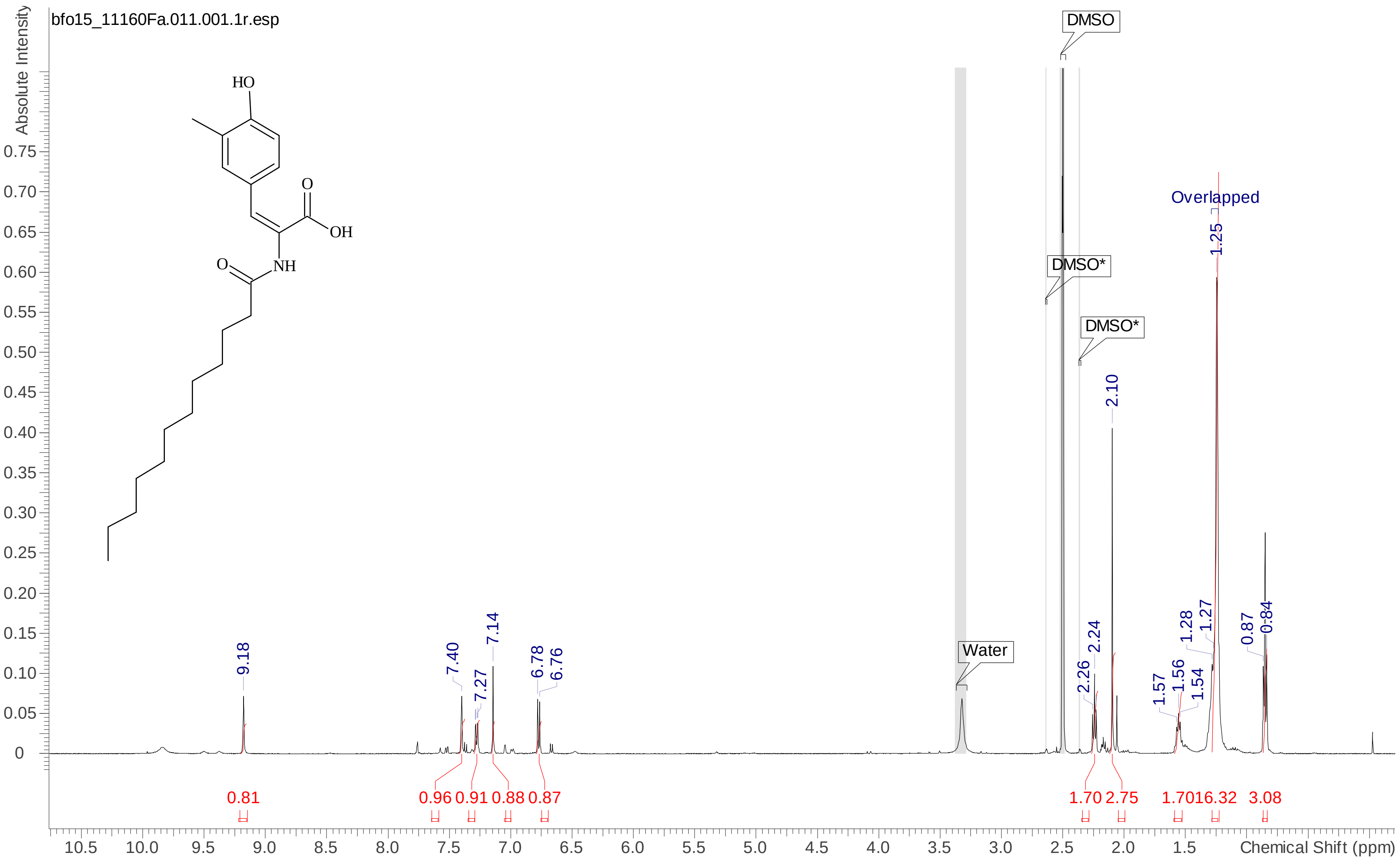

Supplementary Fig. 43. ^{13}C NMR spectrum (126 MHz, DMSO-d_6) of stieleriacine B_2 (5)

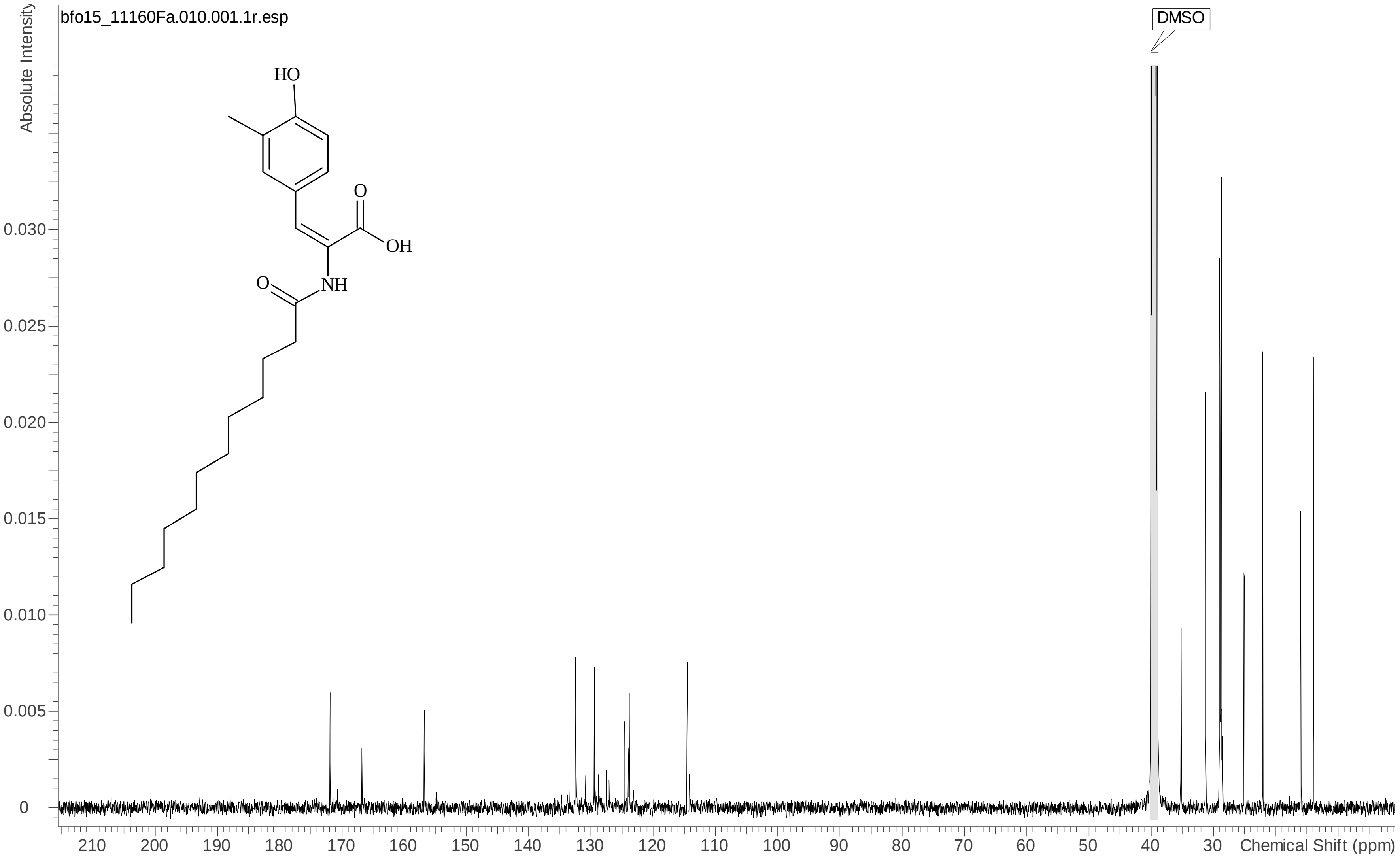

Supplementary Fig. 44. HSQC NMR spectrum (500 MHz, DMSO-d_6) of stieleriacine B_2 (5)

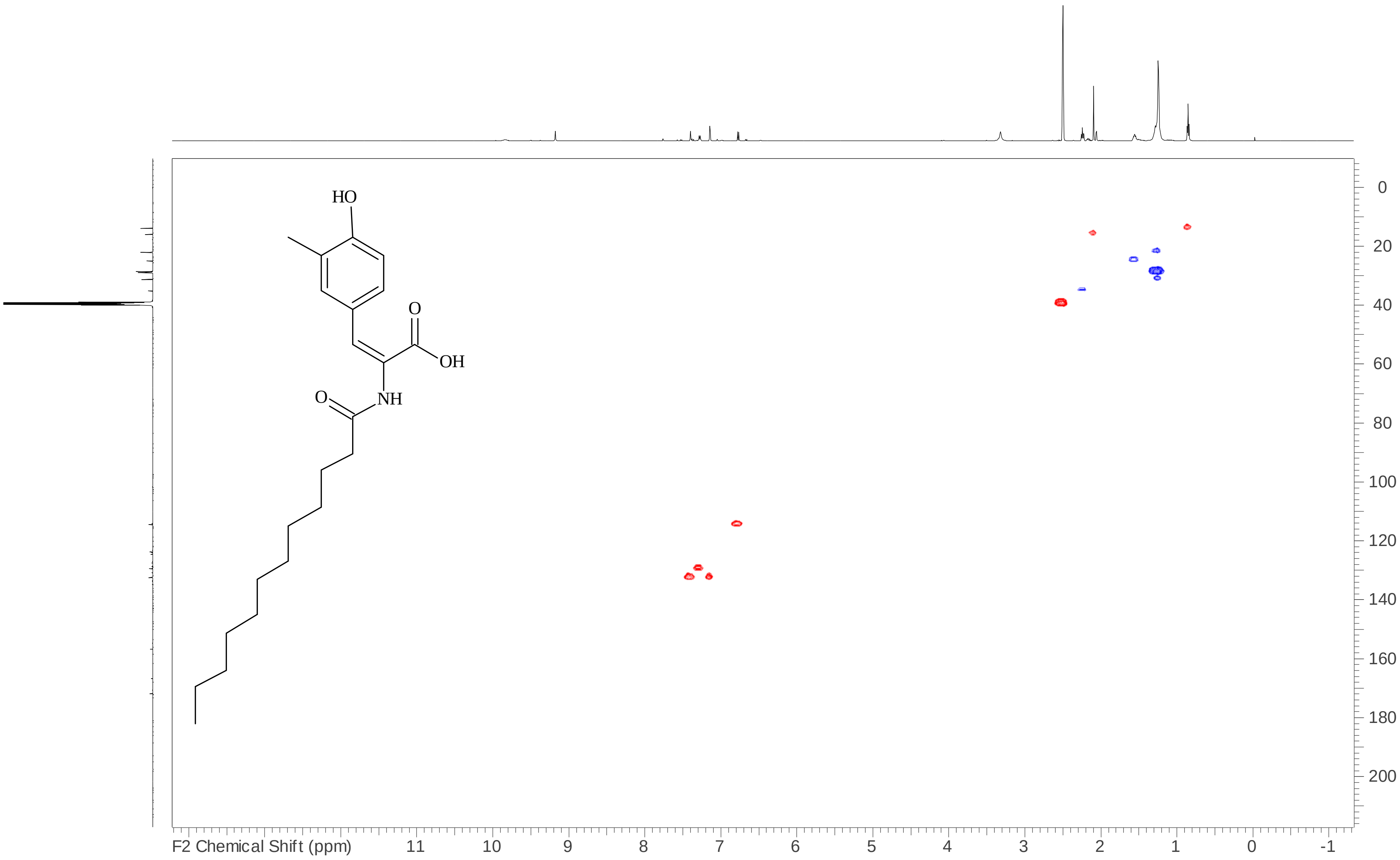

Supplementary Fig. 45. HMBC NMR spectrum (500 MHz, DMSO-d_6) of stieleriacine B_2 (5)

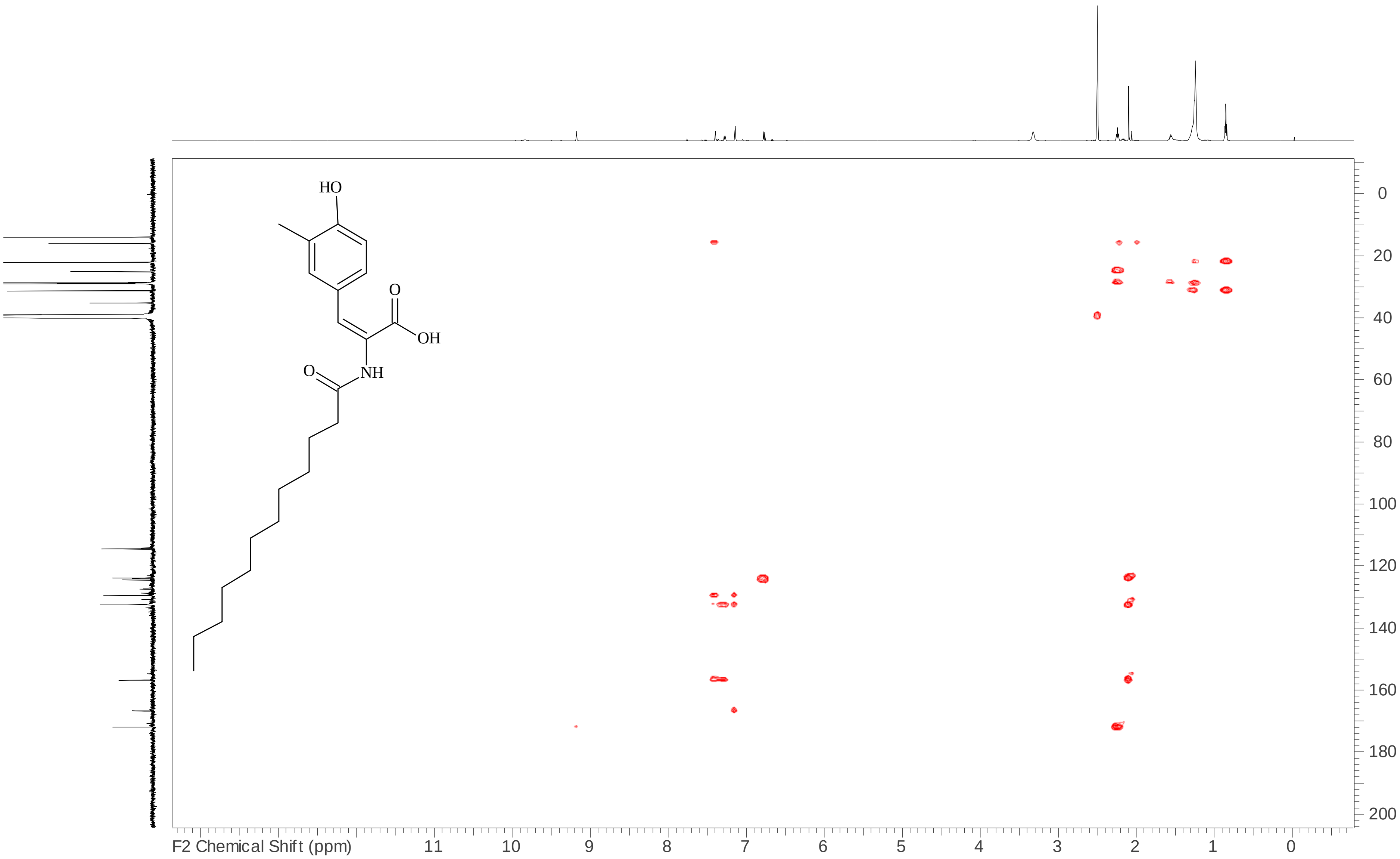

Supplementary Fig. 46. COSY NMR spectrum (500 MHz, DMSO-d_6) of stieleriacine B_2 (5)

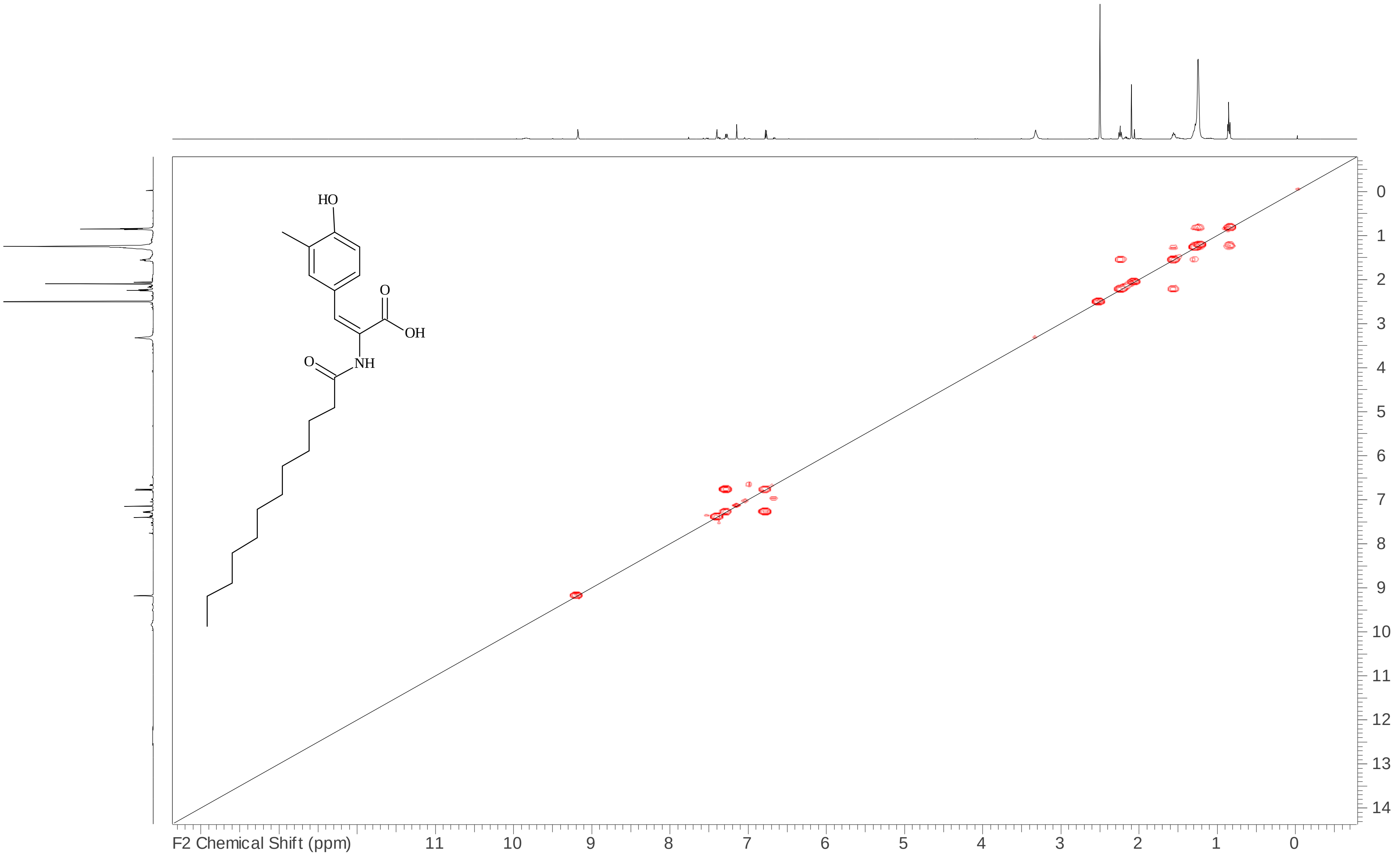

Supplementary Fig. 47. TOCSY NMR spectrum (500 MHz, DMSO-d_6) of stieleriacine B_2 (5)

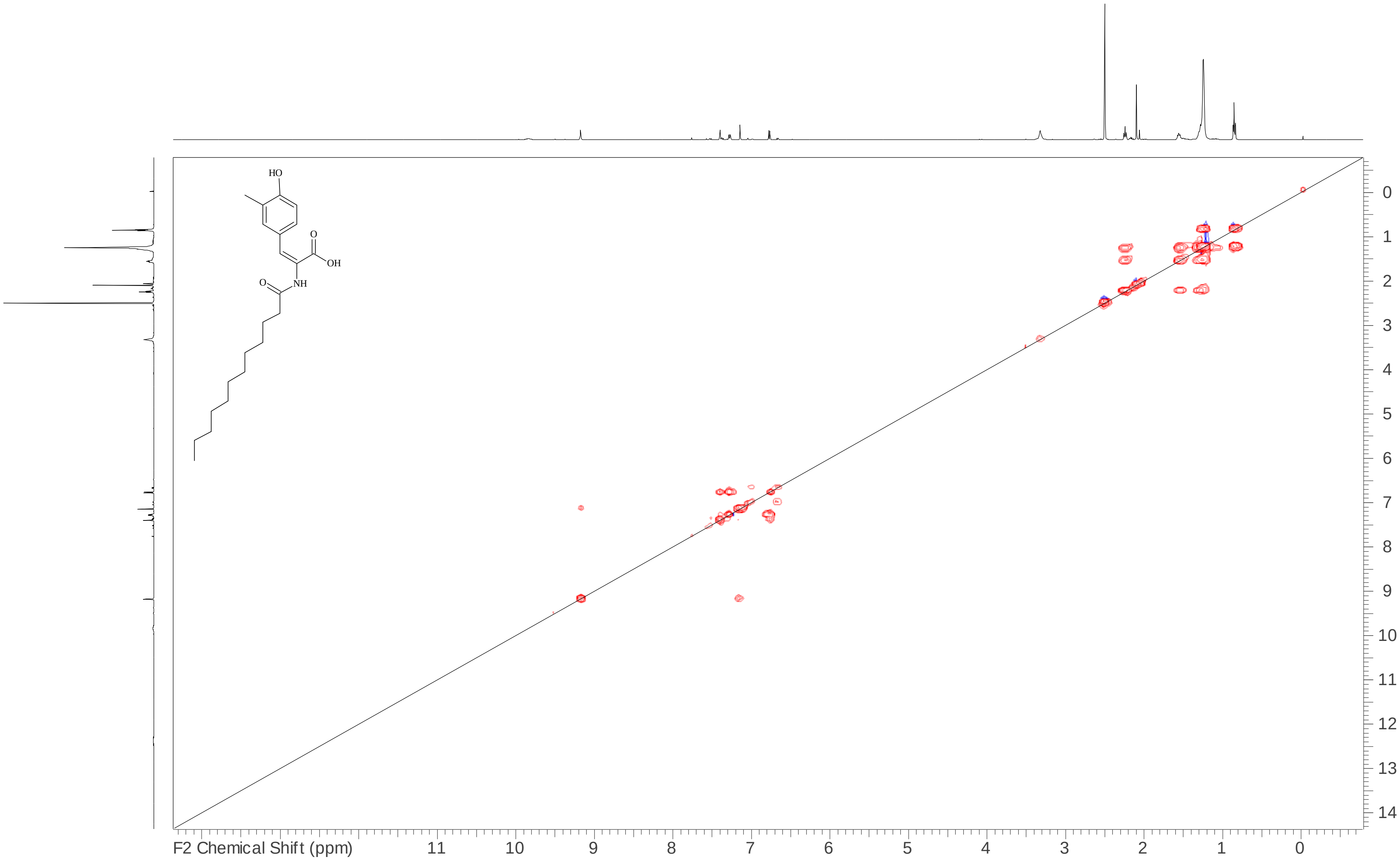

Supplementary Fig. 48. ROESY NMR spectrum (500 MHz, DMSO-d_6) of stieleriacine B_2 (5)

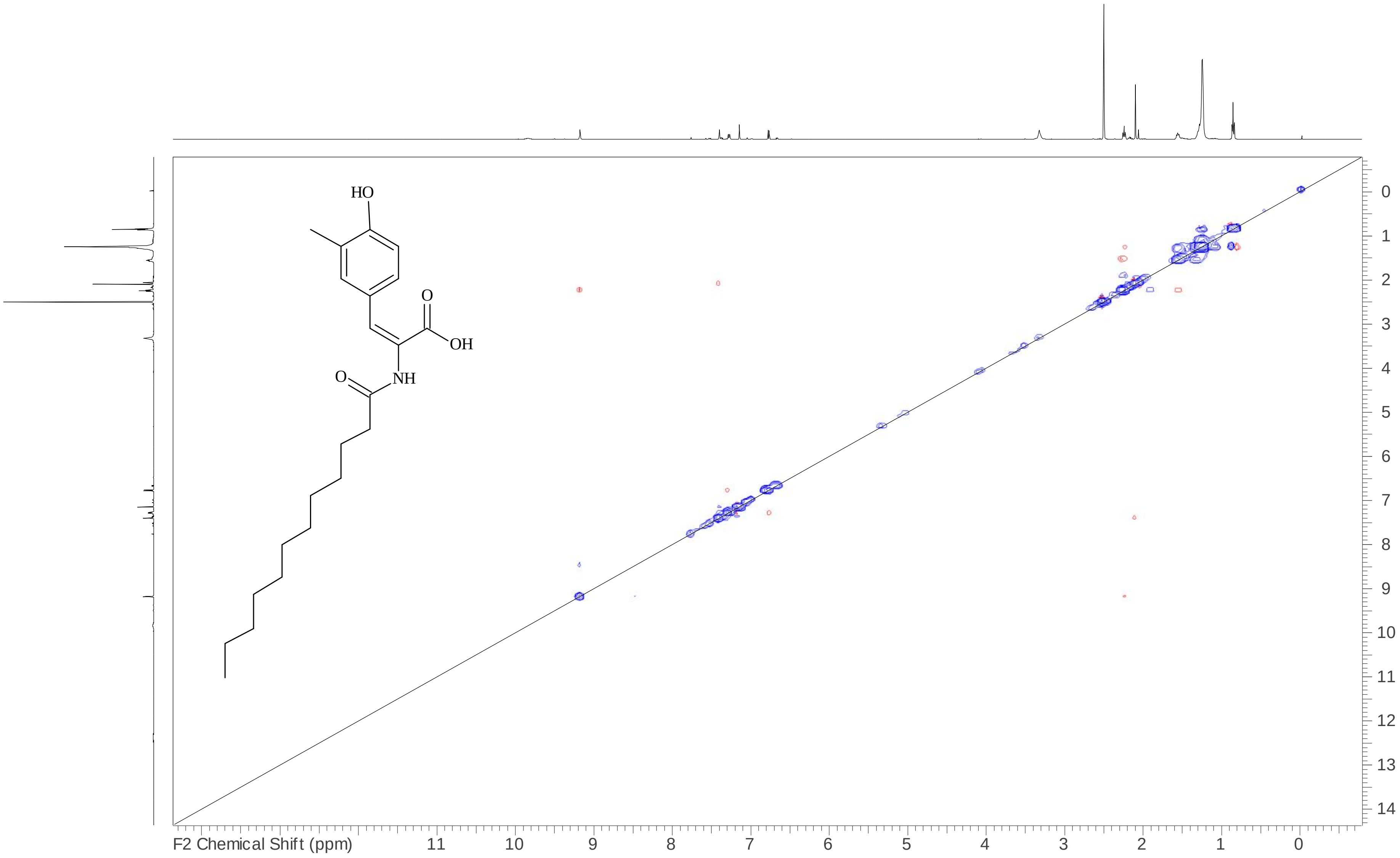

Supplementary Tables

Supplementary Table 1. Minimum inhibitory concentration (MIC) for bacteria, yeasts and fungi in µg/mL. For MICs, a total of 2 µL and 20 µL of a 1 mg/mL stock solution (6.7 and 67 µg/ml) of **1** – **5** were tested. MeOH (20 µL) was tested as negative control and displayed no inhibitory effects. The following reference antimicrobials were used: [a] Oxytetracycline, [b] Gentamycin, [c] Kanamycin for bacteria; [e] Nystatin for yeasts and fungi; n. i. = no inhibition up to 100 µg/mL.

**Strain**	Stieleriacine A_1 (**1**)	Stieleriacine B_1 (**2**)	Stieleriacine C (**3**)	Stieleriacine A_2 (**4**)	Stieleriacine B_2 (**5**)	Reference [a,b,c,d]
Bacillus subtilis DSM 10	50	67	67	67	67	1.1[a]
Escherichia coli DSM 1116	n.i.	n.i.	n.i.	n.i.	n.i.	3.4[a]
Mucor plumbeus MUCL49355	n.i.	n.i.	50	n.i.	n.i.	4.2[e]
Candida tenuis MUCL29892	n.i.	n.i.	n.i.	n.i.	n.i.	6.8[e]
Candida albicans DSM1665	n.i.	n.i.	n.i.	n.i.	n.i.	34[e]
Pichia anomala DSM 6766	n.i.	n.i.	n.i.	n.i.	n.i.	8.4[e]
Micrococcus luteus DSM 1790	n.i.	50	n.i.	50	100	0.8[a]
Chromobacterium violaceum DSM 30191	n.i.	n.i.	n.i.	n.i.	n.i.	1.7[a]
Pseudomonas aeroginosa PA14	n.i.	n.i.	n.i.	n.i.	n.i.	0.1[b]
Mycobacterium smegmatis ATCC 700084	n.i.	n.i.	n.i.	n.i.	n.i.	0.1[c]
Schizosaccharomyces pombe DSM 70572	n.i.	n.i.	n.i.	n.i.	n.i.	8.4[e]
Staphylococcus aureus DSM 346	n.i.	n.i.	67	67	67	0.4[a]
Rhodotorula glutinis DSM 10134	67	n.i.	n.i.	n.i.	n.i.	2.1[e]
Mucor hiemalis DSM 2656	67	n.i.	n.i.	n.i.	n.i.	17[e]

Supplementary Table 2. Duration of T_0, T_1, T_2 and T_3 for untreated Mal15^T cells and cells treated with 1.34 µM stieleriacine A_1. T_0 = duration of lag phase; T_1 = duration of daughter cell formation until release, T_2 = duration of the gap after daughter cell release (T_1) until next daughter cell formation and T_3 = complete reproduction cycle (T_1+T_2). Experiments were performed in three biological replicates.

Replicate	Treatment	T_0[min]	T_1[min]	T_2[min]	T_3[min]	T_3[h]
1	-	262.22	122.96	128.15	251.11	4.19
2		485.56	113.33	142.22	255.56	4.26
3		378.89	111.85	127.78	239.63	3.99
	Ø	375.56	116.05	132.72	248.77	4.15
	deviation	91.21	4.93	6.72	6.71	0.11
1	A_1	201.11	124.07	125.19	249.26	4.15
2		410.00	107.04	153.33	260.37	4.34
3		318.89	116.67	134.07	250.74	4.18
	Ø	310.00	115.93	137.53	253.46	4.22
	deviation	85.51	6.98	11.75	4.93	0.08

Supplementary Table 3. Gene annotation of the putative stieleriacine biosynthetic gene cluster (Mal15_37240 to Mal15_37430) and the region upstream (Mal15_36520 to Mal15_37230).

Locus tag	Automated gene annotation
Mal15_36520	Tetraacyldisaccharide 4'-kinase
Mal15_36530	Iron-responsive transcriptional regulator
Mal15_36540	Hypothetical protein
Mal15_36550	Hypothetical protein
Mal15_36560	Hypothetical protein
Mal15_36570	Serine-aspartate repeat-containing protein D precursor
Mal15_36580	WD domain, G-beta repeat
Mal15_36590	Hypothetical protein
Mal15_36600	Transposase IS200 like protein
Mal15_36610	Transposase for transposon Tn5
Mal15_36620	Transposase for transposon Tn5
Mal15_36630	Chromosome partition protein Smc
Mal15_36640	Hypothetical protein
Mal15_36650	Biopolymer transport protein ExbB
Mal15_36660	Biopolymer transport protein ExbD
Mal15_36670	Hypothetical protein
Mal15_36680	Hypothetical protein
Mal15_36690	FemAB family protein
Mal15_36700	Hypothetical protein
Mal15_36710	Hypothetical protein
Mal15_36720	Transmembrane exosortase (Exosortase_EpsH)
Mal15_36730	Tyrosine-protein kinase YwqD
Mal15_36740	Tetratricopeptide repeat protein
Mal15_36750	Hypothetical protein
Mal15_36760	UDP-*N*-acetylgalactosamine-undecaprenyl-phosphate *N*-acetylgalactosamine-phosphotransferase
Mal15_36770	Peptidoglycan-*N*-acetylglucosamine deacetylase
Mal15_36780	D-inositol-3-phosphate glycosyltransferase
Mal15_36790	Putative teichuronic acid biosynthesis glycosyltransferase TuaC
Mal15_36800	Phosphotransferase enzyme family protein
Mal15_36810	Sulfotransferase domain protein
Mal15_36820	Hypothetical protein
Mal15_36830	Poly-beta-1,6-*N*-acetyl-D-glucosamine *N*-deacetylase precursor
Mal15_36840	Heparinase II/III-like protein
Mal15_36850	Putative teichuronic acid biosynthesis glycosyltransferase TuaC
Mal15_36860	Hypothetical protein
Mal15_36870	Hypothetical protein
Mal15_36880	GDP-mannose 6-dehydrogenase
Mal15_36890	Low molecular weight protein-tyrosine-phosphatase etp
Mal15_36900	Glycosyl transferases group 1
Mal15_36910	Bifunctional polymyxin resistance protein ArnA
Mal15_36920	Hypothetical protein
Mal15_36930	Hypothetical protein
Mal15_36940	Peptidoglycan *O*-acetyltransferase

Locus tag	Automated gene annotation
Mal15_36950	Long-chain-fatty-acid--CoA ligase
Mal15_36960	D-alanine--poly(phosphoribitol) ligase subunit 2
Mal15_36970	L-glutamyl-[BtrI acyl-carrier protein] decarboxylase
Mal15_36980	Hypothetical protein
Mal15_36990	Hypothetical protein
Mal15_37000	Putative glycosyltransferase EpsH
Mal15_37010	Putative oxidoreductase YcjS
Mal15_37020	Hypothetical protein
Mal15_37030	*O*-Antigen ligase
Mal15_37040	Undecaprenyl-phosphate 4-deoxy-4-formamido-L-arabinose transferase
Mal15_37050	Hypothetical protein
Mal15_37060	Teichoic acid translocation permease protein TagG
Mal15_37070	Teichoic acids export ATP-binding protein TagH
Mal15_37080	3-deoxy-manno-octulosonate cytidylyltransferase
Mal15_37090	*N*,*N*'-diacetyllegionaminic acid synthase
Mal15_37100	Putative glycosyltransferase EpsE
Mal15_37110	Hyaluronan synthase
Mal15_37120	Hypothetical protein
Mal15_37130	Hypothetical protein
Mal15_37140	Hypothetical protein
Mal15_37150	tRNA (mo5U34)-methyltransferase
Mal15_37160	bifunctional 3-demethylubiquinone-9 3-methyltransferase/ 2-octaprenyl-6-hydroxy phenol methylase
Mal15_37170	Hemolysin, chromosomal
Mal15_37180	Kynurenine formamidase
Mal15_37190	Beta-monoglucosyldiacylglycerol synthase
Mal15_37200	Poly-beta-1,6-*N*-acetyl-D-glucosamine synthase
Mal15_37210	hypothetical protein
Mal15_37220	*O*-Antigen ligase
Mal15_37230	*O*-acetyltransferase OatA
Mal15_37240	Fatty acid desaturase
Mal15_37250	Carminomycin 4-*O*-methyltransferase
Mal15_37260	tRNA threonylcarbamoyladenosine dehydratase
Mal15_37270	Serine-aspartate repeat-containing protein D precursor
Mal15_37280	Serine-aspartate repeat-containing protein D precursor
Mal15_37290	Hypothetical protein
Mal15_37300	PEP-CTERM motif protein
Mal15_37310	Hypothetical protein
Mal15_37320	Hypothetical protein
Mal15_37330	3-oxoacyl-[acyl-carrier-protein] synthase 2
Mal15_37340	Hypothetical protein
Mal15_37350	Hypothetical protein
Mal15_37360	Transcriptional regulatory protein DegU
Mal15_37370	Hypothetical protein
Mal15_37380	Hypothetical protein
Mal15_37390	Putative efflux pump membrane fusion protein
Mal15_37400	Cobalt-zinc-cadmium resistance protein CzcA

Locus tag	Automated gene annotation
Mal15_37410	Putative efflux pump membrane fusion protein
Mal15_37420	Cobalt-zinc-cadmium resistance protein CzcA
Mal15_37430	Hypothetical protein

Supplementary Table 4. Carbon sources utilised by strain Mal15^T.
+=positive; W=weakly positive

Carbon sources	
Acetic acid	W
N-Acetyl-D-galactosamine	+
N-Acetyl-D-glucosamine	+
L-Arabinose	+
D-Cellobiose	+
Dextrin	W
L-Fucose	+
D-Fructose	+
D-Galactose	+
D-Galactonic acid lacton	W
Gentiobiose	+
α-D-Glucose	+
D-Glucose- 6-phosphate	W
D-Gluconic acid	+
Glucuronamide	+
D-Glucuronic acid	+
α-Ketoglutaric acid	W
α-D-Lactose	+
Lactulose	+
Maltose	W
D-Mannose	+
D-Mannitol	W
D-Melibiose	+
β-Methyl-D-Glucoside	+
D-Raffinose	+
L-Rhamnose	+
Sucrose	+
D-Trehalose	+
Turanose	+
D-Psicose	+

Supplementary Table 5. Enzymatic activities of strain Mal15^T, determined with APIZYM test. Results: + positive; – negative

**Enzymatic activity**	
Alkaline phosphatase	+
Esterase (C4)	+
Esterase lipase (C8)	+
Lipase (C14)	–
Leucine arylamidase	+
Valine arylamidase	+
Cysteine arylamidase	–
Trypsin	–
α-Chymotrypsin	–
Acid phosphatase	+
Naphthol-AS-BI-phosphohydrolase	+
α-Galactosidase	–
β-Galactosidase	–
β-Glucuronidase	–
α-Glucosidase	–
β-Glucosidase	–
N-Acetyl-β-glucosaminidase	–
α-Mannosidase	–
α-Fucosidase	–

Supplementary Notes

Phylogenetic inference

In phylogenetic trees obtained after 16S rRNA gene sequence comparison and whole genome-based multilocus sequence analysis (MLSA), strain Mal15^T clusters within the family *Pirellulaceae*, order *Pirellulales*, class *Planctomycetia*, phylum *Planctomycetes* (Supplementary Fig. 2). Within its family, strain Mal15^T clusters stably with the genera of *Rhodopirellula*, *Rubripirellula*, *Novipirellula* and *Crateriforma* without revealing any current genus as current closest neighbour. Analysis of 16S rRNA gene sequence similarity (Supplementary Fig. 3) shows that all minimal identities between strain Mal15^T and the four other genera are below the threshold of 94.5% that would put strain Mal15^T in any of these taxa[1]. The same is true for the analysis of *rpoB* nucleotide sequences identity (genus threshold of 75.5-78.0%)[2], average amino acid identity (genus threshold of 60-80%)[3] and percentage of conserved proteins (genus threshold of 50%)[4] (Supplementary Fig. 3). The only exception was observed during comparison of strain Mal15^T with the genus *Crateriforma*, for which the values of *rpoB* nucleotide sequences identities and percentage of conserved proteins are slightly above the threshold. Nevertheless, strain Mal15^T and *Crateriforma conspicua* do not cluster monophyletically in any phylogenetic tree (Supplementary Fig. 2) and it is therefore unlikely that they belong to the same genus. Taken together, the different methods suggest that strain Mal15^T represents a novel species within a novel genus in the family *Pirellulaceae*, for which we propose the name *Stieleria maiorica* gen. nov., sp. nov. Strain Mal15^T (DSM 100215^T = LMG 29790^T) is the type strain of the novel species and *Stieleria maiorica* is introduced as type species of the novel genus.

Morphological Characterisation

Cells of strain Mal15^T were investigated using light microscopic and electron microscopic analysis (Supplementary Fig. 4), revealing round to pear-shaped cells with a smooth surface, which form chains or rosettes. Cells are 1.9±0.2 x 1.4±0.2 µm in size (Supplementary Fig. 4).

Colonies appear smooth, round and are pink-coloured on solid medium. Cells are motile swimmers in liquid cultures. When strain Mal15^T is grown under constant agitation (80 rpm), stirring or highly aerated, cells produce an extracellular matrix. Strain Mal15^T divides by polar budding.

Physiological Characterisation

Strain Mal15^T is an aerobic heterotroph. Compared to other planctomycetes characterised so far (typical μ_{max} of 0.01-0.08 h^{-1}), strain Mal15^T is fast-growing. It reaches a maximal growth rate of 0.093 h^{-1} (generation time of 7.5 h) (Supplementary Fig. 5). Determination of the growth rates at different temperatures revealed a mesophilic growth profile with growth from 11 °C to 37 °C with 35 °C as optimal growth temperature (Supplementary Fig. 5a). Measurements at different pH at 35 °C revealed growth in the range of pH 5.5-9.0 with an optimum at pH 7.5 (Supplementary Fig. 5b). Strain Mal15^T was found to be oxidase-negative and catalase-positive. A conventional Gram stain was not possible for the strain as described previously for planctomycetes[5]. The Biolog GN2 MicroLog test showed that strain Mal15^T is capable to utilise a variety of carbon sources (Supplementary Table 4). In particular sugars, such as *N*-acetyl-D-galactosamine, *N*-acetyl-D-glucosamine, L-arabinose, D-cellobiose, L-fucose, D-fructose, D-galactose, gentiobiose, α-D-glucose, D-gluconic acid, glucuronamide, D-glucuronic acid, α-D-lactose, lactulose, D-mannose, D-melibiose, β-methyl- D-glucoside, D-raffinose, L-rhamnose, sucrose, D-trehalose, turanose and D-psicose appear to be preferred carbon sources. Acetic acid, dextrin, D-galactonic acid lactone, D-glucose-6-phosphate, α-ketoglutaric acid, maltose, and D-mannitol showed week reactions, but still over 25% in both biological replicates (Supplementary Table 5). The APIZYM method revealed the following enzyme repertoire: alkaline phosphatase, esterase (C4), esterase lipase (C8), leucine arylamidase, valine arylamidase, acid phosphatase and naphthol-AS-BI-phosphohydrolase (Supplementary Table 6).

Genome Analysis

The complete chromosome of strain Mal15^T comprises 9,894,293 bp and has a G+C content of 59.3%. Annotation with Prokka revealed 7,016 annotated genes in its genome, of which 6,920 were identified as putative protein-coding genes. As known for other planctomycetes[6], only for 42% of these genes a function could be predicted, while the remaining 2,899 genes were annotated as hypothetical proteins or proteins with unknown function. Moreover, 7 rRNA (3x 16S, 2x 23S, 2x 5S) and 81 tRNA entries were annotated. The genomic features of strain Mal15^T are summarised in Supplementary Fig. 6. As planctomycetes are presumed to be valuable sources for the discovery of novel bioactive secondary metabolites[7-9], the genome of Mal15^T was scanned thereupon using antiSMASH version 3.0.5[10]. In total, seven secondary metabolite-associated gene clusters were identified. Like most planctomycetes, the genome of Mal15^T harbors one terpene-related cluster[7,8,11]. The remaining six clusters are related to thiotemplate modular systems (TMS) including nonribosomal peptide synthetases (NRPS) and polyketide synthases (PKS). Interestingly, one of these clusters represents a hybrid TMS cluster related to NRPS and PKS. TMS clusters are known to be associated with the production of numerous small bioactive molecules of medical importance[12]. However, most secondary metabolite-related genes or clusters are known to be silent under axenic laboratory cultivation conditions[13-15], which can be also assumed for planctomycetal secondary metabolite clusters[7].

Description of *Stieleria* gen. nov.

Stieleria (*Stie.le'ri.a'* N.L. fem. n. *Stieleria* named in honor of Anja Heuer, née Stieler, an extraordinary skilled German technician at the Leibniz Institute DSMZ, who played a key role in the cultivation of literally hundreds of novel planctomycetal strains). The round to pear-shaped cells with a smooth cell surface form rosettes or short chains. Cells reproduce by polar budding. In liquid culture, they produce an extracellular matrix which interconnects cells in aggregates. Daughter cells are motile, whereas mother cells are non-motile. The lifestyle is heterotrophic, obligatory aerobic, mesophilic and neutrophilic. The genus belongs to the

phylum *Planctomycetes,* class *Planctomycetia*, order *Pirellulales*, family *Pirellulaceae.* The type species is *Stieleria maiorica*.

Description of *Stieleria maiorica* sp. nov.

Stieleria maiorica (ma'i.ori.ca' N.L. fem. adj. *maiorica*, pertaining to the island Mallorca, Spain, on which the type strain was isolated). In addition to the features described above the species exhibits the following properties: Colonies are pink-coloured on solid medium. Cells are 1.9±0.2 x 1.4±0.2 µm in size. Motile daughter cells originate through budding from sessile mother cells. Gram staining delivers no clear results. The oxidase assay was negative while the catalase assay was positive. The organism can degrade a wide range of carbon sources. In particular, strong signals were observed for *N*-acetyl-D-galactosamine, *N*-acetyl-D-glucosamine, L-arabinose, D-cellobiose, L-fucose, D-fructose, D-galactose, gentiobiose, α-D-glucose, D-gluconic acid, glucuronamide, D-glucuronic acid, α-D-lactose, lactulose, D-mannose, D-melibiose, β-methyl-D-glucoside, D-raffinose, L-rhamnose, sucrose, D-trehalose, turanose and D-psicose while for the carbon sources acetic acid, dextrin, D-galactonic acid lactone, D-glucose-6-phosphate, α-ketoglutaric acid, maltose, D-mannitol only weak signals were detected. The enzyme repertoire includes alkaline phosphatase, esterase (C4), esterase lipase (C8), leucine arylamidase, valine arylamidase, acid phosphatase and naphthol-AS-BI-phosphohydrolase. Growth of the type strain occurs between pH 5.5 and 9.0 with an optimum at pH 7.5. The optimal growth temperature of the type strain turned out to be 35 °C, but cells also grew over a range from 11 °C to 37 °C. The complete chromosome of the type strain comprises 9,894,293 bp, with a G+C content of 59.3%.The type strain is Mal15^T (DSM 100215^T = LMG 29790^T), which was isolated from seawater sediment on Mallorca island, Spain.

Compound information for Stieleriacines

Stieleriacine A_1 (1) (Supplementary Figs. 9-16). (*Z* isomer); off-white, amorphous powder; UV (MeOH) λ_{max} (log ε) 200 (2.6), 314 (2.2) nm. HRESIMS m/z 374.2325 $[M+H]^+$ (calcd for

$C_{22}H_{31}NO_4$, 373.2253). ^{1}H NMR (700MHz, DMSO-d_6) δ = 9.82 (1H, s, C-OH-7), 9.29 (1H, s, NH), 7.35 (1H, d, J = 1.7 Hz, H-9), 7.28 (1H, dd, J = 8.4 Hz, 1.9 Hz, H-5), 7.17 (1H, s, H-3), 6.77 (1H, d, J = 8.4 Hz, H-6), 6.69 (1H, dt, J = 15.4 Hz, 6.9 Hz, H-3'), 6.11 (1H, d, J = 15.5 Hz, H-2'), 2.18 (2H, q, J = 1.0 Hz, H-4'), 2.08 (3H, m, H-10), 1.43 (2H, tquin, J = 7.3 Hz, 1.0 Hz, H-5'), 1.26 (14H, m, H-6', 7', 8', 9', 10', 11'), 0.85 (3H, dt, J = 14.6 Hz, 7.1 Hz, H-12'). ^{13}C NMR (176MHz, DMSO-d_6) δ = 166.6 (C-1), 164.4 (C-1'), 156.9 (C-7), 144.2 (C-3'), 132.7 (C-9), 132.6 (C-3), 129.3 (C-5), 124.5 (C-4), 123.9 (C-8), 123.8 (C-2'), 123.6 (C-2), 114.6 (C-6), 31.3 (C-4'), 31.3 (C-10'), 29.0 - 28.6 (C-6', 7', 8', 9'), 27.8 (C-5'), 22.1 (C-11'), 16.0 (C-10), 14.0 (C-12').

Stieleriacine B_1 (2) (Supplementary Figs. 17-24). (*Z* isomer); off-white, amorphous powder; UV (MeOH) λ_{max} (log ε) 201 (1.5), 227 (1.4), 310 (1.6) nm. HRESIMS m/z 376.2490 $[M+H]^+$ (calcd for $C_{22}H_{33}NO_4$, 375.2409). ^{1}H NMR (500MHz, DMSO-d_6) δ = 9.13 (1H, br s, NH), 7.37 (1H, s, H-9), 7.25 (1H, d, J = 7.6 Hz, H-5), 7.12 (1H, s, H-3), 6.76 (1H, d, J = 8.2 Hz, H-6), 2.24 (2H, t, J = 7.3 Hz, H-2'), 2.09 (3H, s, H-10), 1.55 (2H, m, H-3'), 1.26 (16H, m, H-4', 5', 6', 7', 8', 9', 10', 11'), 0.86 (3H, t, J = 7.2 Hz, H-12'). ^{13}C NMR (126MHz, DMSO-d_6) δ = 171.7 (C-1'), 167.0 (C-1), 156.6 (C-7), 132.4 (C-9), 131.7 (C-3), 129.4 (C-5), 124.8 (C-4), 123.7 (C-8, 2), 114.5 (C-6), 35.3 (C-2'), 31.3 (C-10'), 29.0 - 28.7 (C-4', 5', 6', 7', 8', 9'), 25.1 (C-3'), 22.1 (C-11'), 16.0 (C-10), 13.9 (C-12').

Stieleriacine C (3) (Supplementary Figs. 25-32). Light yellow amorphous solid; $[\alpha]_D^{21}$ -28 (c = 1, CH_3OH); UV (MeOH) λ_{max} (log ε) 202 (1.8), 227 (1.3), 279 (0.8), 312 (0.2) nm. HRESIMS m/z 378.2653 $[M+H]^+$ (calcd for $C_{22}H_{35}NO_4$, 377.2566). ^{1}H NMR (700MHz, DMSO-d_6) δ = 7.86 (1H, br s, NH), 6.86 (1H, s, H-9), 6.79 (1H, dd, J = 8.2 Hz, 1.8 Hz, H-5), 6.62 (1H, d, J = 8.0 Hz, H-6), 4.26 (1H, m, H-2), 2.87 (1H, dd, J = 13.8 Hz, 5.0 Hz, H-3a), 2.68 (1H, dd, J = 13.8 Hz, 9.1 Hz, H-3b), 2.05 (3H, s, H-10), 2.02 (2H, td, J = 7.3 Hz, 2.5 Hz, H-2'), 1.39 (2H, m, H-3'), 1.22 (14H, m, H-5', 6', 7', 8', 9', 10', 11'), 1.14 (2H, m, H-4'), 0.85 (3H, t, J = 7.0 Hz, H-12'). ^{13}C NMR (176MHz, DMSO-d_6) δ = 173.3 (C-1), 171.9 (C-1'), 153.8 (C-7),

131.3 (C-9), 127.8 (C-4), 127.1 (C-5), 123.1 (C-8), 114.2 (C-6), 53.9 (C-2), 36.1 (C-3), 35.2 (C-2'), 31.3 (C-10'), 29.0 - 28.7 (C-5', 6', 7', 8', 9'), 28.5 (C-4'), 25.2 (C-3'), 22.1 (C-11'), 16.0 (C-10), 13.9 (C-12')

Stieleriacine A_2 (4) (Supplementary Figs. 33-40). (*E* isomer); off-white, amorphous powder; UV (MeOH) λ_{max} (log ε) 210 (1.6), 314 (1.6) nm. HRESIMS m/z 374.2336 $[M+H]^+$ (calcd for $C_{22}H_{31}NO_4$, 373.2253). ^{1}H NMR (500MHz, DMSO-d_6) δ = 9.28 (1H, s, NH), 7.34 (1H, s, H-9), 7.28 (1H, dd, J = 8.3 Hz, 1.9 Hz, H-5), 7.17 (1H, s, H-3), 6.78 (1H, d, J = 8.4 Hz, H-6), 6.69 (1H, m, H-3'), 6.12 (1H, d, J = 15.4 Hz, H-2'), 2.18 (2H, m, H-4'), 2.09 (3H, s, H-10), 1.44 (2H, m, H-5'), 1.28 (14H, m, H-5', 6', 7', 8', 9', 10', 11'), 0.87 (3H, t, J = 6.6 Hz, H-12'). ^{13}C NMR (126MHz, DMSO-d_6) δ = 166.7 (C-1), 164.3 (C-1'), 156.8 (C-7), 144.1 (C-3'), 132.7 (C-9), 132.2 (C-3), 129.2 (C-5), 124.6 (C-4), 123.8 (C-8, 2, 2'), 114.6 (C-6), 31.3 (C-4'), 31.3 (C-10'), 29.0 - 28.6 (C-6', 7', 8', 9'), 27.8 (C-5'), 22.1 (C-11'), 16.0 (C-10), 13.9 (C-12').

Stieleriacine B_2 (5) (Supplementary Figs. 41-48). (*E* isomer); off-white, amorphous powder; UV (MeOH) λ_{max} (log ε) 202 (1.6), 310 (1.5) nm. HRESIMS m/z 376.2498 $[M+H]^+$ (calcd for $C_{22}H_{33}NO_4$, 375.2409). ^{1}H NMR (500MHz, DMSO-d_6) δ = 9.18 (1H, s, NH), 7.40 (1H, m, H-9), 7.28 (1H, dd, J = 8.3 Hz, 1.4 Hz, H-5), 7.14 (1H, s, H-3), 6.77 (1H, d, J = 8.4 Hz, H-6), 2.24 (2H, t, J = 7.3 Hz, H-2'), 2.10 (3H, s, H-10), 1.56 (2H, m, H-3'), 1.20 - 1.32 (16H, m, H-4', 5', 6', 7', 8', 9', 10', 11'), 0.85 (3H, t, J = 6.9 Hz, H-12'). ^{13}C NMR (126MHz, DMSO-d_6) δ = 171.9 (C-1'), 166.8 (C-1), 156.8 (C-7), 132.5 (C-9), 132.4 (C-3), 129.5 (C-5), 124.5 (C-4), 124.0 (C-8), 123.8 (C-2), 114.5 (C-6), 35.2 (C-2'), 31.3 (C-10'), 29.0 - 28.6 (C-4', 5', 6', 7', 8', 9'), 25.1 (C-3'), 22.1 (C-11'), 16.0 (C-10), 13.9 (C-12').

Structure Elucidation

The analysis of the culture broth of strain $Mal15^T$ for potential bioactive secondary metabolites led to the isolation of five previously undescribed compounds (Supplementary Figs. 7 and 8). Stieleriacine A_1 (**1**) was isolated as an off-white, amorphous solid. Its

molecular formula was determined by HRESIMS as $C_{22}H_{31}NO_4$, implying eight degrees of unsaturation. The ^1H NMR exhibited signals of three aromatic protons (δ 6.77, 7.28, 7.35), three methines (δ 6.11, 6.69, 7.17), a singlet for a methyl group (δ 2.08), methylene group proton signals (δ 1.26, 1.43, 2.18) and a triplet for a methyl group (δ 0.85). Singlets at δ 9.29 and 9.82 were attributed to an NH and OH group, respectively. The ^{13}C and distortionless enhancement by polarisation transfer (DEPT) spectra shows 22 carbon signals, being one carboxy, one carbonyl, three aromatic methines, three aromatic quaternary carbons, two methyls, eight methylenes, three methines, and one quaternary sp^3 hybridised carbon. $^1H,^1H$ correlated spectroscopy (COSY) and $^1H,^{13}C$ heteronuclear multiple bond correlation (HMBC) spectra revealed a dodecenoyl moiety with the double bond in *E* configuration between C-2’ and C-3’ attached via an amide bond to *meta*-methyl-dehydrotyrosine (Supplementary Fig. 8). ROE correlations support the double bond between C-2 and C-3 to be in *Z* configuration (Supplementary Fig. 8). The NMR data were similar compared to the previously reported thalassotalic acids[16] with differences in the length of the side chain moiety, a methyl group in *meta*-position of the aromatic ring, and the degree of unsaturation of the latter.

Stieleriacine B_1 (**2**), an off-white amorphous powder, was identified as $C_{22}H_{33}NO_4$ by HRESIMS, indicating seven degrees of unsaturation. The major difference in the NMR data of **2** compared to **1** was the absence of the doublet (δ 6.11) and the doublet of triplets (δ 6.69) in the ^1H NMR, as well as the corresponding carbon atoms at 123.8 and 144.2 ppm, respectively. Instead a new multiplet (δ 1.55) appears corresponding to a methylene carbon signal (δ 35.3). The integration of the peak at 1.26 ppm corresponding to methylenes in the heteronuclear single quantum coherence (HSQC)-dept also shows two more protons, confirming the absence of the double bond in the side chain moiety. The carbon shift of C-3 (δ 131.7) could be retrieved from HSQC data. The shift of carbon C-2 remains unclear. Measuring of compound **2** in a different solvent, like acetone-d_6, did not bring clarity to the exact position. Likewise, to compound **1**, C-2 is also not showing any HMBC correlations. However, comparison to compounds **1** and **5** allows the suggestion that the shift of C-2 is overlapping with C-8 (δ 123.7).

Stieleriacine C (**3**), a light yellow amorphous solid, has a molecular formula of $C_{22}H_{35}NO_4$ as determined by HRESIMS, suggesting six degrees of unsaturation. The only noticeable difference to compound **2** in the NMR data is the appearance of a multiplet (δ 4.26) and two doublets of doublets (δ 2.87 and 2.68) in the ^{1}H NMR. The ^{13}C NMR shows a replacement of a methine and a quaternary sp^3 hybridised carbon signal by a methylene (δ 36.1) and a methine (δ 53.9), respectively. HMBC correlations and a negative optical rotation further endorse a *meta*-methylated L-tyrosine moiety.

Stieleriacine A_2 (**4**), an off-white, amorphous solid, was determined by HRESIMS to possess a molecular formula of $C_{22}H_{31}NO_4$, proposing eight degrees of unsaturation (Supplementary Fig. 8). Despite the absence of one carbon atom, the NMR data is fairly consistent with stieleriacine A_1 (**1**), with the only noteworthy difference in the absence of correlations between the NH proton signal with H-5 and H-9 in the rotating frame nuclear Overhauser effect correlation spectroscopy (ROESY) spectra (Supplementary Fig. 8). This difference affirms the double bond between C-2 and C-3 of compound **4** to be in *E* configuration.

Stieleriacine B_2 (**5**) was isolated as an off-white, amorphous powder and could be assigned a molecular formula of $C_{22}H_{33}NO_4$ by HRESIMS, indicating seven degrees of unsaturation. The NMR data is in accordance with the data set of stieleriacine B_1 (**2**), with the main difference being the missing correlations of the NH proton signal with H-5 and H-9 in the ROESY spectra, which allows the conclusion that the double bond between C-2 and C-3 of compound **5** is in *E* configuration.

Bioactivities of Stieleriacines

All compounds were tested against a variety of bacteria and fungi in an antimicrobial activity assay (Supplementary Table 1). Stieleriacine A_1 (**1**) had weak activity against *Bacillus subtilis* (minimal inhibitory concentration (MIC) 50 µg/mL), *Mucor hiemalis* (MIC 67 µg/mL), and *Rhodotorula glutinis* (MIC 67 µg/mL). Stieleriacine B_1 (**2**) showed weak activity against *B. subtilis* (MIC 67 µg/mL) and *Micrococcus luteus* (MIC 50 µg/mL). Furthermore, stieleriacine C (**3**) also had weak activity against *B. subtilis* (MIC 67 µg/mL), *Mucor plumbeus* (MIC 50

µg/mL) and *Staphylococcus aureus* (MIC 67 µg/mL). Stieleriacine A_2 (**4**) showed weak activity against *B. subtilis* (MIC 67 µg/mL), *M. luteus* (MIC 50 µg/mL) and *S. aureus* (MIC 67 µg/mL). Likewise, stieleriacine B_2 had a weak activity against *B. subtilis* (MIC 67 µg/mL), *M. luteus* (MIC 100 µg/mL) and *S. aureus* (MIC 67 µg/mL). None of the above-mentioned compounds showed signs of cytotoxicity in a proliferation assay against the mouse fibroblast cell line L929 and HeLa KB3.1 cells. Despite their similarity to previously isolated tyrosinase-inhibiting substances[16], stieleriacines B_1 and B_2 did not inhibit mushroom tyrosinase in our experiments. Stieleriacines A_1 and A_2 exhibited weak inhibiting effects (IC_{50} 1.4 mM and 1.9 mM, respectively) compared to the known tyrosinase inhibitors arbutin (IC_{50} 140 µM) and kojic acid (IC_{50} 110 µM). Likewise, stieleriacine C showed weak tyrosinase inhibiting effects (IC_{50} 0.9 mM) (for more details see Supplementary Table 6).

Effect of Stieleriacine A_1 on *Stieleria maiorica*

To analyse the physiological impact of stieleriacine A_1 on Mal15^T cells, the cell cycle was determined by time-lapse microscopy (Fig. 3). The cell cycle was separated into three phases: T_0 = duration of lag-phase; T_1 = duration of daughter cell formation until release, T_2 = duration of the gap after daughter cell release (T_1) until next daughter cell formation and T_3 = complete reproduction cycle ($T_1 + T_2$). No significant difference of T_3 was observed for cells incubated with a physiological concentration (1.34 µM) of stieleriacine A_1. Untreated cells showed a mean duration of T_1 = 116.05 min and T_2 = 132.71 min (T_3 = 248.8 min), while treated cells showed a mean duration of T_1 = 115.93 min and T_2 = 137.53 min (T_3 = 253.5 min). T_0 and T_1 of untreated Mal15^T cells and cells treated with 1.34 µM stieleriacine A_1 are listed in Supplementary Table 2. A significant difference was observed for T_0. Untreated cells displayed a T_0 (p = 0.0001) duration of 375.6 min (6.25 h), whereas cells treated with stieleriacine A_1 required 310.0 min (5.16 h) to start division (Fig. 3). In total, T_0 duration was 65.6 min shorter in the presence of stieleriacine A_1.

Effect of Stieleriacine A_1 on strains of the '*Roseobacter* group'

The standardised biofilm formation assay revealed a significant increase (35%, $p < 0.001$) in biofilm formation of *P. inhibens* DSM 17395 in the presence of 134 µM stieleriacine A_1 (Fig. 4). However, the same treatment of *Sulfitobacter dubius* DSM 16472^T cultures resulted in a 15% ($p < 0.001$) lower capacity of biofilm formation (Fig. 4). Physiological concentrations of stieleriacine A_1 (1.34 µM) did not significantly affect the capacity to form biofilms of both *P. inhibens* and *S. dubius* (Fig. 4a). In a further approach, we tested whether stieleriacine A_1 interacts with the LuxI/LuxR system. Thus, we applied the biofilm formation assay on two independent transposon mutants (Tm#3902, Tm#3163) of the exclusive LuxI/LuxR system (PGA1_c03880)[17] in *P. inhibens* DSM 17395 (Supplementary Fig. 1). Both Δ*luxR* transposon mutants showed almost identical absorbance values for CV at 595 nm in the biofilm formation assay. Thus, they were combined and treated as biological replicates (Fig. 4b). Controls revealed that neither the interruption of the *luxR* coding sequence nor the presence of the transposon itself seem to influence the amount or strength of biofilm formation in the controls. However, results for the Δ*luxR* strains display a significant effect on the biofilm formation capacity when the Δ*luxR* cells were treated with both tested concentrations of stieleriacine A_1 (134 µM, 57.5% ($p < 0.001$), 1.34 µM, 17.8% ($p = 0.05$)). A significant increase of biofilm amount can be observed compared to the controls (Supplementary Fig. 10). Remarkably, the effects on biofilm formation with 134 µM stieleriacine A_1 are even significantly stronger (38.4%) for the Δ*luxR* mutants than for the wild type (Fig. 4).

Supplementary Methods

Conditions for isolation of strain Mal15^T

Seawater sediment samples from 30 cm depth were used for the targeted isolation of novel planctomycetes. In general, the composition of the used medium was inspired by previous work on cultivation of planctomycetes[18,19]. Given that all known members of the phylum *Planctomycetes* show high tolerance against β-lactam antibiotics, probably due to presence of β-lactamases[20], β-lactam antibiotics were used as selection pressure. In addition,

cycloheximide was used as antifungal agent. Obtained colonies of resistant bacteria were screened by 16S rRNA gene sequencing.

Genome information

The genome and 16S rRNA gene sequence of strain Mal15^T are available from GenBank under accession numbers CP036264 and MK554562, respectively.

Identification of transposon insertions within the *luxR* gene of *P. inhibens*

We identified transposon insertions into the *luxR* gene (PGA1_c03880) of *P. inhibens* DSM 17395 employing the length difference observed in gel electrophoresis using primers P1903 (5´-CAGTGGGTTGTGACGCTAGA-3´) and P1904 (5´-AGGACCAGATTACGACCAGA-3´), cf. Supplementary Fig. 1.

Supplementary References

1 Yarza, P. *et al.* Uniting the classification of cultured and uncultured bacteria and archaea using 16S rRNA gene sequences. *Nat. Rev. Microbol.* **12**, 635-645, doi:10.1038/nrmicro3330 (2014).

2 Kallscheuer, N. *et al.* Description of three bacterial strains belonging to the new genus *Novipirellula* gen. nov., reclassificiation of *Rhodopirellula rosea* and *Rhodopirellula caenicola* and readjustment of the genus threshold of the phylogenetic marker *rpoB* for *Planctomycetaceae*. *Antonie Van Leeuwenhoek,* doi: 10.1007/s10482-019-01374-5 (2019).

3 Konstantinidis, K. T. & Tiedje, J. M. Towards a genome-based taxonomy for prokaryotes. *J. Bacteriol.* **187**, 6258-6264 (2005).

4 Qin, Q.-L. *et al.* A proposed genus boundary for the prokaryotes based on genomic insights. *J. Bacteriol.* **196**, 2210-2215 (2014).

5 Kohn, T. *et al. Fuerstia marisgermanicae* gen. nov., sp. nov., an Unusual Member of the Phylum Planctomycetes from the German Wadden Sea. *Front. Microbiol.* **7**, 2079, doi:10.3389/fmicb.2016.02079 (2016).

6 Fuerst, J. A. & Sagulenko, E. Beyond the bacterium: Planctomycetes challenge our concepts of microbial structure and function. *Nat. Rev. Microbiol.* **9**, 403-413, doi:10.1038/nrmicro2578 (2011).

7 Jeske, O., Jogler, M., Petersen, J., Sikorski, J. & Jogler, C. From genome mining to phenotypic microarrays: Planctomycetes as source for novel bioactive molecules. *Antonie Van Leeuwenhoek* **104**, 551-567, doi:10.1007/s10482-013-0007-1 (2013).

8 Graça, A. P., Calisto, R. & Lage, O. M. Planctomycetes as Novel Source of Bioactive Molecules. *Front. Microbiol.* **7**, 1241, doi:10.3389/fmicb.2016.01241 (2016).

9 Challinor, V. L. & Bode, H. B. Bioactive natural products from novel microbial sources. *Ann. NY Acad. Sci.* **1354**, 82-97, doi:10.1111/nyas.12954 (2015).

10 Weber, T. *et al.* antiSMASH 3.0-a comprehensive resource for the genome mining of biosynthetic gene clusters. *Nucleic Acids Res.* **43**, W237-243, doi:10.1093/nar/gkv437 (2015).

11 Aghnatios, R. *et al.* Draft genome of *Gemmata massiliana* sp. nov, a water-borne *Planctomycetes* species exhibiting two variants. *Stand. Genomic Sci.* **10**, 120, doi:10.1186/s40793-015-0103-0 (2015).

12 Donadio, S., Monciardini, P. & Sosio, M. Polyketide synthases and nonribosomal peptide synthetases: the emerging view from bacterial genomics. *Nat. Prod. Rep.* **24**, 1073-1109, doi:10.1039/b514050c (2007).

13 van Wezel, G. P. & McDowall, K. J. The regulation of the secondary metabolism of Streptomyces: new links and experimental advances. *Nat. Prod. Rep.* **28**, 1311-1333, doi:10.1039/c1np00003a (2011).

14 Gomez-Escribano, J. P. & Bibb, M. J. Streptomyces coelicolor as an expression host for heterologous gene clusters. *Method. Enzymol.* **517**, 279-300, doi:10.1016/B978-0-12-404634-4.00014-0 (2012).

15 Yamada, Y., Cane, D. E. & Ikeda, H. Diversity and analysis of bacterial terpene synthases. *Method. Enzymol.* **515**, 123-162, doi:10.1016/B978-0-12-394290-6.00007-0 (2012).

16 Deering, R. W. *et al. N*-Acyl Dehydrotyrosines, Tyrosinase Inhibitors from the Marine Bacterium *Thalassotalea* sp. PP2-459. *J. Nat. Prod.* **79**, 447-450, doi:10.1021/acs.jnatprod.5b00972 (2016).

17 Berger, M., Neumann, A., Schulz, S., Simon, M. & Brinkhoff, T. Tropodithietic acid production in *Phaeobacter gallaeciensis* is regulated by *N*-acyl homoserine lactone-mediated quorum sensing. *J. Bacteriol.* **193**, 6576-6585, doi:10.1128/JB.05818-11 (2011).

18 Schlesner, H. The development of media suitable for the microorganisms morphologically resembling *Planctomyces* spp., *Pirellula* spp., and other

Planctomycetales from various aquatic habitats using dilute media. *Syst. Appl. Microbiol.* **17**, 135-145 (1994).
19 Lage, O. M. & Bondoso, J. Bringing Planctomycetes into pure culture. *Front. Microbiol.* **3**, 405, doi:10.3389/fmicb.2012.00405 (2012).
20 Jeske, O. *et al.* Planctomycetes do possess a peptidoglycan cell wall. *Nat. Commun.* **6**, 7116, doi:10.1038/ncomms8116 (2015).
