# Supplementary material for: The planctomycete Stieleria maiorica Mal15T employs stieleriacines to alter the species composition in marine biofilms
Source: Commun Biol. 2020 Jun 12;3:303. doi: 10.1038/s42003-020-0993-2 (PMC7293339; doi:10.1038/s42003-020-0993-2)
Supplement: Supplementary file 4 — Description of Additional Supplementary Files [file 42003_2020_993_MOESM4_ESM.pdf]

Description of Additional Supplementary Files

File Name: Supplementary Movie 1

Description: Time-lapse microscopy of dividing Mal15^T cells. Cells of strain Mal15^T were observed during their cell division cycle using the CellASIC® ONIX Microfluidic Platform. Elastically trapped cells were cultivated in M1H NAG ASW and supplemented with 3.3 µL acetone. Cells were monitored for 32 h at 28 °C. Images were taken every 10 min using phase contrast microscopy. Cells start to divide after 6.25 h in average, if no stieleriacine A_1 was applied to the experimental setting.

File Name: Supplementary Movie 2

Description: Time-lapse microscopy of dividing Mal15^T cells treated with 1.34 µM stieleriacine A_1. To analyse the physiological effect of stieleriacine A_1 under microfluidic conditions, Mal15^T cells were observed during their cell division cycle using the CellASIC® ONIX Microfluidic Platform. Elastically trapped cells were cultivated in M1H NAG ASW supplemented with 1.34 µM stieleriacine A_1 dissolved in 3.3 µL acetone. Cells were monitored for 32 h at 28 °C. Images were taken every 10 min using phase contrast microscopy. Mimicking a dense population by adding constantly physiological concentrations (1.34 µM) of stieleriacine A_1 leads to a significantly reduced lag phase compared to untreated cultures (5.16 h).
